# Global Monthly Water Scarcity: Blue Water Footprints versus Blue Water Availability

**DOI:** 10.1371/journal.pone.0032688

**Published:** 2012-02-29

**Authors:** Arjen Y. Hoekstra, Mesfin M. Mekonnen, Ashok K. Chapagain, Ruth E. Mathews, Brian D. Richter

**Affiliations:** 1 Department of Water Engineering and Management, University of Twente, Enschede, The Netherlands; 2 Water Footprint Network, Enschede, The Netherlands; 3 World Wide Fund-United Kingdom, Godalming, Surrey, United Kingdom; 4 The Nature Conservancy, Charlottesville, Virginia, United States of America; University of Oxford, United Kingdom

## Abstract

Freshwater scarcity is a growing concern, placing considerable importance on the accuracy of indicators used to characterize and map water scarcity worldwide. We improve upon past efforts by using estimates of blue water footprints (consumptive use of ground- and surface water flows) rather than water withdrawals, accounting for the flows needed to sustain critical ecological functions and by considering monthly rather than annual values. We analyzed 405 river basins for the period 1996–2005. In 201 basins with 2.67 billion inhabitants there was severe water scarcity during at least one month of the year. The ecological and economic consequences of increasing degrees of water scarcity – as evidenced by the Rio Grande (Rio Bravo), Indus, and Murray-Darling River Basins – can include complete desiccation during dry seasons, decimation of aquatic biodiversity, and substantial economic disruption.

## Introduction

The inexorable rise in demand for water to grow food, supply industries and sustain urban and rural populations has led to a growing scarcity of freshwater in many parts of the world. An increasing number of rivers now run dry before reaching the sea for substantial periods of the year. In many areas, groundwater is being pumped at rates that exceed replenishment, depleting aquifers and the base flows of rivers [1]. Increasingly, governments, corporations and communities are concerned about the future availability and sustainability of water supplies [2].

During the last twenty years, researchers have developed a number of metrics to help characterize, map and track the geography of water scarcity globally. These have included, for example, the ratio of population size to the renewable water supply [3] and the ratio of water withdrawals to the renewable supply [4]–[7]. These water scarcity indicators have highlighted the mismatch between water availability and water demand, and have helped document the spread of water scarcity over time. Today, water scarcity assessments underpin global assessments of food [7], poverty and human development [8], economic and business prospects [9], and ecological health [10]. Given this widespread use of water scarcity indicators, their accuracy is at a premium.

We have developed a new and more accurate assessment of global water scarcity by combining three innovations in measuring water use and availability. First, following recent developments in water use studies [11]–[17], we measure water use in terms of consumptive use of ground- and surface water flows – i.e., the blue water footprint – rather than water withdrawals. In agriculture, about 40% of water withdrawals typically return to local rivers and aquifers and thereby becomes available for reuse [18], [19], so that the volume of water consumed provides a more accurate basis for estimating scarcity than the volume of water withdrawn. In industries and households even 90–95% of the water withdrawn will return [20]. Second, in assessing water availability we take into account the flows needed to sustain critical ecological functions, as done earlier by for instance Smakhtin et al. [21]. We use a recently proposed presumptive standard that depletion beyond 20% of a river's natural flow increases risks to ecological health and ecosystem services [22]. Third, we compare water use and availability on a monthly rather than annual basis, similar to what Wada et al. [13] did recently. In this way we incorporate the often-great variability of water supply and use throughout the year and capture the seasonal nature of water scarcity [23]. Our global water scarcity study is the first to combine those three innovations in one assessment. It compares on a monthly basis the consumptive use component of blue water withdrawals to the estimated ecologically admissible fraction of runoff.

Following Hoekstra et al. [24], we define blue water scarcity in a given river basin as the ratio of the blue water footprint in that basin to the blue water available, where the latter accounts for environmental water needs by subtracting from the total runoff the presumed flow requirement for ecological health. As is the case in previous water scarcity indicators, we have focused on scarcity of water available in rivers and groundwater, or the “blue” water [25]; we do not consider scarcity of direct precipitation, or “green” water. Based on [26], the monthly blue water footprint of humanity was estimated at a five by five arc minute spatial resolution for the world as a whole, distinguishing between agricultural, industrial, and domestic water footprints. The blue water footprint of human activities is defined as the volume of surface and groundwater consumed as a result of that activity, whereby consumption refers to the volume of freshwater used and then evaporated or incorporated into a product. Natural runoff per river basin was estimated by taking estimates of actual runoff from Fekete et al. [27] and adding the water volumes already consumed (the blue water footprint). Blue water availability is estimated by reducing total natural runoff by 80% to account for presumed environmental flow requirements. The blue water availability is thus the volume of water that can be consumed without expected adverse ecological impacts. We hasten to note, however, that flows dedicated to the maintenance of ecological health can be used for other purposes; the presumptive standard is met as long as net depletion remains within 20% of the natural monthly flow.

We believe that our indicator provides a more reliable and accurate rendering of the status of water budgets (inputs minus outputs) at the river basin scale than has been available to date because it combines these three improvements over previous studies: use of water consumption instead of water withdrawal, explicit incorporation of environmental flow requirements and a monthly time-step. As such, this indicator provides decision-makers with an improved picture of where and when current levels of water use are likely to cause water shortages and ecological harm within river basins around the world.

## Methods

The blue water scarcity in a river basin is defined as the ratio of the total blue water footprint to the blue water availability in a river basin during a specific time period [24]. A blue water scarcity of one hundred per cent means that the available blue water has been fully consumed. The blue water scarcity is time-dependent; it varies within the year and from year to year. In this study, we calculate blue water scarcity per river basin on a monthly basis. Blue water footprint and blue water availability are expressed in mm/month. For each month of the year we consider the ten-year average for the period 1996–2005 to incorporate climate variability, while acknowledging that averaging can obscure inter-annual variability in scarcity.

Average monthly blue water footprints per river basin for the period 1996–2005 have been derived from the work of Mekonnen and Hoekstra [26], who estimated the global blue water footprint at a 5 by 5 arc minute spatial resolution. They reported annual values at country level, whereas in the current study we use the same underlying data to report monthly values at river basin level. The three primary water-consuming sectors are included: agriculture, industry and domestic water supply. The blue water footprint of crop production was calculated using a daily soil water balance model at the mentioned resolution level as reported in Mekonnen and Hoekstra [11], [28], [29]. Blue water consumption in irrigated crop production is calculated by performing two different soil water balance scenarios. The first soil water balance scenario is carried out based on the assumption that the soil does not receive any irrigation. The second soil water balance scenario is carried out with the assumption that the amount of actual irrigation is sufficient to meet the irrigation requirement, applying the same crop parameters as in the first scenario. The blue crop water consumption is equal to the crop water evapotranspiration over the growing period as simulated in the second scenario minus the total crop water evapotranspiration as estimated in the first scenario.

The blue water footprints of industries and domestic water supply were obtained by spatially distributing national data on industrial and domestic water withdrawals from the Food and Agricultural Organization of the United Nations (FAO) [20] according to population densities around the world as given by the Center for International Earth Science Information Network (CIESIN) and the International Center for Tropical Agriculture (CIAT) [30] and by assuming that 5% of the industrial withdrawals and 10% of the domestic withdrawals are ultimately consumed, i.e. evaporated, which are thought to be reasonable estimates according to FAO [20]. Due to a lack of data we have distributed the annual water consumption figures for industry and domestic use equally over the twelve months of the year without accounting for the possible monthly variation.

The monthly blue water availability in a river basin in a certain period was calculated as the ‘natural runoff’ in the basin minus ‘environmental flow requirement’. The natural runoff was estimated by adding the actual runoff and the total blue water footprint within the river basin. Monthly actual runoff data at a 30 by 30 arc minute resolution were obtained from the Composite Runoff V1.0 database [27]. These data are based on model estimates that were calibrated against runoff measurements for different periods, with the year 1975 as the mean central year. In order to approximate the natural (undepleted) runoff, we corrected the 1975 actual runoff data by adding the aggregated blue water footprint per basin as in 1975. The latter was estimated to be 74% of the blue water footprint per basin as was estimated by Mekonnen and Hoekstra [26] for the central year 2000. The 74% refers to the ratio of the global blue water footprint in 1975 to the global blue water footprint in 2000 [31].

In order to establish the environmental flow requirement we have adopted the “presumptive environmental flow standard” as proposed by Richter *et al.* [22] and Hoekstra *et al.* [24]. We note that the application of this standard does not imply that 80% of the total runoff is unavailable for use. In actuality all of the runoff can be used, as long as no more than 20% of the total runoff is depleted by water consumption. As suggested by Richter *et al.* [22], this presumptive standard is to be applied only when site-specific scientific investigation of environmental flow needs has not been undertaken. The presumptive standard is meant to be a precautionary approach to estimating environmental flow requirements when detailed local studies have not been completed, which is presently the case for the vast majority of the world's river basins. We acknowledge that governments and local stakeholders may intentionally choose to consume more than 20% of total natural runoff and bear the ecological consequences to gain other benefits associated with water consumption. However, we feel that it is very important to explicitly account for ecological health in water scarcity assessments, and use of this presumptive standard in the present study enables identification of river basins in which ecological health has likely been compromised.

Blue water scarcity values have been classified into four levels of water scarcity:

- low blue water scarcity (<100%): the blue water footprint is lower than 20% of natural runoff and does not exceed blue water availability; river runoff is unmodified or slightly modified; presumed environmental flow requirements are not violated.
- moderate blue water scarcity (100–150%): the blue water footprint is between 20 and 30% of natural runoff; runoff is moderately modified; environmental flow requirements are not met.
- significant blue water scarcity (150–200%): the blue water footprint is between 30 and 40% of natural runoff; runoff is significantly modified; environmental flow requirements are not met.
- severe water scarcity (>200%). The monthly blue water footprint exceeds 40% of natural runoff; runoff is seriously modified; environmental flow requirements are not met.

We evaluated 405 river basins, which together cover 66% of the global land area (excluding Antarctica) and represent 65% of the global population in 2000 (estimate based on CIESIN and CIAT [30]). We applied river basin boundaries and names as provided by Global Runoff Data Centre (GRDC) [32] (Figure S1). The land areas not covered include for example Greenland, the Sahara desert in North Africa, the Arabian peninsula, the Iranian, Afghan and Gobi deserts in Asia, the Mojave desert in North America and the Australian desert. Also excluded are many smaller land areas, often along the coasts, that do not fall within major river basins.

## Results

### Monthly blue water footprint

**Figure 1:**
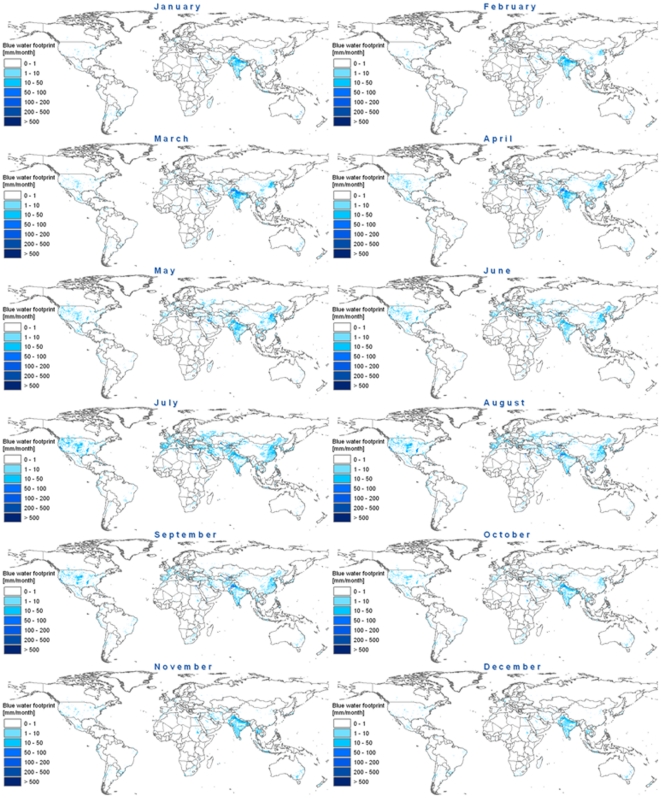
Monthly blue water footprint in the period 1996–2005. The data are shown in mm/month on a 5 by 5 arc minute grid. Data per grid cell have been calculated as the water footprint within a grid cell (in m^3^/month) divided by the area of the grid cell (in 10^3^ m^2^).

Agriculture accounts for 92% of the global blue water footprint; the remainder is equally shared between industrial production and domestic water supply [26]. However, the percentages of water consumed by agriculture, industry and domestic water supply vary across river basins and within the year. While the blue water footprint in agriculture varies from month to month depending on the timing and intensity of irrigation, the domestic water supply and industrial production were assumed to remain constant throughout the year. Therefore, for particular months in certain basins one hundred per cent of the blue water footprint can be attributed to industry and domestic water supply. The intra-annual variability of the total blue water footprint is mapped at a five by five arc minute grid in Figure 1. By aggregating the grid data to the level of river basins we obtain the maps as shown in Figure S2. The monthly blue water footprints per basin are further tabulated in Table S1. The values on the maps are shown in mm per month and can thus directly be compared.

A large blue water footprint throughout the year is observed for the Indus and Ganges River Basins, because irrigation occurs here throughout the year. A large blue water footprint during part of the year is estimated for basins such as the Tigris-Euphrates, Huang He (Yellow River), Murray-Darling, Guadiana, Colorado (Pacific Ocean) and Krishna. When we consider Europe and North America as a whole, we see a clear peak in the blue water footprint in the months May to September (around the northern summer). In Australia, we see a blue water footprint peak in the months October to March (around the southern summer). One cannot find such distinct seasonal patterns in the blue water footprint in South America, Africa or Asia, because these continents are more heterogeneous in climatic conditions.

### Monthly natural runoff and blue water availability by river basin

Natural runoff and blue water availability vary across basins and over the year as shown on the global maps in Figures S3, S4 and in Tables S2, S3. The Amazon and Congo River Basins together account for 28% of the natural runoff in the 405 river basins considered in this study. At a global level, monthly runoff is above average in the months of January and April to August and below average during the other months of the year. When we look at the runoff per region, we find that most of the runoff in North America occurs in the period of April to June, in Europe from March to June, in Asia between May and September, in Africa in January, August and September, and in South America from January to May. While the Amazon and Congo River Basins display relatively low variability over the year, much sharper gradients are apparent in other basins. In some parts of the world, a large portion of the annual runoff occurs within a few weeks or months, generating floods during one part of the year and drought during the other part. Even in otherwise water abundant areas, intra-annual variability can severely limit blue water availability. Under such conditions, considering blue water availability on an annual basis provides an incomplete and sometimes misleading view of blue water availability per basin.

### Monthly water scarcity by river basin

**Figure 2:**
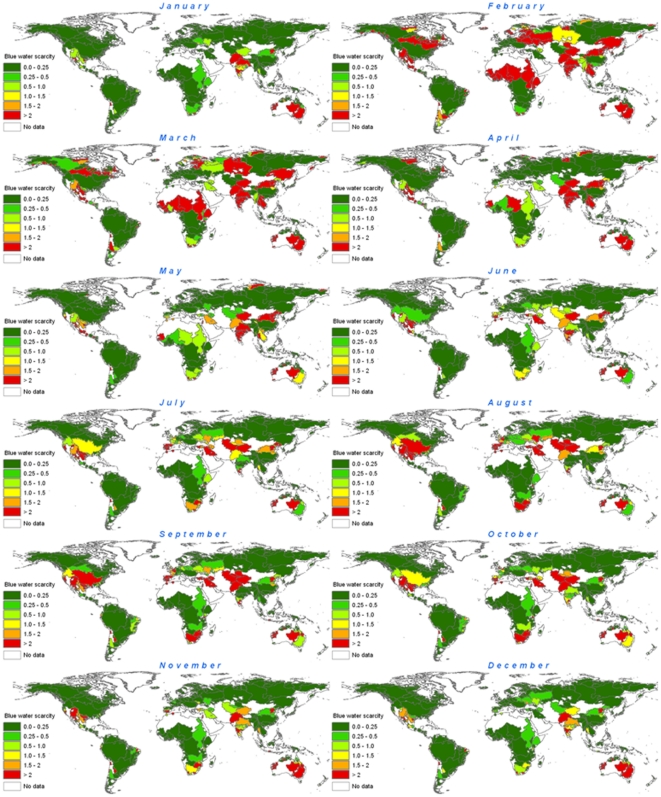
Monthly water scarcity in the world's major river basins, based on the period of 1996–2005. In each month that a river basin is colored in some shade of green, the monthly water scarcity is low (blue water footprint is less than net availability). In such cases, the presumed environmental flow requirements are not violated, and river runoff in that month is unmodified or only slightly modified. In each month that a river basin is colored yellow, water scarcity is moderate. Blue water footprint is between 20 and 30% of natural runoff; runoff is hence moderately modified and environmental flow requirements are not fully met. When a river basin is colored orange, water scarcity is significant. Blue water footprint is between 30 and 40% of natural runoff, so monthly runoff is significantly modified. In each month that a river basin is colored red, water scarcity is severe; the blue water footprint exceeds 40% of natural runoff, therefore runoff is seriously modified.

**Figure 3:**
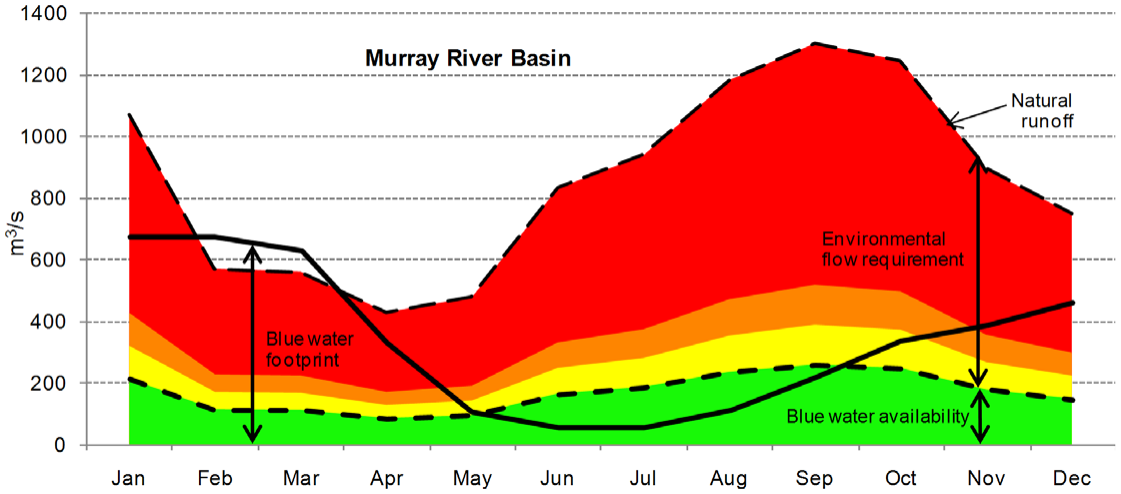
Water scarcity over the year for the Murray-Darling River Basin in Australia (average for the period 1996–2005). Net available water – that is natural runoff minus environmental flow requirement – is shown in green. From October until May, the blue water footprint exceeds net available water; in these months, the presumptive environmental flow requirement is not met. When the blue water footprint moves into the yellow, orange and red colors, water scarcity is moderate, significant and severe, respectively.

For this assessment, we analyzed 405 river basins that collectively account for 69 percent of global runoff, 75 percent of world irrigated area, and 65 percent of world population. For each river basin and each month, we categorize water scarcity from low to severe based on the ratio of blue water footprint to blue water availability (natural runoff minus environmental flow requirements). Referring to Figure 2, in river basins shown in green in a given month, the blue water footprint is less than 20 percent of that month's natural runoff. There is little or no water scarcity and the basin fully meets that month's presumptive environmental flow requirement. Data are provided in Table S4. We illustrate the relationships between blue water footprint, natural runoff, environmental flow requirements and blue water availability for the Murray-Darling River Basin in Figure 3. One can see that blue water footprint in the Murray-Darling River Basin is largest in the period that water availability is lowest. The blue water footprint exceeds natural runoff during a part of the dry period, which is made possible through temporary depletion of groundwater or surface water reservoir storage.

Table 1 gives an overview of the number of basins and number of people facing low, moderate, significant and severe water scarcity during a given number of months per year. In 223 river basins (55% of the basins studied) with 2.72 billion inhabitants (69% of the total population living in the basins included in this study), the blue water footprint exceeds blue water availability during at least one month of the year. For 201 of these basins, with together 2.67 billion inhabitants, there was severe water scarcity during at least one month of the year, highlighting the fact that when water scarcity exists it is usually of a severe nature, meaning that more than 40% of natural runoff is being consumed. In 35 river basins with 483 million people, there was severe water scarcity for at least half of the year.

**Table 1:** Number of basins and number of people facing low, moderate, significant and severe water scarcity during a given number of months per year.

	Number of basins facing low, moderate, significant and severe water scarcity during *n* months per year				Number of people (millions) facing low, moderate, significant and severe water scarcity during *n* months per year			
Number of months per year (*n*)	Low water scarcity	Moderate water scarcity	Significant water scarcity	Severe water scarcity	Low water scarcity	Moderate water scarcity	Significant water scarcity	Severe water scarcity
0	17	319	344	204	353	2690	2600	1289
1	2	55	45	46	18.6	894	357	440
2	1	26	12	49	0.002	302	672	512
3	4	4	2	33	79.6	69.2	220	182
4	6	1	1	22	35.0	0.14	9.2	345
5	18	0	1	16	897	0	97.8	706
6	9	0	0	10	111	0	0	25.6
7	17	0	0	4	144	0	0	88.0
8	29	0	0	4	293	0	0	254
9	29	0	0	3	66.8	0	0	20.2
10	52	0	0	0	428	0	0	0
11	39	0	0	2	296	0	0	1.8
12	182	0	0	12	1233	0	0	93.3
Total	405	405	405	405	3956	3956	3956	3956

**Figure 4:**
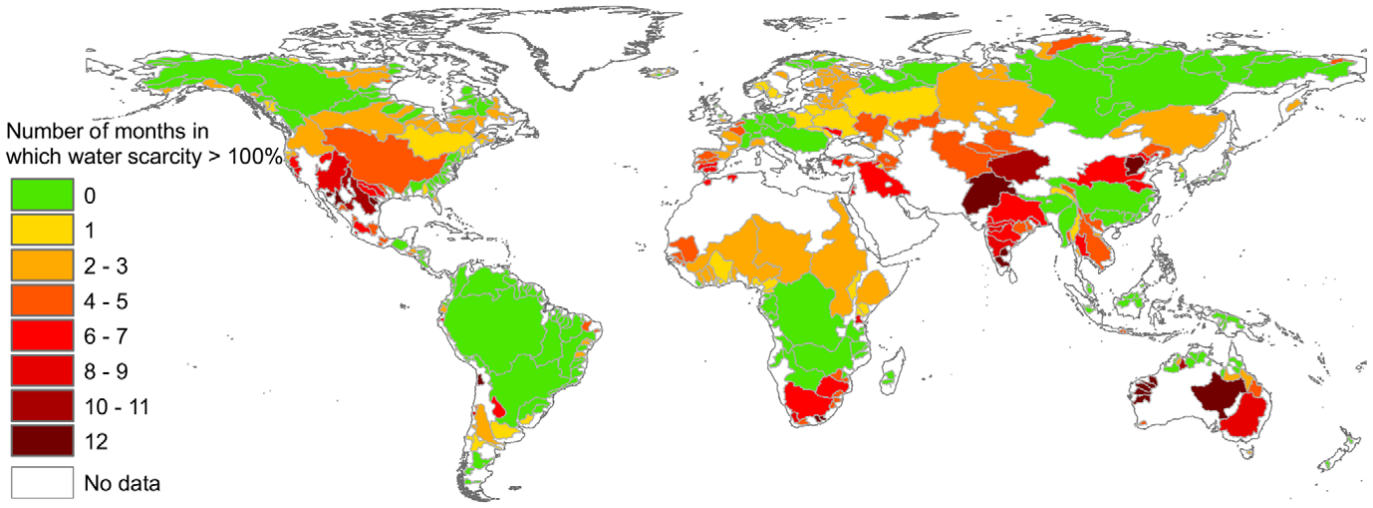
Number of months during the year in which the blue water footprint exceeds blue water availability for the world's major river basins, based on the period of 1996–2005. Blue water availability refers to natural flows (through rivers and groundwater) minus the presumed environmental flow requirement.

Of importance when considering the social, economic and environmental impacts of water scarcity is both the severity and the duration of the scarcity (see Figure 4). Twelve of the river basins included in this study experience severe water scarcity during all months of the year. The largest of those basins is the Eyre Lake Basin in Australia, one of the largest endorheic basins in the world, arid and inhabited by only about 86,000 people, but covering around 1.2 million km^2^. The most heavily populated basin facing severe water scarcity all year long is the Yongding He Basin in northern China (serving water to Beijing), with an area of 214,000 km^2^ and a population density of 425 persons per km^2^. Eleven months of severe water scarcity occurs in the San Antonio River Basin in Texas, US and the Groot-Kei River Basin in Eastern Cape, South Africa. Two heavily populated river basins face nine months of severe water scarcity, the Penner River Basin in southern India, a basin with a dry tropical monsoon climate and 10.9 million people, and the Tarim River Basin in China, which includes the Taklamakan Desert with 9.3 million people. Four basins face severe water scarcity during eight months a year: the Indus with 212 million people; the Cauvery with an area of 91,000 km^2^ and 35 million people; the Dead Sea Basin, which includes the Jordan River and extends over parts of Jordan, Israel, the West Bank and minor parts of Lebanon and Egypt; and the Salinas River in California in the US.

## Discussion

The current study provides the first global assessment of blue water scarcity at the scale of river basins and at a monthly resolution while accounting for environmental flow requirements. We find that at least 2.7 billion people are living in basins that experience severe water scarcity during at least one month of the year. Our estimate is close to what Oki and Kanae [5] found in another recent global water scarcity study, although they looked at water withdrawals instead of consumption and considered water scarcity at an annual basis. They found 2.4 billion people living in severely water-stressed areas. The similar finding is explained by the fact that Oki and Kanae call an area ‘severely water stressed’ already when the annual ratio of water withdrawal to runoff exceeds 40% [5]. When we roughly assume that water consumption (the blue water footprint) is 60% of total water withdrawal in a basin, this criterion is equivalent to saying that severe water stress occurs when the blue water footprint exceeds 24% of runoff, which means that less than 76% of runoff remains (on an annual basis). In our study, severe water scarcity is assumed to occur when less than 60% of runoff remains (on a monthly basis). We thus use a less strict criterion, but apply a monthly evaluation which is more strict. This can help explain the similarity between [5] and our study in the identification of severely water stressed areas and in the estimation of the number of people living under severe water stress.

However, water scarcity analysis at a monthly time step provides insight into water scarcity that is not revealed in annual water scarcity studies [4]–[6], [21]; in particular the fact that scarcity occurs in certain periods of the year and not in others [13], [33]. This enables a more detailed analysis of when water consumption is exceeding water availability which can assist in pinpointing and prioritizing investments in blue water footprint reduction. If stricter criteria for high water scarcity was used in line with previous annual studies, the number of high water stress areas and the people affected by water stress would increase.

In this study, water scarcity has been evaluated at the scale of large river basins. Other investigators have presented global water scarcity assessments at a much higher spatial resolution, by applying a 30 arc minute grid [5]–[6], [13]. While we acknowledge that portrayal of water scarcity at a higher spatial resolution can be useful for some purposes, we feel that it is very important to portray water scarcity using geographic units familiar and relevant to water managers and planners, i.e., at the river basin scale. We also caution that the accuracy of existing runoff and water consumption data may not yet warrant interpretation of results at higher spatial resolution. We stress that our basic analyses of blue water footprint and water availability have been carried out at high-resolution grid level, so that it is only in the presentation of scarcity levels that we show results at basin level.

The levels of water scarcity estimated in this study correspond strongly with documented ecological declines and socio-economic disruption in some of the world's most heavily used river basins. The Indus River Basin, with 212 million people, faces severe water scarcity during eight months of the year. In the northwestern Indian provinces of Punjab, Rajasthan and Haryana, each one of which lies fully or partly in the Indus River Basin, groundwater is steadily being depleted [34]. Unsustainable groundwater depletion and severe water scarcity threaten potable water supplies and agricultural output, affecting the country's food supplies and the government's welfare programs. The Rio Grande (or Rio Bravo) Basin – an international river basin shared by the US and Mexico – suffers severe water scarcity during seven months of the year. As a result of low water levels, the concentration of pollutants is so high that fish kills have occurred, and the lower river is suffering from greatly increased salinity levels which have displaced 32 native freshwater fish species [35]. Regional economic losses in irrigated agriculture due to water shortages have been estimated at $135 million per year, including loss of more than 4,000 jobs annually [36]. In the Murray-Darling basin in south-eastern Australia with six months of severe water scarcity, depletion of river flows caused the Murray to run dry before reaching the sea for the first time in 2002, and 20 of 23 sub-basins have been assessed as being in “poor” to “very poor” ecosystem health [37]. A highly controversial new draft basin plan proposes a multi-billion dollar government program of irrigation water buybacks in an attempt to reduce consumption by at least 20% and increase return flows to depleted wetlands and streams, with projected economic losses to agriculture of at least $800 million per year [37].

With severe water scarcity occurring at least one month per year in close to one half of the river basins included in this study, our results underline the critical nature of water shortages around the world. Businesses, investors, farmers, governments and others may find this scarcity indicator useful in assessing their water-related risks. The indicator highlights where investments in improved water efficiency and productivity may be critical to averting water shortages and seasonal rationing. It also illuminates that trade – particularly in agricultural products – can help alleviate water scarcity through import of water-intensive products from more water-rich areas.

Rockström *et al.* [38] have posed that planetary boundaries for different global resources can be determined. By including the presumptive environmental flow requirement and doing the analysis at a monthly time-step, our water scarcity indicator contributes higher resolution analysis for setting a boundary for the sustainable use of freshwater at local and regional scales [39], [40]. Maintaining water use within this boundary of water availability can have implications for economic and infrastructure planning, trade and agricultural policies, and development aid. The presumptive environmental flow standard applied in our water scarcity analysis is a precautionary boundary that should be refined with site-specific studies. However, depletion beyond this boundary will typically involve tradeoffs between the social and economic benefits of increased consumptive use and the loss of ecosystem health and related social and economic costs [22].

While our water scarcity indicator provides an improved accounting of the current status of basin water budgets, a couple of caveats deserve mention so as to avoid misinterpretation of these results. Our estimates of blue water availability account for month-by-month natural variability in flow, but they do not yet properly account for the perturbation of seasonal runoff patterns by river flow regulation by dams. The runoff dataset from Fekete et al. [27] used in this study is a construct based on runoff modeling on the one hand and river discharge measurements on the other hand, so that it *implicitly* includes impacts from reservoirs, inter-basin transfers and consumptive water use (but only in those cases where discharge measurements were available). We have nullified the impact of consumptive water use by adding our own consumptive water use estimates to the ‘actual’ runoff from this dataset to obtain ‘natural’ runoff, but we have not been able to cancel out the effects of dams and inter-basin transfers.

Further, our water footprint estimates do not yet include evaporation from artificial reservoirs. Additionally, our estimates of blue water footprint do not account for inter-basin transfers of water. For basins that are net exporters of water (e.g., the Colorado, through deliveries to southern California, Las Vegas, the Front Range of Colorado and elsewhere) the scarcity picture is likely worse than presented here, whereas for net importers of water it may be better.

Our water scarcity estimates also include uncertainties inherent in the data used and the assumptions made. The data on actual runoff are model-based estimates calibrated against long-term runoff measurements [27]; the model outcomes include an error of 5% at the scale of large river basins and greater in smaller basins. The runoff measurements against which the model is calibrated have accuracy on the order of ±10–20 percent [27]. Estimates of blue water footprint can easily contain an uncertainty of ±20% [28], [29], [41]; in general, uncertainties for relatively small river basins will be bigger than for large river basins.

In order to estimate natural (undepleted) runoff in each river basin, we have added the estimated blue water footprint from [26] to the estimated actual runoff from [27]. In doing so, we overestimate natural runoff in those months in which the blue water footprint partially draws down the total annual water storage in the basin (e.g., from aquifers) rather than depleting that month's runoff. Similarly, we underestimate the natural runoff in the months in which water is being stored for later consumption. Further, as a result of our approach we overestimate natural runoff in those months and basins in which a portion of the water consumed comes from fossil (non-renewable) groundwater, because that water should not be included in natural runoff. However, empirical data on consumption of renewable versus fossil groundwater are very difficult to obtain at a global scale; so far only rough assessments based on models and assumptions have been made [12], [42], [43].

This study has excluded the issue of water pollution. Blue water scarcity has been defined such that it refers to scarcity in quantitative sense. Return flows from agriculture, industries and households are not consumptive use, so they do not affect our scarcity measure. In many places, water scarcity is much higher than suggested by us if one would consider scarcity of *uncontaminated* water.

Despite these cautionary notes, our estimates provide a significant improvement over previous water scarcity indicators and the relative spatial and temporal patterns of water scarcity globally because they provide a more detailed assessment of when and where water scarcity occurs. Moreover, the calculated scarcity values for each river basin and month are conservative estimates of actual scarcity for two reasons. First, by evaluating water scarcity at the level of whole river basins, we do not capture spatial variations within basins. Flows may be substantially more depleted at the sub-basin level, for example, than for that basin as a whole. Second, we assume an average year with regard to both blue water footprint and availability, but in many basins inter-annual variations are substantial, aggravating the scarcity problem in the drier years.

The water scarcity values presented refer to the period 1996–2005. Continued growth in blue water footprint due to growing populations, changing food patterns (for instance, more meat consumption) and increasing demand for biofuels, combined with the effects of climate change on runoff patterns, are likely to result in a worsening and expansion of water scarcity in many river basins in the decades ahead [6].

## Supporting Information

Figure S1 **Global river basin map.**
(TIFF)

Figure S2 **Global maps of the monthly blue water footprint in the world's major river basins. Period 1996–2005.**
(TIF)

Figure S3 **Global maps of monthly natural runoff in the world's major river basins.**
(TIF)

Figure S4 **Global maps of monthly blue water availability in the world's major river basins.**
(TIF)

Table S1 **Monthly blue water footprint for the world's major river basins.**
(PDF)

Table S2 **Monthly natural runoff for the world's major river basins.**
(PDF)

Table S3 **Monthly blue water availability for the world's major river basins.**
(PDF)

Table S4 **Monthly blue water scarcity for the world's major river basins.**
(PDF)
