# Supplementary figures and images for: Global Monthly Water Scarcity: Blue Water Footprints versus Blue Water Availability
Source: PLoS One. 2012 Feb 29;7(2):e32688. doi: 10.1371/journal.pone.0032688 (PMC3290560; doi:10.1371/journal.pone.0032688)

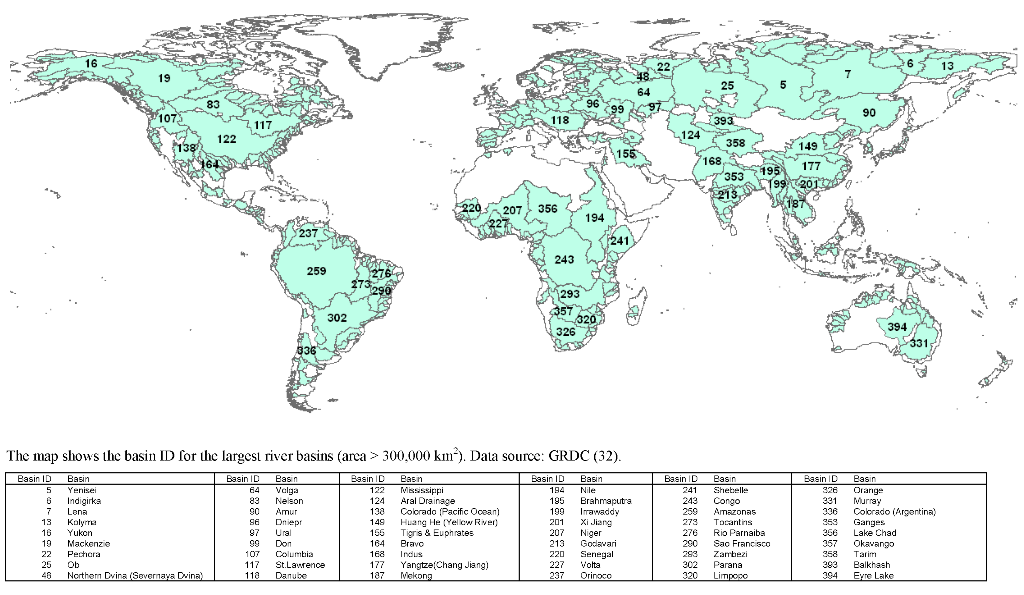

Supplement: Figure S1 — Global river basin map. (TIFF) [file pone.0032688.s001.tif]

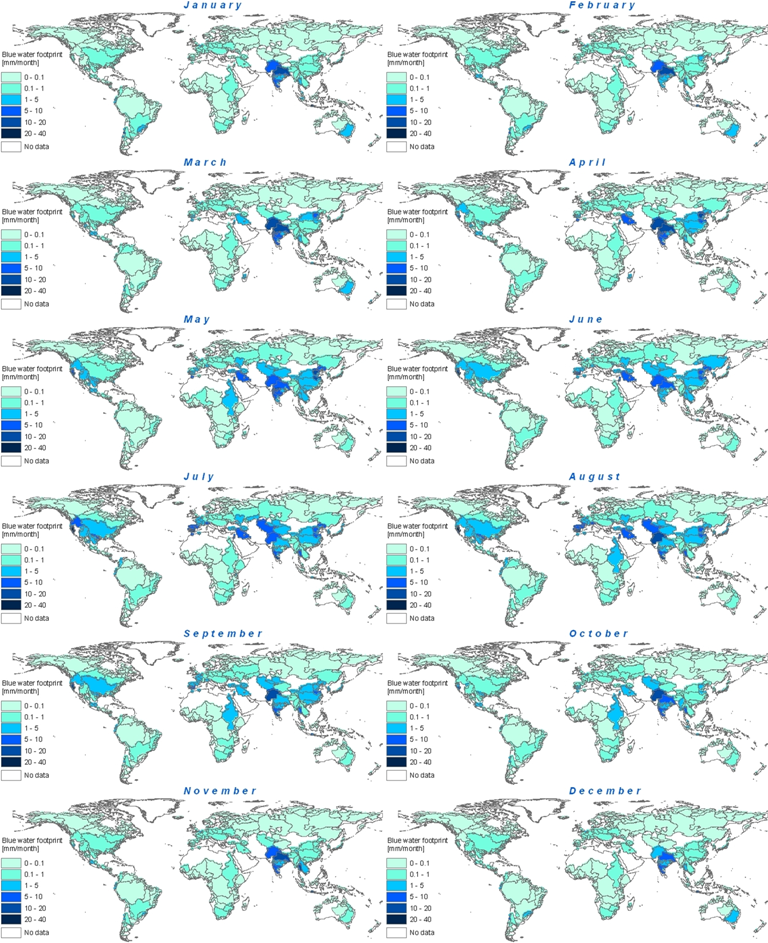

Supplement: Figure S2 — Global maps of the monthly blue water footprint in the world's major river basins. Period 1996–2005. (TIF) [file pone.0032688.s002.tif]

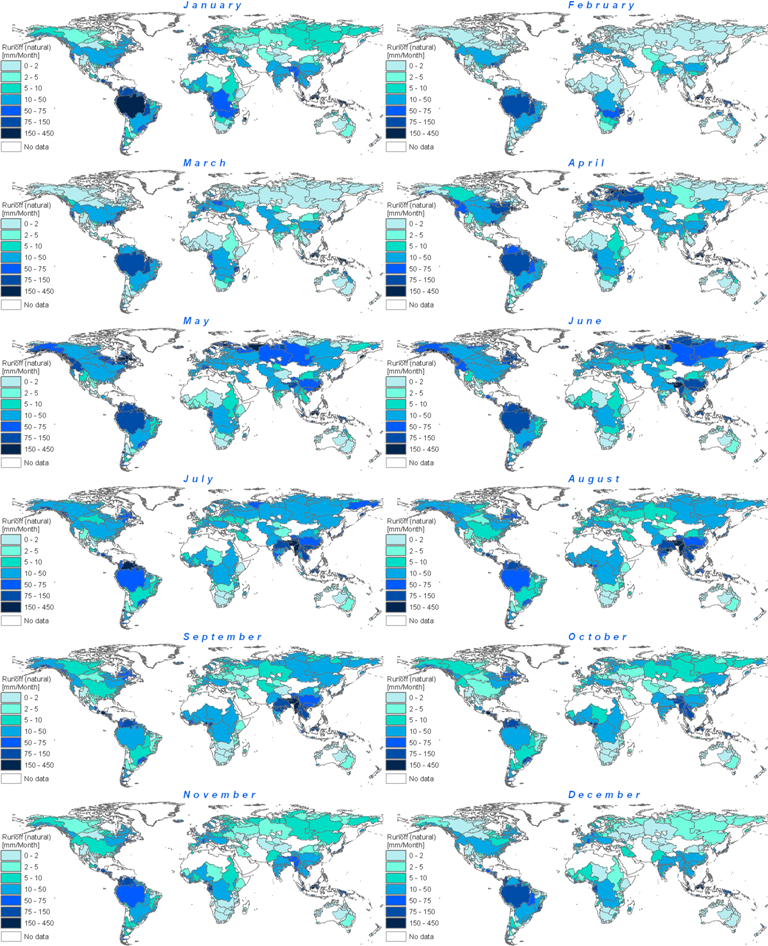

Supplement: Figure S3 — Global maps of monthly natural runoff in the world's major river basins. (TIF) [file pone.0032688.s003.tif]

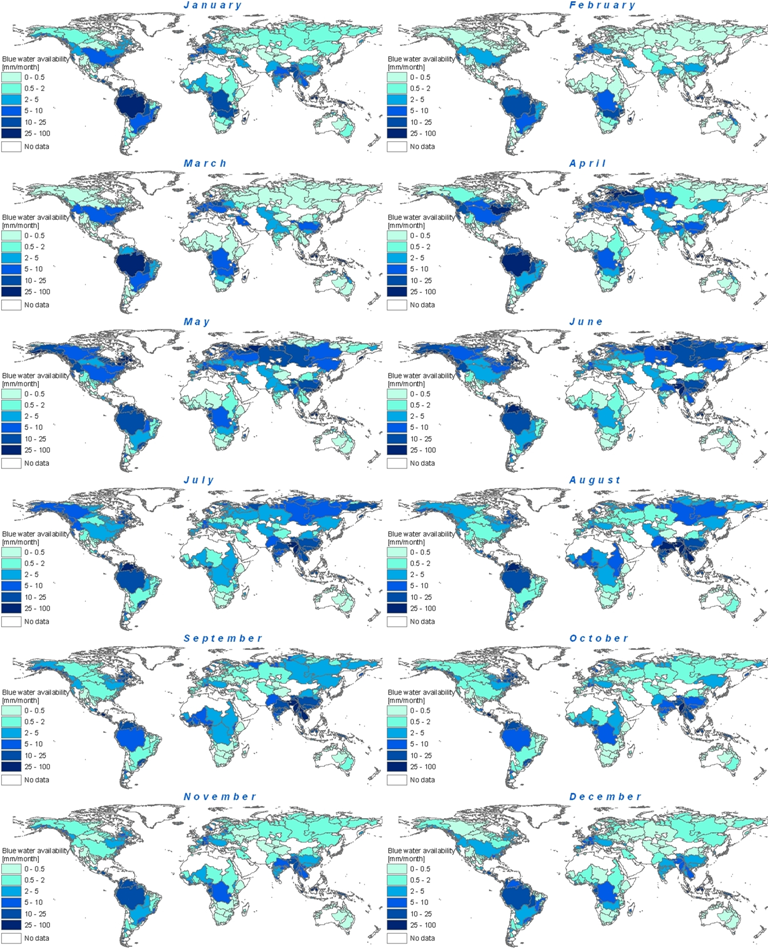

Supplement: Figure S4 — Global maps of monthly blue water availability in the world's major river basins. (TIF) [file pone.0032688.s004.tif]
