# Supplementary material for: Global Monthly Water Scarcity: Blue Water Footprints versus Blue Water Availability
Source: PLoS One. 2012 Feb 29;7(2):e32688. doi: 10.1371/journal.pone.0032688 (PMC3290560; doi:10.1371/journal.pone.0032688)
Supplement: Table S1 — Monthly blue water footprint for the world's major river basins. (PDF) [file pone.0032688.s005.pdf]

Table S1. Monthly blue water footprint for the world's major river basins

Period: 1996-2005

Basin ID	Basin name	Blue water footprint (10^3 m^3/month)												
		Jan	Feb	Mar	Apr	May	Jun	Jul	Aug	Sep	Oct	Nov	Dec	Average
1	Khatanga	8.8	8.8	8.8	8.8	8.8	8.8	8.8	8.8	8.8	8.8	8.8	8.8	8.8
2	Olenek	11.3	11.3	11.3	11.3	11.3	11.3	11.3	11.3	11.3	11.3	11.3	11.3	11.3
3	Anabar	2.6	2.6	2.6	2.6	2.6	2.6	2.6	2.6	2.6	2.6	2.6	2.6	2.6
4	Yana	46.4	46.4	46.4	46.4	46.4	46.4	46.4	46.4	46.4	46.4	46.4	46.4	46.4
5	Yenisei	14005.0	14005.0	14010.8	22586.1	67379.7	87527.4	79042.8	56657.6	32477.7	18324.4	14275.6	14012.4	36192.1
6	Indigirka	79.1	79.1	79.1	79.1	79.1	79.1	79.1	79.1	79.1	79.1	79.1	79.1	79.1
7	Lena	2433.3	2433.3	2433.3	2433.4	2434.3	2436.8	2447.0	2468.5	2445.9	2433.4	2433.3	2433.3	2438.8
8	Omoloy	5.5	5.5	5.5	5.5	5.5	5.5	5.5	5.5	5.5	5.5	5.5	5.5	5.5
9	Tana (NO, FI)	14.6	14.6	14.6	14.6	14.6	14.6	14.6	14.6	14.6	14.6	14.6	14.6	14.6
10	Colville	5.0	5.0	5.0	5.0	5.0	5.0	5.0	5.0	5.0	5.0	5.0	5.0	5.0
11	Alazeya	12.6	12.6	12.6	12.6	12.6	12.6	12.6	12.6	12.6	12.6	12.6	12.6	12.6
12	Anderson	0.6	0.6	0.6	0.6	0.6	0.6	0.6	0.6	0.6	0.6	0.6	0.6	0.6
13	Kolyma	261.7	261.7	261.7	261.7	261.7	262.0	262.3	264.3	262.8	261.7	261.7	261.7	262.1
14	Tuloma	397.5	397.5	397.5	397.5	397.5	397.5	397.5	397.5	397.5	397.5	397.5	397.5	397.5
15	Muonio	110.0	110.0	110.0	110.0	110.0	110.0	110.0	110.0	110.0	110.0	110.0	110.0	110.0
16	Yukon	709.7	709.7	709.7	733.2	864.6	869.9	819.9	756.4	751.8	725.8	712.9	710.3	756.2
17	Palyavaam	14.8	14.8	14.8	14.8	14.8	14.8	14.8	14.8	14.8	14.8	14.8	14.8	14.8
18	Kemijoki	322.5	322.5	322.5	322.5	322.5	322.5	322.5	322.5	322.5	322.5	322.5	322.5	322.5
19	Mackenzie	3302.6	3302.6	3302.7	3524.1	3876.2	3757.9	3650.4	3685.7	3512.4	3419.4	3324.3	3302.9	3496.8
20	Noatak	10.3	10.3	10.3	10.3	10.3	10.3	10.3	10.3	10.3	10.3	10.3	10.3	10.3
21	Anadyr	21.3	21.3	21.3	21.3	21.3	21.3	21.3	21.3	21.3	21.3	21.3	21.3	21.3
22	Pechora	1147.7	1147.7	1147.7	1147.7	1147.7	1147.7	1147.7	1147.7	1147.7	1147.7	1147.7	1147.7	1147.7
23	Lule	64.2	64.2	64.2	64.2	64.2	64.2	64.2	64.2	64.2	64.2	64.2	64.2	64.2
24	Kalixaelven	62.1	62.1	62.1	62.1	62.1	62.1	62.1	62.1	62.1	62.1	62.1	62.1	62.1
25	Ob	55630.5	55630.5	55641.7	95861.9	304570.9	399138.7	534705.8	460741.0	242699.8	102971.4	57227.1	55632.2	201704.3
26	Ellice	0.0	0.0	0.0	0.0	0.0	0.0	0.0	0.0	0.0	0.0	0.0	0.0	0.0
27	Taz	28.6	28.6	28.6	28.6	28.6	28.6	28.6	28.6	28.6	28.6	28.6	28.6	28.6
28	Kobuk	11.1	11.1	11.1	11.1	11.1	11.1	11.1	11.1	11.1	11.1	11.1	11.1	11.1
29	Coppermine	2.9	2.9	2.9	2.9	2.9	2.9	2.9	2.9	2.9	2.9	2.9	2.9	2.9
30	Hayes(Trib. Arctic Ocean)	0.0	0.0	0.0	0.0	0.0	0.0	0.0	0.0	0.0	0.0	0.0	0.0	0.0
31	Pur	372.7	372.7	372.7	372.7	372.7	372.7	372.7	372.7	372.7	372.7	372.7	372.7	372.7
32	Varzuga	7.8	7.8	7.8	7.8	7.8	7.8	7.8	7.8	7.8	7.8	7.8	7.8	7.8
33	Ponoy	6.5	6.5	6.5	6.5	6.5	6.5	6.5	6.5	6.5	6.5	6.5	6.5	6.5
34	Kovda	62.8	62.8	62.8	62.8	62.8	62.8	62.8	62.8	62.8	62.8	62.8	62.8	62.8
35	Back	0.1	0.1	0.1	0.1	0.1	0.1	0.1	0.1	0.1	0.1	0.1	0.1	0.1
36	Kem	147.8	147.8	147.8	147.8	147.8	147.8	147.8	147.8	147.8	147.8	147.8	147.8	147.8
37	Nadym	82.7	82.7	82.7	82.7	82.7	82.7	82.7	82.7	82.7	82.7	82.7	82.7	82.7
38	Quoich	0.0	0.0	0.0	0.0	0.0	0.0	0.0	0.0	0.0	0.0	0.0	0.0	0.0
39	Mezen	79.7	79.7	79.7	79.7	79.7	79.7	79.7	79.7	79.7	79.7	79.7	79.7	79.7
40	Iijoki	138.9	138.9	138.9	138.9	139.0	139.1	139.1	139.6	139.2	139.0	138.9	138.9	139.0
41	Joekulsa A Fjoellum	2.3	2.3	2.3	2.3	2.3	2.3	2.3	2.3	2.3	2.3	2.3	2.3	2.3
42	Svarta, Skagafiroi	6.1	6.1	6.1	6.1	6.1	6.1	6.1	6.1	6.1	6.1	6.1	6.1	6.1
43	Oulujoki	435.0	435.0	435.0	435.0	445.6	464.0	490.9	525.1	480.8	440.8	435.0	435.0	454.7
44	Lagarfljot	9.0	9.0	9.0	9.0	9.0	9.0	9.0	9.0	9.0	9.0	9.0	9.0	9.0
45	Thelon	14.5	14.5	14.5	14.5	14.5	14.5	14.5	14.5	14.5	14.5	14.5	14.5	14.5
46	Angerman	117.4	117.4	117.4	117.4	117.4	117.4	117.4	117.4	117.4	117.4	117.4	117.4	117.4
47	Thjorsa	5.5	5.5	5.5	5.5	5.5	5.5	5.5	5.5	5.5	5.5	5.5	5.5	5.5
48	Northern Dvina(Severnaya I	3254.5	3254.5	3254.5	3256.1	3650.4	3924.0	3905.7	3715.4	3340.4	3254.5	3254.5	3254.5	3443.2
49	Oelfusa	22.4	22.4	22.4	22.4	22.4	22.4	22.4	22.4	22.4	22.4	22.4	22.4	22.4
50	Nizhny Vyg (Soroka)	164.6	164.6	164.6	164.6	164.6	164.6	164.6	164.6	164.6	164.6	164.6	164.6	164.6
51	Kuskokwim	57.6	57.6	57.6	57.6	59.3	59.5	58.4	57.6	57.6	57.6	57.6	57.6	58.0
52	Vuoksi	1650.8	1650.8	1650.8	1650.8	1715.7	1799.9	1953.7	2144.8	1886.8	1667.7	1650.8	1650.8	1756.1
53	Onega	333.5	333.5	333.5	333.5	381.5	426.0	423.5	383.5	338.8	333.5	333.5	333.5	357.3
54	Susitna	152.5	152.5	152.5	153.5	187.7	189.8	163.9	153.7	152.6	152.6	152.5	152.5	159.7
55	Kymijoki	1285.5	1285.5	1285.5	1285.5	1364.7	1421.1	1488.1	1625.0	1456.9	1296.9	1285.5	1285.5	1363.8
56	Neva	8027.9	8027.9	8027.9	8045.8	10574.5	12074.7	11011.0	11704.2	8871.9	8031.5	8027.9	8027.9	9204.4
57	Ferguson	0.0	0.0	0.0	0.0	0.0	0.0	0.0	0.0	0.0	0.0	0.0	0.0	0.0
58	Copper	24.9	24.9	24.9	24.9	24.9	24.9	24.9	24.9	24.9	24.9	24.9	24.9	24.9
59	Gloma	1728.4	1728.4	1728.4	1729.2	1903.1	2987.3	4253.9	4640.8	2178.8	1728.5	1728.4	1728.4	2338.6
60	Kokemaenjoki	1710.1	1710.1	1710.1	1710.1	1914.1	2077.2	2256.1	2597.0	2145.7	1723.1	1710.1	1710.1	1914.5
61	Vaenern-Goeta	2650.5	2650.5	2650.5	2652.1	2858.8	3208.6	3451.6	3187.7	2747.6	2651.0	2650.5	2650.5	2834.2
62	Thlewiaza	0.3	0.3	0.3	0.3	0.3	0.3	0.3	0.3	0.3	0.3	0.3	0.3	0.3
63	Alsek	6.6	6.6	6.6	6.6	6.6	6.6	6.6	6.6	6.6	6.6	6.6	6.6	6.6
64	Volga	116047.3	116047.3	116127.0	151487.2	607847.6	798852.7	1124796	963030.8	356041.0	162975.0	120668.5	116099.0	395835.0
65	Dramselv	642.5	642.5	642.5	642.5	654.1	826.0	1109.7	905.7	702.3	642.5	642.5	642.5	724.6
66	Arnaud	0.0	0.0	0.0	0.0	0.0	0.0	0.0	0.0	0.0	0.0	0.0	0.0	0.0
67	Nushagak	7.4	7.4	7.4	7.4	7.4	7.4	7.4	7.4	7.4	7.4	7.4	7.4	7.4
68	Seal	7.2	7.2	7.2	7.2	7.2	7.2	7.2	7.2	7.2	7.2	7.2	7.2	7.2
69	Taku	11.8	11.8	11.8	11.8	11.8	11.8	11.8	11.8	11.8	11.8	11.8	11.8	11.8
70	Narva	1601.6	1601.6	1601.6	1604.9	1893.4	1940.6	2045.5	2297.4	1791.2	1607.8	1601.6	1601.6	1765.7
71	Stikine	10.0	10.0	10.0	10.0	10.0	10.0	10.0	10.0	10.0	10.0	10.0	10.0	10.0
72	Churchill	605.0	605.0	605.1	680.2	814.0	750.3	759.1	763.2	704.7	665.2	616.8	605.6	681.2
73	Feuilles (Riviere Aux)	0.0	0.0	0.0	0.0	0.0	0.0	0.0	0.0	0.0	0.0	0.0	0.0	0.0
74	George	0.2	0.2	0.2	0.2	0.2	0.2	0.2	0.2	0.2	0.2	0.2	0.2	0.2
75	Caniapiscau	6.2	6.2	6.2	6.2	6.2	6.2	6.2	6.2	6.2	6.2	6.2	6.2	6.2
76	Western Dvina (Daugava)	2902.1	2902.1	2902.1	3089.8	4952.7	4524.9	4328.4	4954.4	3556.7	2913.7	2902.1	2902.1	3569.3
77	Aux Melezes	0.0	0.0	0.0	0.0	0.0	0.0	0.0	0.0	0.0	0.0	0.0	0.0	0.0
78	Baleine, Grande Riviere De	0.0	0.0	0.0	0.0	0.0	0.0	0.0	0.0	0.0	0.0	0.0	0.0	0.0
79	Spey	26.4	26.4	26.4	26.4	26.4	26.4	26.4	26.4	26.4	26.4	26.4	26.4	26.4
80	Kamchatka	48.9	48.9	48.9	48.9	48.9	48.9	48.9	48.9	49.0	48.9	48.9	48.9	48.9
81	Nass	19.9	19.9	19.9	19.9	19.9	19.9	19.9	19.9	19.9	19.9	19.9	19.9	19.9
82	Skeena	300.1	300.1	300.1	300.1	300.1	300.1	300.1	300.1	300.1	300.1	300.1	300.1	300.1
83	Nelson	36043.3	36119.7	37077.4	98166.3	181129.3	204530.0	355878.4	533170.5	281797.3	108523.6	55374.7	39680.0	163957.5
84	Hayes(Trib. Hudson Bay)	96.9	96.9	96.9	96.9	96.9	96.9	96.9	96.9	96.9	96.9	96.9	96.9	96.9
85	Gudena	400.5	400.5	400.5	402.4	1175.8	2941.0	3637.7	1889.1	1014.8	415.5	400.5	400.5	1123.2
86	Skjern A	144.1	144.1	144.1	144.4	303.4	1367.9	2643.4	1120.9	328.4	144.2	144.1	144.1	564.4
87	Neman	4559.5	4559.5	4559.5	4880.6	8336.2	8420.3	8262.7	11094.1	7335.1	4682.7	4559.5	4559.5	6317.4
88	Fraser	8611.9	8611.9	8617.0	9741.4	11359.1	12187.4	15646.7	18285.4	12788.9	9019.3	8619.4	8611.9	11008.4
89	Severn(Trib. Hudson Bay)	45.4	45.4	45.4	45.4	45.4	45.4	45.4	45.4	45.4	45.4	45.4	45.4	45.4
90	Amur	61291	61363	69115	435992	1515404	2321588	1258873	758246	521703	92588	69587	64788	602545
91	Tweed	326.3	326.3	326.3	326.3	326.6	333.2	395.3	404.4	368.3	326.9	326.3	326.3	342.7
92	Grande Riviere De La Balei	3.7	3.7	3.7	3.7	3.7	3.7	3.7	3.7	3.7	3.7	3.7	3.7	3.7
93	Grande Riviere	10.0	10.0	10.0	10.0	10.0	10.0	10.0	10.0	10.0	10.0	10.0	10.0	10.0
94	Winisk	40.6	40.6	40.6	40.6	40.6	40.6	40.6	40.6	40.6	40.6	40.6	40.6	40.6
95	Churchill, Fleuve (Labrador)	57.7	57.7	57.7	57.7	57.7	57.7	57.7	57.7	57.7	57.7	57.7	57.7	57.7
96	Dniepr	58219.8	58219.8	58220.8	75741.6	230947.0	285228.0	363212.3	338828.4	166626.4	71978.5	58731.2	58219.8	152014.4
97	Ural	7719.8	7719.8	7726.0	29996.9	138276.3	227767.1	379764.5	304376.4	113415.5	34294.8	9006.6	7722.5	105648.9
98	Wisla	42823.6	42823.6	42830.6	43383.9	50173.1	53458.7	53639.2	64292.7	55353.7	44967.6	42827.3	42823.6	48283.1
99	Don	39722.6	39722.6	39722.7	104613.8	508233.8	647321.8	790482.4	672631.7	249166.3	77697.2	40938.9	39722.6	270831.3

Basin ID	Basin name	Blue water footprint (10^3 m^3/month)												
		Jan	Feb	Mar	Apr	May	Jun	Jul	Aug	Sep	Oct	Nov	Dec	Average
100	Oder	29979.2	29979.2	29987.5	30402.4	34120.9	37229.9	40736.3	46184.5	40612.3	31840.4	29986.3	29979.2	34253.2
101	Elbe	44757.5	44757.5	44796.9	45993.8	47830.3	52688.8	71886.0	85614.6	76979.8	50498.2	44792.1	44757.7	54612.8
102	Trent	3851.8	3851.8	3856.6	3867.3	4113.6	4724.6	7918.2	7500.9	5254.9	3919.6	3851.8	3851.8	4713.6
103	Weser	18785.6	18785.6	18786.8	18885.3	19314.9	21212.5	29191.0	36488.8	30603.1	19801.2	18785.8	18785.6	22452.2
104	Attawapiskat	9.5	9.5	9.5	9.5	9.5	9.5	9.5	9.5	9.5	9.5	9.5	9.5	9.5
105	Eastmain	2.9	2.9	2.9	2.9	2.9	2.9	2.9	2.9	2.9	2.9	2.9	2.9	2.9
106	Manicouagan (Riviere)	92.6	92.6	92.6	92.6	92.6	92.8	92.8	92.9	92.7	92.6	92.6	92.6	92.7
107	Columbia	34262	35262	180824	848539	1447369	2311177	3409891	2913847	1540886	615283	129775	41987	1125758
108	Little Mecatina	1.0	1.0	1.0	1.0	1.0	1.0	1.0	1.0	1.0	1.0	1.0	1.0	1.0
109	Natashquan (Riviere)	3.4	3.4	3.4	3.4	3.4	3.4	3.4	3.4	3.4	3.4	3.4	3.4	3.4
110	Rhine	122345.5	122345.5	122352.6	123279.3	135280.2	140236.7	145768.4	176128.5	150043.6	124553.1	122345.5	122345.5	133918.7
111	Albany	128.2	128.2	128.2	128.2	128.3	128.6	128.9	128.8	128.4	128.2	128.2	128.2	128.4
112	Saguenay (Riviere)	2088.6	2088.6	2088.6	2088.6	2102.2	2206.7	2155.3	2134.6	2095.1	2088.6	2088.6	2088.6	2109.5
113	Thames	7697.0	7697.0	7697.1	7699.1	7726.2	7880.7	8220.7	8141.2	7885.4	7709.7	7697.0	7697.0	7812.4
114	Nottaway	293.0	293.0	293.0	293.0	293.2	293.3	293.3	293.1	293.0	293.0	293.0	293.0	293.1
115	Rupert	2.7	2.7	2.7	2.7	2.7	2.7	2.7	2.7	2.7	2.7	2.7	2.7	2.7
116	Moose(Trib. Hudson Bay)	815.6	815.6	815.6	815.7	821.0	824.1	827.2	823.3	816.0	815.6	815.6	815.6	818.4
117	St.Lawrence	383010.0	383010.0	383187.1	386034.2	408783.9	451638.1	537777.5	564415.6	478676.0	402709.1	383289.8	383022.3	428796.1
118	Danube	172885.0	172888.3	176401.4	214410.4	349900.8	428431.0	640658.5	692509.3	429867.8	245115.7	174598.8	172895.0	322546.8
119	Seine	46280.8	46280.8	46499.4	48950.3	59473.4	72701.6	118179.0	156201.5	116779.5	56640.6	46296.5	46280.8	71713.7
120	Dniestr	13797.1	13797.1	13851.0	21101.3	69002.6	80201.6	60248.9	113028.4	63236.6	20506.5	13898.7	13797.1	41372.2
121	Southern Bug	5970.1	5970.1	5970.1	8880.2	28585.4	34379.8	50055.3	50401.7	21160.3	8384.4	6097.0	5970.1	19318.7
122	Mississippi	476071.5	553677.1	1066448	1676456	2574769	3671826	9923789	12809395	8325019	2696248	679909	513391	3747250
123	Skagit	428.9	428.9	428.9	429.4	436.7	908.0	1462.7	1546.0	820.1	431.6	428.9	428.9	681.6
124	Aral Drainage	51679.1	48145.8	215735.5	1182471	2320721	4541763	8587253	8909592	6123848	2291408	281293	100329	2887853
125	Loire	23162.6	23162.6	23736.9	26573.5	39703.4	65121.6	165091.3	251733.7	171018.7	48758.7	23288.3	23162.6	73709.5
126	Rhone	28384.8	28529.3	29642.5	32065.3	41461.0	58054.0	141031.2	150460.5	72119.4	32102.8	28758.1	28384.9	55916.2
127	Saint John	2582.5	2582.5	2582.5	2582.5	2587.8	2736.4	3219.2	4622.8	2960.2	2594.6	2582.5	2582.5	2851.3
128	Po	40929.7	40933.9	41954.4	44063.6	126507.8	202893.7	617810.5	620682.7	211145.4	53621.6	40929.9	40929.7	173533.6
129	Penobscot	765.3	765.3	765.3	765.5	767.3	777.6	865.5	1048.9	825.9	770.4	765.3	765.3	804.0
130	St.Croix	120.1	120.1	120.1	120.1	120.2	124.5	129.3	135.6	124.2	120.1	120.1	120.1	122.9
131	Kuban	6573.6	6573.6	6573.6	10296.5	77019.1	160897.1	291432.5	165757.2	37165.1	9876.5	6599.1	6573.6	65444.8
132	Connecticut	10498.8	10498.8	10499.1	10554.3	10865.5	12300.3	12701.8	10951.6	10645.2	10524.8	10506.4	10498.8	10920.4
133	Liao He	25918.6	27536.5	43955.3	421314	1382065	1906167	1116163	667477	467166	49664.8	33284.3	30489.8	514266.7
134	Garonne	9783.0	9804.2	11113.9	13422.2	20437.9	38994.0	217419.2	288619.9	187398.5	45387.3	11066.3	9783.0	71935.8
135	Ishikari	3230.4	3230.4	3230.4	3254.8	3378.9	14603.7	15213.4	19830.1	11559.0	3991.3	3230.4	3230.4	7331.9
136	Merrimack	11384.4	11384.4	11384.5	11418.1	11515.8	11710.1	11758.7	11498.9	11424.6	11408.5	11387.6	11384.5	11471.7
137	Hudson	19701.2	19701.2	19702.3	19776.2	19909.2	20191.0	21219.1	21213.9	20235.2	19767.0	19718.1	19701.3	20069.6
138	Colorado(Pacific Ocean)	51531.4	79016.8	258871.8	465243.9	688780.7	833506.3	868950.9	785259.8	598564.3	367116.3	152984.9	88178.4	436500.5
139	Klamath	695.1	695.1	875.6	28761.1	81554.8	127597.5	176238.8	151941.9	92866.9	28794.6	2489.8	695.1	57767.2
140	Ebro	4822.5	10975.0	46643.5	78434.8	122848.5	275459.1	587776.7	525750.6	242242.7	68777.9	11223.1	5629.7	165048.7
141	Rogue	1317.7	1317.7	1366.0	4252.0	11582.2	20252.7	27198.1	23091.3	14726.5	4929.1	1336.1	1317.7	9390.6
142	Douro	5884.1	7786.1	20223.4	41082.9	74657.3	252660.6	601045.1	614466.1	242744.1	45678.2	7325.7	5886.8	159953.4
143	Susquehanna	20293.7	20293.8	20304.9	20419.3	20885.0	21594.8	24593.3	26111.7	22846.0	20997.1	20312.5	20294.9	21578.9
144	Luan He	14156.1	63826.7	198095.8	369022.7	439376.3	226806.4	192323.3	207433.2	160173.2	62890.7	14435.4	11342.7	163323.5
145	Kura	26370.9	30851.7	107105.3	282772.4	308423.7	521039.7	733223.8	807810.5	455357.5	167331.3	53554.5	38164.1	294333.8
146	Dalinghe	3816.9	4321.7	6670.5	24989.9	66489.8	97171.0	50590.6	36848.1	27222.0	6012.0	4571.4	4326.1	27752.5
147	Delaware	32242.6	32244.3	32284.9	32560.0	34246.2	37078.2	37708.9	34841.7	33292.8	32505.0	32316.5	32254.2	33631.3
148	Sacramento	15241.3	15248.2	48730.6	287969	667890	1235885	1591869	1566041	1081215	300097	40641	15584	572201
149	Huang He (Yellow River)	217673	738449	2375921	4267862	4256628	3400184	3466422	2159613	992897	434435	188078	176407	1889547
150	Kizilirmak	4234.6	4254.3	7119.9	39274	119425	168870	187790	206397	129236	49110	14939	5399	78004
151	Yongding He	99988.0	545652.6	1990251	3417354	3359369	1712020	1842686	1930023	1020618	352702	102495	96961	1372510
152	Tejo	11231.7	14362.0	30236.9	47754.6	82013.5	223938.2	441127.2	433196.6	200765.3	50828.6	14147.8	11245.0	130070.6
153	Sakarya	5368.1	5386.4	8192.8	31515.7	95507.6	139487.4	174554.7	209581.8	140841.5	50135.6	9821.4	5927.5	73026.7
154	Eel (Calif.)	188.2	188.2	188.5	235.2	657.9	1102.8	1441.8	1205.3	846.8	282.0	190.2	188.2	559.6
155	Tigris & Euphrates	205397.3	718731.7	2729822	5090895	6654136	4850558	4639864	4544688	2850413	1543961	664503	264649	2896468
156	Potomac	17093.4	17093.9	17117.0	17504.4	18009.5	18871.4	19799.9	20398.5	18608.5	17477.1	17133.5	17096.7	18017.0
157	Guadiana	2588.8	11643.9	52785.6	92804.4	158832.5	420029.3	737694.5	702725.4	330357.8	95626.3	12663.1	3155.2	218408.9
158	Kitakami	2130.2	2130.8	2134.3	2137.7	2162.4	7457.6	19066.8	45846.8	28745.3	3920.7	2130.2	2131.7	9999.5
159	Mogami	1860.1	1860.1	1861.2	1880.2	1927.3	6926.2	10796.2	31753.9	14549.3	3721.7	1860.1	1860.1	6738.0
160	Han-Gang (Han River)	16927.3	16934.6	16961.0	17284.7	21933.1	37684.3	27829.3	22227.9	27161.2	17042.1	16943.4	16938.6	21322.3
161	Guadalquivir	6527.2	33894.1	123992.3	189770.9	279532.8	689193	1097659	1047458	503164	161945.8	34484.8	10685.8	348192.2
162	San Joaquin	8455.2	9670.6	92792.8	399075.9	658744.8	1062562	1459542	1459787	1013340	379470.4	66973.1	13882.9	552024.8
163	James	4539.4	4540.4	4547.5	4915.6	5152.6	5472.9	6092.1	6322.3	5119.3	4966.6	4569.3	4540.0	5064.8
164	Bravo	52585.2	100575.1	248393.7	392946.1	525645.7	497835.0	599657.0	567057.2	464507.0	286434.9	105140.1	72297.3	326089.5
165	Shinano, Chikuma	3548.2	3548.2	3550.4	3587.8	3678.9	6456.5	16274.8	37548.0	13720.9	4472.4	3548.3	3548.5	8623.6
166	Roanoke	7459.3	7460.9	7501.9	9261.5	11279.4	12219.9	14283.4	15908.4	11010.0	9934.4	7680.5	7460.6	10121.7
167	Naktong	11953.1	12079.3	12273.1	12489.7	20491.6	78141.3	30160.6	55497.2	57360.2	12754.4	12180.0	12134.1	29643.6
168	Indus	6455179	7692491	14959408	13807935	6182331	6262009	8796342	13190821	16068994	13120835	7128949	3924286	9799132
169	Tone	16652.4	16658.4	16684.8	16921.1	17434.2	30525.3	54229.0	105969.7	50170.4	20794.1	16652.7	16658.4	31612.5
170	Salinas	1560.7	1560.7	1714.9	8299.6	24644.6	52141.5	82755.9	87842.6	55421.4	11439.2	2716.4	1588.6	27640.5
171	Pee Dee	13141.8	13137.8	13237.6	14886.2	17965.8	19219.3	20735.8	21667.2	17175.0	14965.9	13358.8	13144.5	16053.0
172	Chelif	2243.7	4486.6	11906.5	19297.1	32072.6	55384.2	71153.4	66882.8	45925.4	14779.6	5989.0	3961.0	27840.2
173	Cape Fear	8243.5	8240.8	8485.1	10325.2	15144.1	15161.8	13943.7	14504.2	11093.5	9492.9	8437.9	8244.8	10943.1
174	Tenryu	2246.4	2246.4	2246.6	2248.0	2257.8	3038.9	4244.1	9498.5	4323.8	2375.5	2247.5	2246.6	3268.3
175	Santee	15848.2	15848.7	15928.6	16585.8	17815.1	18043.5	19724.0	19815.2	17430.5	16623.7	16044.5	15861.2	17130.7
176	Kiso	3159.0	3159.2	3159.2	3159.5	3160.5	3417.9	4734.5	8819.0	4727.2	3469.9	3159.7	3159.4	3940.4
177	Yangtze(Chang Jiang)	450511.6	711676.0	1138406	2002161	3060663	2339674	3741942	3868817	3424557	511004	356944	371101	1831455
178	Yodo	16043.7	16046.0	16047.9	16128.7	16362.8	21036.0	36931.7	71186.4	31780.5	21024.7	16047.3	16048.2	24557.0
179	Sebou	3773.3	21476.2	78740.1	187653.4	202084.4	168880.9	204203.9	162631.6	125903.4	63496.1	21559.7	5086.2	103790.8
180	Alabama River & Tombigbee	21968.7	21970.9	21985.2	22295.5	23759.7	24523.0	28772.0	31987.1	26194.1	23480.1	22074.9	21970.5	24248.5
181	Savannah	5927.7	5936.8	6054.9	6580.1	7448.3	8419.0	10987.8	13392.7	8444.6	7316.6	6083.3	5962.8	7712.9
182	Gono (Go)	667.8	668.1	669.3	673.9	696.4	1130.1	1457.8	3598.7	1559.7	1162.5	668.4	668.7	1135.1
183	Huai He	84982.9	147657.0	567969.3	1635316	1948176	1581174	1624787	1330904	1160434	234794.7	92572.2	90858.3	874968.8
184	Apalachicola	15024.4	15066.2	15382.3	19427.6	28387.7	44625.3	75496.9	125950.5	56360.2	39981.7	16002.7	15121.5	38902.3
185	Brazos	29558.9	48605.7	117767.4	168556.2	282486.0	395665	1060283	973829.0	599180.5	189533.7	37740.8	24382.9	327299.1
186	Altamaha	12223.9	12281.3	12675.9	14587.9	17877.9	21013.5	36291.0	45297.4	26091.4	19718.3	12513.6	12265.8	20236.5
187	Mekong	757008.7	440654.9	582312.0	754421.9	1240389	805570.7	659922.9	528371.9	283186.5	739371.1	1180498	767382.8	728257.6
188	Colorado(Caribbean Sea)	15522.5	23764.4	55151.7	79227.2	133806.1	214639.8	522178.2	524039.6	371594.0	123331.4	19980.8	14106.5	174778.5
189	Trinity(Texas)	27495.9	27688.4	29144.4	32670.3	34251.9	36749.3	45035.5	41220.4	33270.3	29203.9	27948.6	27522.9	32683.5
190	Pearl	3156.3	3156.5	3157.7	3191.7	3317.1	3336.0	3450.4	3628.9	3334.6	3202.7	3160.4	3156.4	3270.7
191	Sabine	2914.3	2943.5	3248.5	4993.8	7985.4	8449.1	12327.0	8889.5	4780.5	3403.6	3076.1	2919.7	5494.3
192	Suwannee	3006.2	3067.6	3435.9	6565.9	14272.2	16998.2	29457.6	42620.1	19697.9	12591.8	3713.5	3045.1	13206.0
193	Yaqui	12804.7	40790.9	85472.2	98556.4	48621.7	32724.7	24545.0	38239.4	44900.4	29924.7	14373.1	12757.3	40309.2
194	Nile	1596883	1639017	2685612	2819450	3108601	2101369	2924081	3198978	3529943	3321549	2035678	1000401	2496797
195	Brahmaputra	95618.5	76934.9	132628.5	102108.9	68629.6	35127.7	78327.4	55174.6	94765.4	478232.7	455027.2	88132.9	146725.7
196	St.Johns	14741.8	16036.7	17961.2	18742.7	23549.9	19402.7	17594.6	18178.2	15870.8	15861.3	15025.0	14786.2	17312.6
197	Nueces	5736.2	11373.9	28924.4	38248.5	48415.9	56133.5	89955.4	66684.0	33032.0	14181.0	7292.2	5200.8	33764.8
198	San Antonio	4887.5	6350.4	12503.9	16007.5	17404.6	19929.4	30160.6	22397.7	11958.6	6937.6	5209.5	4789.1	13211.4
199	Irrawaddy	61867.7	62279.7	109496.0	150159.0	107761.4	338248.0	133305.1	88392.4	241220.5	554244.8	147710.9	40590.2	169606.3
200	Fuerte	2992.4	7032.5	14502.1	27035.1	31771.5	43066.3	41879.3	44576.8	23372.6	28061.2	7859.1	6195.4	23195.4
201	Xi Jiang	112099.1	148679.4	188596.5	394758.4	535641.1	313893.2	296653.6	302293.3	534178.9	83317.1	67032.5	78758.5	254658.5
202	Bei Jiang	17753.0	17808.0	17866.4	19713.7	35915.3	40428.0	79708.7	61083.2	75276.0	20831.8	19111.1	18475.3	35330.9

Basin ID	Basin name	Blue water footprint (10^3 m^3/month)												
		Jan	Feb	Mar	Apr	May	Jun	Jul	Aug	Sep	Oct	Nov	Dec	Average
203	San Pedro	1970.2	3084.5	5653.0	8269.6	9543.3	4601.2	3673.3	12070.8	22712.6	15185.2	3834.6	3921.9	7876.7
204	Dong Jiang	11698.5	11620.1	11600.6	13139.6	24274.1	25818.1	50233.2	35538.3	41690.7	12980.1	12451.0	12101.6	21928.8
205	Mahi	234803.3	213391.3	332457.9	301590.9	185769.5	51117.1	24897.0	40458.9	80569.1	151801.5	127823.0	145556.7	157519.7
206	Damodar	321001.6	150895.6	217247.6	60473.5	20235.7	18013.7	57510.2	38370.4	50884.2	128180.7	281488.4	245126.1	132452.3
207	Niger	117175.3	133775.1	159994.2	102340.4	247266.2	227449.8	190715.7	142446.1	193011.8	207068.0	64979.2	72785.9	154917.3
208	Narmada	591222	582117	1216831	1529310	1537921	408983.0	38561.8	50038.6	112396.8	249632.9	212263.3	398514.2	577316.0
209	Brahmani River (Bhahmani)	108131.9	45430.0	71957.7	57563.0	52284.8	23326.4	23378.5	18202.0	31067.4	69816.1	110970.6	105337.9	59788.9
210	Mahanadi(Mahahadi)	493904.3	146732.8	213472.7	204175.8	213763.2	76145.2	93534.4	66762.9	209738.6	494316.1	598494.6	475411.3	273871.0
211	Santiago	69433.9	156850.1	329195.1	358517.6	227133.2	88512.1	63621.5	80404.7	178113.9	230843.2	139603.3	107577.7	169150.5
212	Panuco	59147.5	128380.2	251479.9	266556.7	186223.8	80759.8	60191.8	75690.8	96250.7	104272.6	69303.7	80568.1	121568.8
213	Godavari	1403293	846541	1675666	1996301	2123564	823832	551196	539414	663656	1168966	1408048	1423182	1218638
214	Tapti	276668.3	227566.1	414078.5	494077.2	533823.8	201524.6	90419.5	118814.1	198954.2	310907.4	278697.0	277175.7	285225.5
215	Sittang	3465.5	4516.6	8520.0	8858.3	5784.9	35071.4	17130.2	7752.7	31031.5	44533.4	9145.4	2562.4	14864.4
216	Armeria	3496.4	10961.6	23001.5	38879.1	37308.7	13663.1	4419.5	2587.5	2146.6	13316.5	11430.0	13509.5	14560.0
217	Ca	10049.8	9707.5	7879.1	11152.4	40910.6	18897.3	12304.8	4239.3	4066.4	4907.1	5918.1	6333.7	11363.8
218	Chao Phraya	500052	429799	702371	726552	447428	309828	1301998	1314239	977560	1175695	2152860	752664	899254
219	Krishna	2085475	825245	1696212	1831925	1892192	1086965	1387624	1679785	2348322	1758006	2284196	2233875	1759152
220	Senegal	26195.1	13556.4	20473.6	15815.1	16250.3	15939.4	41069.3	35983.9	35292.4	59240.1	51877.4	28735.1	30035.7
221	Papaloapan	5755.1	10892.1	18931.4	19716.5	14544.8	7490.8	7494.8	11051.7	6995.3	12754.2	9045.0	9997.6	11222.4
222	Grisalva	9068.6	12611.1	38902.8	60109.3	42527.8	15144.1	9803.9	13796.8	8004.6	8094.8	14728.7	24789.5	21465.2
223	Verde	1688.1	3855.9	8808.5	9301.3	6019.0	2591.5	2556.6	2616.5	3248.5	2719.8	3776.9	4438.0	4301.7
224	Mae Klong	25670.1	28215.2	46157.7	45329.9	20724.5	11506.4	52439.2	66338.2	44699.2	24524.1	55270.0	32920.0	37816.2
225	Tranh (Nr Thu Bon)	4406.8	4982.2	3362.2	4165.1	26187.3	28753.8	26879.3	12805.6	1545.2	1535.0	1631.9	2428.4	9890.3
226	Penner	183705.0	46423.6	70472.3	60887.3	59123.8	56079.9	214195.2	204265.8	247099.0	191439.6	245016.4	187965.9	147222.8
227	Volta	9567.8	10442.0	12602.1	8230.3	5987.6	8736.4	7358.4	5708.3	6382.8	10244.7	7843.7	7902.8	8417.3
228	Lempa	8417.2	3985.4	10300.0	13889.0	6041.7	3074.9	2889.5	2900.3	2579.5	3108.5	6446.6	9496.9	6094.1
229	Gambia	331.1	367.9	421.2	366.2	382.3	290.1	1097.0	855.3	818.6	1268.8	342.0	338.4	573.2
230	Grande De Matagalpa	566.0	594.8	3029.8	4736.1	1566.4	402.8	637.9	1219.1	440.0	276.3	199.7	523.6	1182.7
231	Cauvery	458718	207070	515498	458868	442335	449788	1508522	1507427	1445163	774266	560226.2	465899.2	732815.0
232	San Juan	9649.7	9142.5	18721.9	27753.9	9814.9	4224.3	5693.1	9177.3	6153.5	3690.4	3351.3	6276.3	9470.8
233	Geba	3460.3	4754.7	5841.6	5808.9	4717.1	1657.3	372.7	80.4	279.3	318.8	2963.3	4028.0	2856.9
234	Corubal	386.5	521.0	617.3	612.7	529.8	236.9	108.2	90.1	111.3	141.8	329.7	445.6	344.2
235	Magdalena	36962.3	40595.1	109023.0	121897.9	126453.2	143794.5	277591.3	321763.7	124292.8	46851.9	36228.9	37982.4	118619.8
236	Comoe	3723.0	4563.1	6155.5	3805.2	2893.7	2868.3	2656.4	2317.6	2150.1	4090.6	4433.9	5521.1	3764.9
237	Orinoco	51166.0	75192.9	148018.5	116530.6	58659.7	56289.8	84861.4	118091.2	86830.9	29194.6	26893.7	62694.2	76202.0
238	Bandama	3618.5	5359.1	7722.2	6704.9	5053.7	1699.8	1779.8	1487.8	1212.6	2538.2	5959.7	8605.1	4311.8
239	Oueme	965.2	1307.5	1498.3	1155.2	861.4	648.6	653.4	607.9	634.5	587.3	956.1	1201.2	923.1
240	Sassandra	1176.2	1950.1	4275.9	3602.8	2184.4	742.6	559.7	581.4	547.9	844.0	2016.3	3249.2	1810.9
241	Shebelle	78624.7	60600.1	38694.8	18624.5	18867.7	141778.8	217842.5	123581.2	47896.3	37851.8	28963.6	48183.1	71792.4
242	Mono	400.3	450.1	431.1	311.4	284.5	257.8	253.3	276.6	256.5	244.1	277.6	336.7	315.0
243	Congo	7425.3	9630.1	9895.9	9371.1	19489.5	31988.4	33458.0	35361.3	32674.7	24805.9	8041.2	5782.6	18993.7
244	Atrato	596.3	596.9	620.1	682.7	619.3	595.2	595.6	597.3	595.9	595.0	595.0	595.2	607.1
245	Cuyuni	188.7	205.4	258.7	243.8	192.8	173.8	173.5	201.6	217.8	210.9	201.0	199.8	205.6
246	Cavally	224.8	264.5	339.2	278.6	162.7	136.0	154.8	174.3	168.0	190.3	225.8	270.4	215.8
247	Tano	207.1	226.5	206.9	166.7	153.9	149.5	155.2	169.7	189.7	158.2	161.4	201.3	178.8
248	Cross	1586.0	1864.2	1722.9	1362.9	1270.9	1236.3	1224.4	1224.2	1222.4	1226.9	1382.7	1766.8	1424.2
249	Sanaga	1625.2	2104.0	1347.5	1037.7	567.7	531.5	466.5	444.4	446.7	507.6	1440.6	2049.2	1047.4
250	Pra	4539.5	4288.5	3579.0	1338.1	879.6	617.9	973.8	1169.3	854.9	487.4	486.7	2106.0	1776.7
251	Davo	176.9	219.2	214.8	197.5	105.9	84.7	106.8	161.7	147.3	142.5	147.0	237.7	161.8
252	Essequibo	20.8	20.8	20.8	20.8	20.8	20.8	20.8	20.8	20.8	20.8	20.8	20.8	20.8
253	Kelantan	39173.4	29665.9	1797.7	1179.2	1582.4	3175.2	4628.0	5039.6	30769.7	16146.9	8351.1	4871.7	12198.4
254	Corantijn	53.6	53.6	53.6	53.6	73.0	58.8	156.3	249.1	923.1	548.6	193.2	68.3	207.1
255	Coppename	10.1	10.1	10.1	10.1	10.1	10.1	10.1	10.1	10.1	10.1	10.1	10.1	10.1
256	Kinabatangan	235.4	201.9	201.9	205.0	211.2	210.0	209.7	255.4	322.7	253.0	231.7	234.9	231.1
257	Maroni	8.8	8.8	8.8	8.8	8.8	8.8	8.8	8.8	8.8	8.8	8.8	8.8	8.8
258	San Juan (Columbia - Pacifi	990.4	1280.5	2848.2	2452.2	2799.0	3381.4	6309.4	6367.6	2508.4	1066.0	871.8	995.3	2655.8
259	Amazonas	90048.9	82160.0	98044.1	250757.2	285935.4	217698.9	191979.8	286579.2	288724.7	205464.6	148718.1	82683.1	185732.8
260	Pahang	21420.6	9762.1	1971.7	2172.3	2408.5	4547.0	6821.8	6363.9	19897.8	10406.4	9369.4	6314.9	8454.7
261	Nyong	125.9	125.8	125.5	125.3	125.0	125.0	125.1	125.1	125.0	125.0	125.0	125.4	125.3
262	Oyapock	6.2	6.2	6.2	6.2	6.2	6.2	6.2	6.2	6.2	6.2	6.2	6.2	6.2
263	Rajang	1381.3	401.3	276.8	275.4	281.3	277.3	297.9	297.1	642.4	454.9	647.3	1066.2	524.9
264	Ntem	182.2	183.4	180.7	179.4	178.9	178.9	178.9	178.9	178.9	178.9	178.9	180.3	179.9
265	Ogooue	600.6	546.4	478.0	302.8	265.3	369.3	713.9	1155.4	842.7	478.2	246.6	337.6	528.1
266	Rio Araguari	24.8	24.8	24.8	24.8	24.8	24.8	24.8	24.8	24.8	24.8	24.8	24.8	24.8
267	Mira	5669.4	4594.5	3362.4	2750.4	3808.6	5730.0	12593.3	24869.5	19047.6	6104.3	5516.5	3371.0	8118.1
268	Esmeraldas	14478.9	11223.3	8395.3	7606.1	8790.7	11571.1	27996.9	53475.8	38916.5	17927.0	24302.0	13038.1	19810.1
269	Tana	11163.8	11021.9	3741.6	1002.6	920.8	2771.7	6243.1	8141.2	7988.8	4856.0	1270.2	4218.5	5278.3
270	Daule & Vinces	76294.6	27282.6	15585.9	10387.3	21676.0	48581.3	111075.1	180108.2	150194.0	76443.7	131809.0	71459.2	76741.4
271	Rio Gurupi	199.6	185.4	185.1	185.5	192.2	228.4	253.2	267.7	261.0	235.6	215.4	200.1	217.4
272	Rio Capim	483.6	471.3	471.3	471.0	473.7	480.5	487.3	498.7	524.3	509.9	510.5	486.3	489.1
273	Tocantins	17599.9	11559.6	10800.6	20018.0	13298.0	17296.2	21141.7	23947.2	20498.2	11143.5	10846.6	11621.8	15814.3
274	Kouilou	80.2	80.6	78.7	78.8	328.9	2036.2	2923.4	3622.1	3720.0	2881.4	637.8	78.6	1378.9
275	Nyanga	12.5	12.5	12.5	12.5	12.5	12.5	12.5	12.5	12.5	12.5	12.5	12.5	12.5
276	Rio Parnaiba	7330.6	5752.8	4658.7	6687.2	13878.3	19976.6	23026.0	25728.3	24345.8	20674.2	13068.3	11625.1	14729.3
277	Rio Itapecuru	1038.7	919.8	862.8	976.1	1843.3	2457.8	2708.8	2785.4	2449.8	1974.0	1276.0	1055.5	1695.7
278	Rio Acarau	706.2	808.0	582.4	481.1	1001.3	2657.1	3081.1	3854.0	4372.7	4049.2	3535.8	2922.3	2337.6
279	Pangani	6122.5	21481.4	20589.8	13511.5	23766.6	57621.2	68179.9	35271.8	31027.4	22244.9	7098.8	5938.5	26071.2
280	Rio Pindare	469.5	433.7	429.4	436.5	460.4	528.3	560.4	575.3	571.6	540.4	486.2	474.7	497.2
281	Sepik	69.0	69.0	69.0	69.0	69.0	69.0	69.0	69.0	69.0	69.0	69.0	69.0	69.0
282	Rio Mearim	1054.4	880.1	825.0	951.2	1188.8	1609.1	1764.5	1825.4	1771.5	1592.6	1140.1	1074.0	1306.4
283	Chira	26561.8	14163.4	4811.4	5996.6	11137.9	11924.2	20562.3	31761.0	27866.3	15536.5	22752.6	17981.7	17588.0
284	Rufiji	8962.8	8069.6	6182.5	14982.0	35507.8	19053.8	13350.6	12116.6	11378.6	9694.2	4804.0	5735.0	12486.4
285	Rio Jaguaribe	8821.3	10924.3	7871.0	9318.9	24950.8	35430.5	39452.8	48828.2	56859.5	52105.1	40421.8	30580.7	30463.7
286	Purari	66.8	66.8	66.8	66.8	66.8	66.8	66.8	66.8	66.8	66.8	66.8	66.8	66.8
287	Ruvu	3703.6	1994.0	1510.1	804.6	2727.2	2346.2	2409.8	2873.0	2727.9	2245.8	1384.8	1773.4	2208.4
288	Rio Paraiba	2470.4	2697.3	2946.2	2212.0	2943.5	2971.3	4130.0	5962.4	11502.7	11706.3	10296.8	8106.6	5662.1
289	Solo (Bengawan Solo)	333523.1	96711.1	38593.2	3097.7	4184.1	15196.8	30962.6	48138.2	78677.7	51440.1	274543.7	172560.2	95635.7
290	Sao Francisco	27576.3	37265.5	57937.4	94628.2	121453.3	122548.9	132061.6	169133.9	177712.7	120696.3	58297.6	40099.8	96617.6
291	Brantas	180435.4	52134.7	19233.3	2510.4	2889.4	8721.2	20930.1	35078.7	52881.4	28404.6	169820.2	102706.1	56312.1
292	Santa	4443.4	4714.9	5859.8	17926.2	18412.2	16769.7	13970.0	21548.3	24273.3	12229.0	8988.3	3339.1	12706.2
293	Zambezi	18945.8	18076.7	31935.5	85399.4	117638.8	124036.6	148703.9	214596.6	260861.2	218911.7	101555.8	33946.1	114550.7
294	Rio Vaza-Barris	1612.9	1939.0	2318.3	2205.0	1895.9	1614.1	1815.3	2315.2	3117.0	2951.9	2081.0	1854.0	2143.3
295	Rio Itapicuru	2629.4	3113.9	2876.2	3181.5	3252.1	2851.4	3307.0	4175.5	5578.0	5672.9	4047.1	3619.3	3692.0
296	Rio Paraguacu	4204.4	5205.6	6584.1	12009.8	15360.5	12145.9	12096.3	17812.0	20447.7	16656.5	8324.7	6676.8	11460.4
297	Canete	2538.0	1777.9	3242.3	6624.0	7540.1	5536.9	3993.8	5323.5	5539.0	5606.5	4349.0	2983.8	4587.9
298	Rio De Contas	5876.9	6331.4	10008.6	16689.7	22514.9	18702.6	23056.0	33644.2	36795.0	25500.9	9867.6	7560.9	18045.7
299	Roper	10.1	8.5	107.9	661.6	999.3	939.6	997.4	1160.4	1255.2	1106.4	431.3	75.4	646.1
300	Daly	32.7	30.4	148.0	896.6	1355.4	1322.9	1392.6	1547.8	1659.7	1372.8	422.2	77.1	854.8
301	Drysdale	4.4	4.4	4.4	4.4	4.4	4.4	4.4	4.4	4.4	4.4	4.4	4.4	4.4
302	Parana	372525.6	280676.9	232433.0	310336.0	224416.6	282362.5	389987.9	484246.3	441763.5	351021.8	281804.3	210576.8	321845.9
303	Durack	4.5	4.5	4.5	4.5	4.5	4.5	4.5	4.5	4.5	4.5	4.5	4.5	4.5
304	Rio Prado	845.8	1144.8	1246.2	1744.2	2161.6	1830.6	2190.6	3280.5	3413.9	2676.9	1327.9	968.0	1902.6
305	Victoria	2.8	2.8	2.8	2.8	2.8	2.8	2.8	2.8	2.8	2.8	2.8	2.8	2.8

Basin ID	Basin name	Blue water footprint (10^3 m^3/month)												
		Jan	Feb	Mar	Apr	May	Jun	Jul	Aug	Sep	Oct	Nov	Dec	Average
306	Mitchell(N. Au)	341.7	261.0	734.6	3963.8	5537.1	5395.0	5844.0	6913.5	8084.4	7994.6	4814.7	1996.1	4323.4
307	Majes	3314.9	2330.3	2230.2	6243.8	6557.9	3327.0	2217.4	3421.6	4721.7	4279.7	3531.6	2713.2	3740.8
308	Ord	10.2	5.1	95.9	2642.1	5015.6	6620.6	8488.8	9999.0	10849.1	8894.4	4903.3	290.0	4817.8
309	Jequitinhonha	945.8	1485.9	1556.0	2000.2	2114.7	2118.7	2390.9	2950.1	2962.3	2164.1	1094.5	855.1	1886.5
310	Macarthur	1.1	1.1	1.1	1.1	1.1	1.1	1.1	1.1	1.1	1.1	1.1	1.1	1.1
311	Fitzroy	11.9	12.0	12.4	14.1	14.2	13.9	14.3	14.9	15.2	15.3	14.4	12.5	13.8
312	Gilbert	7.4	7.0	69.2	192.1	192.0	166.5	177.4	213.7	246.0	244.9	166.5	86.0	147.4
313	Mucuri	398.8	697.6	722.2	893.4	1042.3	1273.0	1400.9	1994.9	1783.8	1336.8	535.7	375.9	1037.9
314	Rio Doce	4008.0	11396.5	10312.4	13025.3	15031.5	19754.2	25232.1	29963.7	24373.8	18958.1	6088.4	3693.6	15153.1
315	Save	7032.9	7311.8	12439.9	22876.0	18101.7	21476.8	25475.9	52038.5	66437.5	48232.1	15712.5	7525.0	25388.4
316	Burdekin	1069.8	1069.6	6076.7	16014.8	13433.7	13045.9	15661.6	19538.3	24172.3	24665.8	17069.6	5682.5	13125.1
317	Tsiribihina	60722.5	42436.5	88041.6	170196.4	57454.6	3648.5	3562.4	3689.5	3846.5	3978.5	2776.9	19607.2	38330.1
318	Buzi	339.3	168.7	571.0	1950.0	2369.0	2285.2	2411.6	3704.3	4836.8	4651.0	1881.5	694.8	2155.3
319	Loa	348.3	348.2	349.1	344.5	338.5	344.2	350.9	378.1	394.9	396.1	358.0	357.0	359.0
320	Limpopo	98842.0	124195.8	219628.8	213847.8	120149.9	125648.9	151835.7	249928.3	325116.1	260022.0	144978.1	90398.9	177049.4
321	De Grey	11.0	11.0	11.0	11.0	11.0	11.0	11.0	11.0	11.0	11.0	11.0	11.0	11.0
322	Paraiba Do Sul	8096.7	7902.6	9838.7	14156.2	11371.2	13191.8	16284.0	18864.9	14871.9	14426.4	8900.6	7285.8	12099.2
323	Fortescue	9.8	9.8	10.1	10.2	10.2	10.0	10.1	10.3	10.5	10.7	10.5	10.2	10.2
324	Mangoky	36833.8	29713.2	51243.5	62417.9	19314.3	3491.1	3051.9	3056.3	3009.5	3297.2	2252.2	12760.5	19203.4
325	Fitzroy	6135.8	9673.2	46656.6	47241.2	30687.5	22800.7	29204.9	39198.8	53174.6	56851.7	42674.6	29016.8	34443.0
326	Orange	122068.9	208326.4	240318.1	173615.7	83286.4	99538.7	127434.9	195558.1	238623.5	235041.8	144597.6	110597.0	164917.3
327	Ashburton	10.1	10.1	10.1	10.1	10.1	10.1	10.1	10.1	10.1	10.1	10.1	10.1	10.1
328	Gascoyne	16.1	26.1	37.7	31.0	21.7	16.8	14.0	28.0	40.4	42.4	37.7	32.1	28.7
329	Rio Ribeira Do Iguape	2155.1	2072.6	2145.4	2360.0	2087.9	2051.4	2090.6	2154.1	2099.0	2084.0	2155.9	2158.7	2134.6
330	Incomati	6744.6	10034.5	17801.4	28995.5	20121.8	20976.2	24492.5	35427.8	44482.0	30139.7	16687.2	10339.6	22186.9
331	Murray	1796675	1629509	1677274	851276	281547	141682	147848	291593	566450	902256	998880	1224788	875815
332	Murchison	19.2	30.4	35.3	27.4	16.5	13.9	12.6	22.6	35.9	41.7	37.9	30.9	27.0
333	Maputo	1657.9	1863.0	6406.3	16175.1	15406.1	17843.1	19266.3	28775.0	32439.2	26730.3	13692.5	9878.1	15844.4
334	Uruguay	674173.3	302717.3	105062.7	9149.2	4500.7	4538.6	4675.7	6884.4	7069.2	75991.9	222852.6	345000.7	146884.7
335	Tugela	12844.4	32307.0	51653.7	31877.5	17955.0	18187.8	24585.7	38346.7	45657.8	41740.0	25547.1	13318.7	29501.8
336	Colorado (Argentinia)	164413.7	128020.2	103199.4	45559.1	35233.9	27627.6	63043.9	118507.8	170003.6	212465.5	229930.1	151341.9	120778.9
337	Rio Jacui	259052.2	109321.3	47759.7	2362.1	2136.7	2126.9	2125.6	2146.1	2192.2	28141.6	82757.9	131107.2	55935.8
338	Huasco	880.5	669.4	596.5	320.6	233.5	377.0	420.7	900.5	1491.4	1703.2	575.0	678.5	737.2
339	Limari	33036.7	20409.8	15231.9	4343.8	2483.7	1128.7	1359.8	4913.1	11224.6	15815.0	12289.3	17019.2	11604.6
340	Negro (Uruguay)	77510.1	40088.3	24714.6	1701.7	169.3	169.0	169.1	173.3	185.6	2311.3	17959.6	32370.9	16460.2
341	Groot-Vis	14526.3	32500.4	31051.4	18743.8	15831.9	12788.2	13412.8	17802.9	29350.8	36957.0	25499.1	25607.1	22839.3
342	Salado	30690.2	31714.2	12777.0	4173.2	2776.8	2699.5	3138.3	3343.9	6448.3	7055.3	7087.4	7698.1	9966.8
343	Blackwood	677.8	868.2	921.3	540.3	112.5	58.0	56.5	62.1	86.6	400.3	621.9	777.6	431.9
344	Rapel	160994.9	92143.4	63877.9	14339.2	2676.0	1202.5	1193.5	3295.0	25200.3	76379.7	56481.3	90362.1	49012.2
345	Negro (Argentinia)	22754.5	22634.3	16810.0	7621.7	3348.7	1700.5	2238.0	4931.8	8688.9	13110.6	21027.1	23140.1	12333.9
346	Biobio	28414.2	15065.1	11595.8	3916.5	5844.3	5712.0	5919.4	6177.5	11895.2	15852.7	5911.7	15700.7	11000.4
347	Waikato	805.3	884.8	1470.9	936.2	772.1	771.2	771.2	771.2	771.3	772.8	793.6	862.8	865.3
348	South Esk	4363.8	7720.4	9018.2	3485.7	1054.4	242.4	114.1	376.1	1709.8	3617.7	4179.9	5909.2	3482.6
349	Chubut	4565.2	9149.9	10672.0	6480.2	3205.9	1252.6	1584.8	3762.2	6442.3	9430.3	10387.5	10851.3	6482.0
350	Clutha	2540.9	17233.5	17807.5	11223.5	1629.9	204.3	188.7	925.2	7142.1	10489.7	13228.7	10746.4	7780.0
351	Baker	25.4	66.4	107.7	64.7	33.7	27.0	29.0	38.7	70.2	137.0	122.6	111.6	69.5
352	Santa Cruz	29.0	136.8	175.6	95.3	34.8	18.5	17.8	36.9	91.7	181.2	210.6	166.2	99.5
353	Ganges	13158709	14044732	19911407	12435642	10053368	5544274	3941654	2382353	3662445	7534298	11330417	6754745	9229504
354	Salween	18910.2	21222.2	34782.4	73001.7	71107.9	75090.7	40034.7	32003.0	47800.8	46577.3	20138.6	13490.7	41180.0
355	Hong(Red River)	86812.4	94924.2	106398.9	217194.1	516481.4	257551.0	115094.1	89732.2	76564.6	35708.5	39272.1	55205.2	140911.5
356	Lake Chad	123841.3	182360.2	195207.9	192937.9	45619.4	47440.0	34137.9	24381.8	34770.1	57186.8	45197.3	63649.7	87227.5
357	Okavango	850.3	939.6	1358.4	2321.6	2706.3	2777.0	3170.3	4094.5	4792.1	4473.7	2363.9	1472.9	2610.1
358	Tarim	46367	103636	364031	795438	1367750	1424510	1536483	1380857	981231	229859	96323	39023	697126
359	Horton	0.2	0.2	0.2	0.2	0.2	0.2	0.2	0.2	0.2	0.2	0.2	0.2	0.2
360	Hornaday	0.4	0.4	0.4	0.4	0.4	0.4	0.4	0.4	0.4	0.4	0.4	0.4	0.4
361	Conception	863.2	2402.2	4812.7	6493.5	5091.4	6316.2	5835.4	8031.6	7388.1	5674.9	2054.4	1500.2	4705.3
362	Ulua	7388.7	5158.9	11597.1	15430.8	9677.7	6025.2	3696.0	2598.1	1529.4	873.7	1227.4	5331.4	5877.9
363	Patacua	684.0	319.0	962.4	1424.5	722.7	403.1	517.0	290.0	288.6	149.5	167.7	575.7	542.0
364	Coco	694.7	582.3	1181.1	1487.5	561.4	289.3	280.7	274.2	256.1	223.7	233.4	511.1	548.0
365	Ocona	1388.9	1328.2	1458.9	5571.7	6039.9	2802.8	1669.3	2565.0	3477.2	3202.9	2475.2	1547.2	2793.9
366	Cuanza	868.5	1178.4	738.4	660.0	1308.7	2689.8	3287.9	4779.9	5424.3	5051.3	1988.5	1595.4	2464.3
367	Cunene	184.8	215.9	172.6	214.8	387.3	485.7	548.8	643.5	658.0	475.7	299.6	198.5	373.8
368	Doring	13480.3	27491.8	34498.5	19964.9	5689.1	2413.1	2085.1	6704.6	22058.4	44962.5	32514.2	28071.5	19994.5
369	Gamka	2715.0	13051.7	17664.1	12790.1	7396.2	6211.1	4523.1	5661.0	15664.3	22727.4	12638.5	11656.3	11058.3
370	Groot- Kei	2443.3	5945.1	6908.7	4971.3	4848.2	4427.5	5461.1	7147.2	9965.5	10746.6	7337.2	5007.8	6267.5
371	Lurio	42.1	42.1	42.5	65.9	79.2	78.9	85.1	104.1	120.3	121.2	72.7	49.4	75.3
372	Messalo	9.9	9.7	10.0	10.2	15.2	15.4	25.7	41.2	55.8	55.8	13.7	10.9	22.8
373	Rovuma	368.1	278.2	236.9	380.9	604.4	438.8	401.0	464.3	501.0	509.2	345.7	331.1	405.0
374	Galana	4086.6	4402.9	2121.7	1109.4	1314.2	2968.8	4794.7	5148.0	4916.8	4405.9	1807.1	2100.7	3264.7
375	Pyasina	462.7	462.7	462.7	462.7	462.7	462.7	462.7	462.7	462.7	462.7	462.7	462.7	462.7
376	Popigay	1.6	1.6	1.6	1.6	1.6	1.6	1.6	1.6	1.6	1.6	1.6	1.6	1.6
377	Fuchun Jiang	9387.7	9389.8	9394.0	14416.1	31425.8	25890.3	120394.7	93993.7	98617.9	20451.1	10449.3	9426.9	37769.8
378	Min Jiang	8168.5	8157.3	8156.7	8768.9	14877.9	16995.3	92286.1	71040.8	63069.9	11420.9	9325.7	8428.1	26724.7
379	Han Jiang	8406.5	8240.3	8206.6	9470.7	17858.6	21719.8	60040.3	39510.8	41152.2	10352.2	9234.2	8624.0	20234.7
380	Mamberamo	123.4	123.9	123.3	123.0	123.1	123.5	123.2	124.1	126.2	126.8	125.8	124.6	124.2
381	Lorentz	4.5	4.6	4.5	4.5	4.5	4.5	4.6	4.6	4.7	4.7	4.6	4.5	4.6
382	Eilanden	15.9	16.5	15.9	15.6	15.6	15.6	15.9	16.0	16.6	16.9	17.3	16.6	16.2
383	Uwimbu	16.9	18.2	19.4	18.5	22.2	29.7	38.1	42.2	40.7	34.4	27.9	20.3	27.4
384	Sungai Kajan	412.8	124.0	27.6	27.7	28.2	29.3	30.4	28.2	556.1	441.0	119.7	276.4	175.1
385	Sungai Mahakam	1603.6	820.3	288.3	267.0	293.8	278.8	503.9	607.6	3384.0	2255.0	1350.9	990.5	1053.6
386	Sungai Kapuas	1123.8	772.5	494.9	455.4	463.5	467.5	502.7	491.3	1104.4	745.6	646.5	654.6	660.2
387	Batang Kuantan	21554.5	8941.9	831.1	965.8	1148.5	2243.1	2968.7	3059.6	16614.0	17601.1	14363.5	10111.4	8366.9
388	Batang Hari	18804.9	8425.0	1056.0	990.9	1605.4	2782.2	3403.0	4321.5	17731.0	17859.5	12626.0	9336.6	8245.2
389	Flinders	13.9	26.6	130.2	192.0	176.5	146.7	162.3	198.3	236.2	237.2	172.6	113.5	150.5
390	Leichhardt	13.2	13.8	24.0	31.9	31.2	28.1	29.8	33.5	36.8	36.4	29.4	22.0	27.5
391	Escaut (Schelde)	28581.6	28581.6	28582.3	28814.5	31907.5	33659.4	36561.2	38970.5	32038.1	28958.6	28581.6	28581.6	31151.5
392	Issyk-Kul	3869.6	3867.4	5610.0	85052.7	331331.6	551793.0	652012.1	682351.3	487278.5	193577.7	23889.9	4687.0	252110.1
393	Balkhash	7898.1	7903.9	26411.0	176009.3	367687.4	451268.4	660660.3	716715.5	543809.3	148355.0	15538.2	8438.0	260891.2
394	Eyre Lake	256.3	391.9	803.8	738.9	643.9	558.2	597.0	749.8	900.6	923.5	721.1	586.0	655.9
395	Lake Mar Chiquita	73618.5	72832.9	50326.6	42798.2	21882.7	24157.2	34910.8	46837.8	66462.2	55393.0	44846.3	30999.3	47088.8
396	Lake Turkana	16702.2	12196.8	6961.2	1990.2	2639.1	2532.7	3148.7	7388.1	8373.8	6303.4	4962.7	8450.8	6804.2
397	Dead Sea	4976.7	10605.9	46781.5	124014.2	191001.2	180841.4	209018.1	193278.0	109862.3	66696.8	33941.2	16173.1	98932.5
398	Suriname	72.8	72.8	72.8	72.8	72.8	72.8	72.8	72.8	72.8	72.8	72.8	72.8	72.8
399	Lake Titicaca	54629.1	39854.3	30463.0	25263.3	17817.7	15135.1	12497.3	15948.5	17203.3	18560.0	20898.8	30440.3	24892.6
400	Lake Vattern	717.8	717.8	717.8	719.3	853.4	993.5	1190.2	1358.0	955.5	726.5	717.8	717.8	865.4
401	Great Salt Lake	11288.1	11334.6	26872.7	108482.1	190196.5	290908.9	355162.4	295952.7	178346.4	99340.6	32184.9	13326.8	134449.7
402	Lake Taymur	11.7	11.7	11.7	11.7	11.7	11.7	11.7	11.7	11.7	11.7	11.7	11.7	11.7
403	Daryacheh-Ye Orumieh	4126.5	7750.8	33647.9	83546.7	100958.4	133857.5	161670.1	185227.9	115943.3	52188.0	17973.6	7598.4	75374.1
404	Van Golu	847.5	847.7	993.1	1806.4	8779.5	18522.7	17687.8	18856.2	13419.3	4618.4	1624.0	959.4	7413.5
405	Ozero Sevan	1005.7	1005.7	1084.3	1191.3	1787.3	4689.9	6576.3	7338.7	4115.2	2066.8	1122.2	1040.9	2752.0
