# Supplementary material for: Global Monthly Water Scarcity: Blue Water Footprints versus Blue Water Availability
Source: PLoS One. 2012 Feb 29;7(2):e32688. doi: 10.1371/journal.pone.0032688 (PMC3290560; doi:10.1371/journal.pone.0032688)
Supplement: Table S2 — Monthly natural runoff for the world's major river basins. (PDF) [file pone.0032688.s006.pdf]

Table S2. Monthly natural runoff for the world's major river basins

Basin ID	Basin name	Area (km^2)	Natural runoff (Mm^3/month)												
			Jan	Feb	Mar	Apr	May	Jun	Jul	Aug	Sep	Oct	Nov	Dec	Average
1	Khatanga	294907.5	1571.7	58.7	35.4	21.4	221.2	25398.5	12884.8	6944.9	4357.0	2419.0	1461.0	882.4	4688.0
2	Olenek	208522.0	1231.0	159.8	96.5	58.3	658.3	16431.9	5510.3	3070.3	1870.4	1106.6	668.3	403.7	2605.5
3	Anabar	85015.5	525.8	85.3	51.5	31.1	18.8	4128.1	2082.3	1011.7	602.5	354.9	214.4	129.5	769.6
4	Yana	233479.4	896.1	72.8	44.0	26.6	159.2	7535.9	6820.7	3807.3	1832.6	1106.8	668.5	403.8	1947.9
5	Yenisei	2558237.3	16135.2	742.2	452.4	7752.7	162714.9	162047.8	97190.0	64240.9	48091.7	24440.8	14455.9	8735.1	50583.3
6	Indigirka	341227.8	1902.5	179.7	108.5	65.6	450.9	16809.8	14507.5	6786.0	3631.1	2176.2	1314.4	793.9	4060.5
7	Lena	2425551.1	15771.8	655.1	396.4	361.9	87091.9	124907.8	84650.4	63046.2	53636.1	23839.3	14390.4	8692.2	39786.6
8	Omoloy	38871.3	26.9	0.7	0.4	0.2	0.2	426.1	277.1	128.9	73.6	43.7	26.4	15.9	85.0
9	Tana (NO, FI)	14518.1	71.8	0.0	0.0	0.0	2851.4	778.3	457.0	276.1	217.4	149.0	77.9	47.1	410.5
10	Colville	57544.7	185.8	1.4	0.8	0.5	45.5	1938.0	1977.6	1034.6	579.1	324.5	196.0	118.4	533.5
11	Alazeya	85493.3	184.1	31.2	18.9	11.4	6.9	1896.8	555.0	314.2	189.8	114.6	69.2	41.8	286.2
12	Anderson	66491.7	54.7	0.4	0.2	0.1	2548.6	797.3	434.9	262.7	158.7	95.8	57.9	35.0	370.5
13	Kolyma	652850.5	3721.1	160.9	97.2	58.8	5342.4	25441.2	35941.4	16207.8	10517.4	5575.9	3367.8	2034.2	9038.8
14	Tuloma	26057.7	94.6	11.6	7.1	4.4	1732.1	547.3	300.0	180.4	119.2	121.3	55.2	33.5	267.2
15	Muonio	37346.5	143.9	13.1	8.0	213.9	2005.5	1110.4	750.1	388.4	294.9	187.3	101.6	61.4	439.9
16	Yukon	829632.3	4850.3	252.5	152.7	943.7	53166.5	48766.9	29776.4	16607.0	12906.1	7648.1	4212.2	2544.3	15152.2
17	Palyavaam	31112.8	106.3	0.0	0.0	0.0	0.0	1878.7	1036.4	526.4	355.2	191.1	115.4	69.7	356.6
18	Kemijoki	55824.7	487.2	3.2	2.0	594.1	8987.0	2448.1	1458.1	934.7	1082.1	1366.4	516.4	312.0	1515.9
19	Mackenzie	1752001.5	5637.8	86.9	53.5	9063.7	79403.5	79172.3	46002.0	24877.4	15732.9	10447.5	5767.0	3484.1	23310.7
20	Noatak	32319.5	156.5	0.8	0.5	0.3	401.9	1980.3	1099.7	624.3	629.7	275.6	166.5	100.6	453.1
21	Anadyr	171275.8	1182.1	1.3	0.8	0.5	2452.2	21998.7	10380.5	5461.5	4105.7	2116.1	1278.1	771.9	4145.8
22	Pechora	312763.3	2459.1	31.2	19.2	1449.0	58305.9	38794.9	16270.2	9630.9	7855.7	4450.1	2542.8	1536.1	11945.4
23	Lule	25127.6	313.1	1.0	0.6	1119.1	3851.3	3784.3	1895.4	1112.2	951.7	683.0	335.9	202.9	1187.5
24	Kalixaelven	17157.6	78.3	6.6	4.0	388.6	1237.8	757.3	351.5	203.1	137.7	118.6	57.6	34.8	281.3
25	Ob	2701040.7	6930.1	198.1	135.9	80842.2	148619.4	68228.3	36839.8	23540.4	18275.7	14074.8	6865.0	4161.9	34059.3
26	Ellice	12599.6	30.5	0.0	0.0	0.0	0.0	958.3	248.8	150.3	90.8	54.8	33.1	20.0	132.2
27	Taz	152086.0	989.9	6.0	3.6	2.2	12922.1	23163.4	7321.6	4370.1	3193.2	1738.0	1049.7	634.0	4616.2
28	Kobuk	30242.4	211.1	31.8	19.2	11.6	2063.7	1100.6	619.7	373.5	338.6	159.6	96.4	58.2	423.7
29	Coppermine	43016.4	28.9	0.1	0.1	0.0	390.0	714.8	246.4	139.9	84.5	51.0	30.8	18.6	142.1
30	Hayes(Trib. Arctic Ocean)	22992.8	27.0	0.0	0.0	0.0	0.0	834.5	225.2	133.1	80.4	48.5	29.3	17.7	116.3
31	Pur	111351.3	740.7	0.3	0.3	0.3	7186.7	15161.5	4615.2	2795.1	2896.5	1331.2	804.1	485.8	3001.5
32	Varzuga	8182.2	47.2	0.0	0.0	0.0	683.9	188.7	110.1	66.6	112.4	140.1	51.2	30.9	119.3
33	Ponoy	13186.0	127.2	0.0	0.0	0.0	1585.1	438.1	255.8	178.3	268.5	394.1	138.1	83.4	289.1
34	Kovda	10227.6	30.6	0.0	0.0	0.0	881.9	280.3	152.3	92.5	71.2	78.0	33.2	20.1	136.7
35	Back	141351.9	327.2	0.0	0.0	0.0	5571.3	8215.3	2637.8	1590.9	990.4	588.0	355.2	214.5	1707.6
36	Kem	42080.8	235.3	0.5	0.4	2642.5	3902.2	1352.6	781.6	490.1	429.8	696.2	253.6	153.2	911.5
37	Nadym	54624.7	383.2	0.2	0.1	0.1	4504.7	7346.2	2354.4	1461.2	1502.8	687.7	415.4	250.9	1575.6
38	Quoich	28217.6	41.6	0.0	0.0	0.0	0.0	1216.3	349.6	199.5	128.2	74.8	45.2	27.3	173.5
39	Mezen	76715.3	372.9	4.5	2.7	3916.9	10521.7	3855.7	2081.0	1250.6	799.5	867.4	386.3	233.3	2024.4
40	Iijoki	16163.3	94.2	0.1	0.1	1326.2	997.0	396.0	232.9	151.1	155.9	299.9	102.3	61.8	318.1
41	Joekulsa A Fjoellum	7311.0	75.0	0.0	0.0	5.7	754.0	592.9	236.8	148.0	147.0	221.4	82.3	49.2	192.7
42	Svarta, Skagafiroi	3429.6	54.9	0.0	29.3	123.6	363.8	392.6	148.0	89.0	76.1	152.2	68.9	36.0	127.9
43	Oulujoki	30554.5	230.9	1.1	0.8	5398.3	1702.8	939.7	562.7	373.1	416.6	710.7	247.3	149.5	894.5
44	Lagarfljot	3285.3	112.9	0.0	0.0	22.3	1190.7	742.9	324.1	231.6	257.6	309.8	128.1	74.0	282.9
45	Thelon	238839.0	478.4	0.1	0.0	0.0	6190.9	10749.7	3370.2	2026.8	1686.6	859.5	519.1	313.5	2182.9
46	Angerman	32372.0	343.5	0.5	0.4	4027.0	3524.6	2685.5	1232.5	833.4	764.3	954.8	371.0	224.1	1246.8
47	Thjorsa	7527.1	422.1	33.1	91.6	1123.0	1723.6	1322.4	662.2	584.0	668.7	840.8	478.5	288.8	686.6
48	Northern Dvina(Severnaya	323573.1	1021.4	35.1	22.2	44567.2	19378.9	9541.8	5592.3	3376.1	2058.6	2097.6	971.8	587.9	7437.6
49	Oelfusa	5678.3	378.5	0.0	323.1	1726.6	690.3	685.2	565.6	398.0	471.2	658.7	434.6	320.1	554.3
50	Nizhny Vyg (Soroka)	31334.1	206.5	0.1	0.1	5451.2	1622.9	926.7	553.6	334.4	420.4	626.2	224.2	135.5	875.1
51	Kuskokwim	118114.0	1269.5	1.4	0.9	0.5	16620.3	7998.3	5470.1	5473.3	5291.7	2398.6	1372.5	829.0	3893.8
52	Vuoksi	62707.4	334.8	1.2	1.2	10528.9	2968.3	1728.3	1039.2	643.3	560.3	960.4	387.0	220.0	1614.4
53	Onega	65894.0	224.9	2.8	1.8	10633.8	3402.5	1902.0	1125.9	680.1	436.9	556.4	234.8	141.1	1611.9
54	Susitna	49470.3	1270.7	2.2	1.4	3654.1	8319.0	8905.7	5269.7	4095.0	4842.3	2738.5	1370.5	827.8	3441.4
55	Kymijoki	33623.1	186.3	1.0	1.0	4570.7	1319.9	758.1	456.3	277.2	216.4	308.4	311.4	122.5	710.7
56	Neva	223309.5	1195.2	10.6	8.8	32169.2	9560.0	5468.9	3272.4	1984.2	1703.3	2586.4	1686.6	773.9	5034.9
57	Ferguson	15200.4	40.3	0.0	0.0	0.0	0.0	1053.5	296.1	171.1	142.1	72.5	43.8	26.4	153.8
58	Copper	64959.7	1090.8	0.0	0.0	17.3	7546.7	10214.6	7440.5	4185.8	3963.1	2215.5	1184.1	715.2	3214.5
59	Gloma	42862.7	563.2	1.3	12.0	3439.3	3368.1	3615.4	2048.4	1487.9	1400.5	1344.1	677.6	369.7	1527.3
60	Kokemaenjoki	26615.9	236.7	1.6	1.5	3662.2	1092.9	616.1	371.5	225.5	147.2	152.9	501.9	154.8	597.1
61	Vaenern-Goeta	51791.5	1198.3	3.2	2259.8	5377.8	2297.5	1379.5	819.2	707.0	791.8	1475.7	1616.9	1037.2	1580.3
62	Thlewiaza	64399.6	91.9	10.3	6.2	3.7	1834.1	666.0	339.5	204.2	198.6	94.1	56.8	34.3	295.0
63	Alsek	28422.0	286.1	0.0	0.0	584.4	2611.7	2770.9	1100.4	738.6	961.8	680.0	310.6	187.6	852.7
64	Volga	1408278.9	3091.7	147.2	747.6	140132.1	51088.9	28581.8	17230.0	10747.1	6887.4	6486.8	3165.6	1902.0	22517.4
65	Dramselv	17364.0	259.8	0.5	97.0	1233.4	1458.1	1401.1	734.0	665.9	720.2	633.0	304.9	170.5	639.9
66	Arnaud	44931.9	564.3	0.0	0.0	0.0	632.5	5641.8	2064.4	1543.3	1878.6	1362.9	612.6	370.0	1222.5
67	Nushagak	29513.6	517.3	0.0	0.0	1308.4	4936.8	1770.2	1042.6	1293.2	1548.5	1382.8	561.6	339.2	1225.1
68	Seal	53439.9	126.4	1.6	1.0	0.6	3534.7	1434.9	728.5	432.3	415.7	241.7	130.6	78.9	593.9
69	Taku	17967.6	430.1	0.0	0.0	806.8	2508.0	2296.9	1033.2	783.7	1027.7	1263.6	467.0	282.0	908.3
70	Narva	58147.0	710.7	1.2	1.2	6018.5	1937.0	1079.4	639.7	420.0	372.9	731.8	1459.3	466.4	1153.2
71	Stikine	51147.5	1826.9	603.3	508.0	1656.9	9699.8	11933.0	5766.5	3728.6	3815.5	3379.2	1948.6	1370.5	3853.1
72	Churchill	298505.0	789.5	20.1	12.3	3400.0	13120.8	7724.3	3761.6	2131.7	1960.1	1724.5	774.9	468.2	2990.7
73	Feuilles (Riviere Aux)	37425.3	673.6	0.0	0.0	0.0	2539.5	5199.5	2051.0	1757.3	1931.8	1773.6	731.3	441.7	1424.9
74	George	39054.1	758.1	0.0	0.0	0.0	4958.0	5039.7	3961.6	2616.4	2618.4	1701.2	823.0	497.1	1914.5
75	Caniapiscau	105690.6	2297.2	0.9	0.5	0.3	16170.7	12475.4	6805.5	6229.8	6669.5	6024.1	2490.3	1504.1	5055.7
76	Western Dvina (Daugava)	89340.3	772.7	2.1	2.1	9180.8	2977.1	1713.6	1016.3	619.6	425.3	849.3	1548.2	507.4	1634.5
77	Aux Melezes	41384.1	637.5	0.1	0.1	0.0	5433.5	3250.9	1841.6	1727.2	1787.2	1696.1	691.6	417.7	1457.0
78	Baleine, Grande Riviere De	22136.1	379.8	0.0	0.0	0.0	4270.1	1505.8	1122.0	983.1	1130.8	994.3	412.3	249.0	920.6
79	Spey	2942.2	478.1	203.5	174.9	136.5	98.8	58.1	43.8	52.5	81.3	157.4	246.1	292.1	168.6
80	Kamchatka	54103.9	689.9	0.0	0.0	0.0	9603.9	7199.7	5399.2	2604.2	1918.9	1546.8	749.0	452.4	2513.7
81	Nass	21211.7	1054.0	0.0	810.7	3441.7	6020.4	4634.1	2047.3	1515.8	1839.7	2607.5	1420.3	691.1	2173.5
82	Skeena	42944.4	1078.2	0.2	1041.9	4491.6	8819.6	7160.8	3082.5	2010.7	1937.6	2313.5	1518.6	707.1	2846.9
83	Nelson	1099380.3	1698.4	40.6	35.8	29311.7	20864.5	15143.9	8074.5	5014.9	4579.4	4110.0	1799.8	1090.5	7647.0
84	Hayes(Trib. Hudson Bay)	105371.9	339.8	7.0	4.3	2.6	7011.9	3778.4	2054.7	1111.5	798.0	731.1	339.8	205.3	1365.4
85	Gudena	2860.9	259.3	115.5	103.3	77.1	41.9	24.7	16.2	10.7	14.8	58.0	124.8	157.3	83.6
86	Skjern A	2817.6	351.5	132.9	121.1	89.0	50.8	28.9	18.4	22.4	70.4	165.2	192.5	228.6	122.6
87	Neman	97299.9	740.2	3.4	2540.2	8813.4	3141.2	1745.1	1037.9	641.8	406.1	467.3	1597.2	486.5	1801.7
88	Fraser	239678.4	3955.1	1048.3	2360.7	15356.9	26897.1	15496.8	7432.2	4439.9	3254.0	3618.6	3140.5	2506.4	7458.9
89	Severn(Trib. Hudson Bay)	98590.5	593.2	0.0	0.0	969.8	7964.8	3325.3	1813.8	1158.6	1429.9	1689.2	644.0	388.9	1664.8
90	Amur	2023520.4	13098.2	58.9	59.3	41918.1	57294.3	53865.4	45446.7	49807.8	50357.7	28455.4	14176.3	8572.4	30259.2
91	Tweed	4770.9	575.8	239.8	225.1	158.2	110.7	72.3	54.4	61.3	91.1	172.0	311.6	366.0	203.2
92	Grande Riviere De La Bale	24256.7	482.1	0.0	0.0	0.0	3057.6	1978.3	1140.9	1097.4	1241.0	1368.9	523.3	316.1	933.8
93	Grande Riviere	111718.4	2729.2	0.9	0.5	0.3	20738.4	10192.2	6544.7	6144.0	7187.4	7682.4	2959.2	1787.3	5497.2
94	Winisk	106470.3	890.2	2.1	1.3	3470.5	10190.2	4433.5	2449.4	1479.2	2350.9	2486.7	957.9	578.6	2440.9
95	Churchill, Fleuve (Labrador	84984.3	1702.6	0.0	0.0	0.0	15223.9	10971.0	6028.7	4689.5	4563.7	4517.0	1848.4	1116.4	4221.8
96	Dniepr	510661.3	849.5	45.1	9860.5	20513.6	7860.1	4484.4	2811.4	1783.2	1063.7	891.9	1449.4	588.9	4350.1
97	Ural	339084.2	324.2	5.7	165.2	10759.3	3817.4	2162.0	1470.8	948.2	527.8	322.1	587.8	214.5	1775.4
98	Wisla	193764.0	947.0	37.8	12812.8	6810.9	4190.9	2577.5	1781.3	1243.2	858.0	779.6	1157.5	847.9	2837.0
99	Don	425629.6	308.1	29.4	4138.3	14368.8	5709.0	3280.6	2272.7	1517.1	801.0	444.2	431.0	212.1	2792.7

Basin ID	Basin name	Area (km^2)	Natural runoff (Mm^3/month)												
			Jan	Feb	Mar	Apr	May	Jun	Jul	Aug	Sep	Oct	Nov	Dec	Average
100	Oder	116536.3	1032.5	1852.6	5896.3	3319.4	2160.7	1462.2	947.3	682.0	505.5	478.7	627.0	1046.2	1667.5
101	Elbe	139347.6	2858.7	2594.5	4899.4	3701.9	2272.5	1577.0	1147.9	892.7	725.8	865.4	1454.7	1857.4	2070.7
102	Trent	9052.9	691.4	368.2	303.2	213.3	137.6	80.5	56.5	51.0	49.0	67.1	198.0	402.5	218.2
103	Weser	43140.2	3511.3	2008.3	1895.7	1397.8	902.7	617.5	504.3	484.0	501.8	819.6	1601.5	2294.1	1378.2
104	Attawapiskat	30457.4	121.7	0.0	0.0	0.0	2195.5	613.1	355.5	214.7	350.3	329.0	132.1	79.8	366.0
105	Eastmain	48837.5	1182.9	0.0	0.0	2558.1	9188.6	4899.7	2922.3	2536.0	2987.8	3379.4	1284.2	775.6	2642.9
106	Manicouagan (Riviere)	54205.4	1252.8	0.3	0.2	524.6	8666.6	6924.6	3691.1	2983.3	3220.2	3479.9	1358.9	820.8	2743.6
107	Columbia	668561.9	11960.9	11259.5	20903.3	38188.0	55190.6	36849.4	18551.8	11683.5	7290.7	5462.2	6682.2	7923.4	19328.8
108	Little Mecatina	17902.9	444.5	0.0	0.0	0.0	6156.3	2178.5	1505.7	1078.9	1004.0	1263.0	482.6	291.5	1200.4
109	Natashquan (Riviere)	16948.2	291.0	0.0	0.0	156.2	3726.8	1620.1	1208.4	808.1	691.2	792.3	315.9	190.8	816.7
110	Rhine	190522.1	13179.1	7657.6	8094.3	9602.3	8013.0	6055.1	4779.6	4183.7	3971.9	4514.6	6546.6	8363.7	7080.1
111	Albany	123081.0	813.0	0.1	0.1	8411.8	9204.2	3827.2	2148.3	1306.1	1776.4	2432.0	882.5	533.1	2611.2
112	Saguenay (Riviere)	91366.9	1984.2	1.5	1.5	11245.1	11251.7	8137.7	5058.6	4100.4	4665.6	5748.5	2173.4	1301.6	4639.2
113	Thames	12358.9	726.2	447.7	361.1	237.6	136.9	78.4	49.7	32.4	21.8	18.4	131.8	395.2	219.8
114	Nottaway	118709.0	2188.3	0.2	0.2	13451.3	15987.7	7640.1	5188.1	4271.3	5083.6	6291.3	2438.0	1434.9	5331.3
115	Rupert	16063.4	311.4	0.0	0.0	794.7	3201.0	1189.1	796.1	677.5	758.7	894.3	338.0	204.2	763.8
116	Moose(Trib. Hudson Bay)	105615.2	1000.2	0.6	0.6	11334.0	9979.0	4300.8	2527.6	1594.4	2136.5	3024.6	1085.8	656.0	3136.7
117	St.Lawrence	1055021.5	13835.1	351.1	29605.6	132375.7	51230.9	31947.4	19602.9	13031.9	15304.9	21038.1	22898.9	9715.3	30078.1
118	Danube	793704.8	15369.2	12969.9	30056.7	34399.1	27077.0	19150.4	14083.1	11248.0	10213.5	12685.9	15065.0	12575.3	17907.8
119	Seine	74227.9	3426.9	2491.0	2183.7	1663.9	1005.1	594.9	402.4	304.5	202.7	234.2	696.1	1700.9	1242.2
120	Dniestr	72108.2	407.9	13.3	3147.7	2602.1	1528.5	1130.4	792.2	626.7	510.8	629.4	702.3	272.1	1030.3
121	Southern Bug	60121.0	38.8	7.9	1840.9	967.0	519.4	311.3	205.6	138.9	77.0	43.3	27.5	18.1	349.6
122	Mississippi	3196605.4	79924.0	66571.6	111936.2	102147.6	83552.9	56877.9	36359.7	27329.8	18097.4	12199.9	20013.0	38932.9	54495.2
123	Skagit	7961.0	951.3	342.8	1452.8	1747.8	975.0	491.1	287.0	173.6	108.5	409.0	751.3	600.5	690.9
124	Aral Drainage	1233148.5	2546.9	4161.6	13784.1	20140.9	23937.1	21375.9	17965.9	12930.4	8104.3	3850.2	1674.4	1541.5	11001.1
125	Loire	115943.6	5691.9	3966.2	3912.1	3192.9	2280.7	1475.0	937.1	710.2	546.1	804.6	1839.3	3262.7	2384.9
126	Rhone	97485.2	7316.8	3365.1	5895.8	6325.3	5588.7	4446.6	2690.2	2212.7	2277.1	3477.1	5232.4	5154.7	4498.5
127	Saint John	55151.8	1543.0	1.9	1.9	13364.2	4478.5	3060.9	1929.0	1260.9	1384.3	2179.7	2871.4	1012.4	2757.4
128	Po	73066.6	4276.6	2000.0	3536.1	5530.3	6397.4	4452.6	2941.3	2394.4	2314.0	2947.8	3482.3	2857.2	3594.2
129	Penobscot	21168.9	655.1	0.6	482.1	5554.7	1878.0	1224.9	733.4	451.5	421.6	684.5	1327.0	429.8	1153.6
130	St.Croix	4638.6	170.5	0.1	0.1	1441.8	475.5	301.5	171.5	101.8	88.6	166.9	352.7	111.8	281.9
131	Kuban	58935.7	1008.9	1275.5	1664.7	2123.9	1943.7	1594.5	1393.7	826.2	621.9	471.0	528.0	626.0	1173.2
132	Connecticut	27468.3	934.3	7.8	3116.2	4979.1	2528.5	1556.0	985.4	660.3	761.0	1049.6	1699.4	665.0	1578.5
133	Liao He	194436.5	678.2	20.4	220.6	1657.1	2266.7	2171.0	2661.2	3988.1	2606.5	1383.3	782.0	454.6	1574.1
134	Garonne	55807.2	3122.4	1918.1	2169.4	2289.6	1911.6	1113.4	759.2	594.0	474.6	657.6	1074.9	1915.8	1500.0
135	Ishikari	13783.3	859.4	2.4	2.4	4390.2	1918.3	1080.5	793.8	776.5	1178.1	1434.9	1481.8	564.3	1206.9
136	Merrimack	12645.1	381.1	8.4	3157.8	1789.4	1035.9	634.7	377.8	233.8	212.7	361.4	783.0	252.8	769.1
137	Hudson	36892.8	1000.4	26.3	4477.0	4970.6	2745.5	1656.2	1063.5	725.6	742.5	1044.8	1764.9	728.3	1745.5
138	Colorado(Pacific Ocean)	640463.6	323.3	99.7	738.0	3046.4	5903.7	4320.1	2390.6	1653.7	1126.8	740.4	370.5	231.4	1745.4
139	Klamath	40040.1	3010.5	3574.1	3546.4	3046.4	2001.6	1051.5	698.7	455.6	276.0	146.5	394.9	1495.2	1641.5
140	Ebro	85158.6	4220.1	2820.2	2785.8	2876.1	2619.8	1631.9	1192.1	853.9	509.9	561.7	873.7	2424.5	1947.5
141	Rogue	14526.6	1090.2	1088.4	909.0	858.5	622.6	302.4	190.5	120.0	73.0	41.5	212.2	514.3	501.9
142	Douro	96125.4	3913.4	3031.8	4104.3	2983.9	2057.4	1252.2	1050.6	820.4	404.1	215.0	539.3	1650.2	1835.2
143	Susquehanna	69080.1	2091.9	1240.2	8814.7	5917.2	3887.6	2447.9	1522.7	1002.4	867.5	1337.0	2499.4	1658.8	2774.0
144	Luan He	71071.5	174.3	47.2	147.9	279.9	408.1	214.3	964.0	1219.0	725.2	357.8	190.0	115.8	403.6
145	Kura	182283.3	559.4	187.2	708.9	3035.7	4111.4	2768.8	1901.8	1396.6	913.7	766.2	692.7	413.7	1454.7
146	Dalinghe	22823.1	58.3	3.2	6.3	33.3	66.4	80.0	85.0	386.7	210.1	123.7	64.5	39.6	96.4
147	Delaware	26713.4	1640.3	801.3	4062.4	2255.7	1789.8	1043.1	712.5	568.2	587.3	762.0	1406.7	1294.0	1410.3
148	Sacramento	77208.9	5375.8	6127.3	6249.1	5067.8	3136.9	2369.0	2064.4	1708.0	1170.4	511.3	343.5	1439.3	2963.6
149	Huang He (Yellow River)	988062.6	2702.4	608.3	2320.1	5166.0	8177.4	9102.2	10309.3	9805.2	9930.4	5872.9	3052.0	1802.8	5737.4
150	Kizilirmak	77873.6	199.1	836.5	1201.8	2397.1	1578.7	804.4	537.4	393.4	241.0	132.0	83.4	128.5	711.1
151	Yongding He	214406.5	174.7	403.8	1472.8	2529.4	2501.8	1275.4	1729.3	2397.6	1071.8	442.9	185.1	137.8	1193.5
152	Tejo	70351.7	2713.3	2202.2	3261.5	2062.1	1343.3	834.4	721.8	559.4	292.8	124.7	136.5	1182.1	1286.2
153	Sakarya	62482.7	348.5	1016.9	1126.0	888.3	508.1	345.2	273.7	242.4	156.9	68.9	26.5	75.1	423.0
154	Eel (Calif.)	7449.9	1366.1	1258.1	927.1	540.6	304.0	173.7	105.4	63.9	38.7	23.2	15.6	579.5	449.6
155	Tigris & Euphrates	832578.6	15514.6	16609.9	22140.0	25647.8	18735.0	10281.7	7368.3	5738.1	3544.0	2291.3	3575.7	7623.3	11589.1
156	Potomac	32380.6	1355.9	1163.5	1705.2	1374.1	948.5	624.9	370.6	255.1	188.4	213.6	392.6	752.3	778.7
157	Guadiana	66020.0	246.7	715.8	1619.0	1022.7	571.6	571.0	703.0	614.9	301.8	105.4	30.3	15.9	543.2
158	Kitakami	9652.4	690.1	172.5	1357.6	1082.6	802.0	551.9	581.1	590.1	631.9	775.8	828.3	528.7	716.1
159	Mogami	6853.1	969.3	827.6	1039.4	774.9	556.2	376.7	392.9	356.7	430.5	519.9	740.8	1023.9	667.4
160	Han-Gang (Han River)	24771.5	814.3	12.5	1372.3	1838.7	1166.9	1200.5	4312.9	3914.3	2697.0	1388.7	1010.5	540.7	1689.1
161	Guadalquivir	56954.8	677.2	1306.6	3139.9	1977.6	1055.0	1003.9	1110.6	955.3	481.2	185.6	65.2	160.4	1009.9
162	San Joaquin	34365.6	707.1	829.3	1285.7	1335.3	1136.9	1098.6	1267.9	1193.7	818.4	322.2	74.9	102.3	847.7
163	James	23528.4	1600.5	1239.3	1248.0	954.0	670.9	429.1	258.7	169.2	122.7	160.2	381.4	857.5	674.3
164	Bravo	510056.3	263.4	84.8	204.6	657.0	1336.3	937.5	913.3	1079.5	1202.6	640.0	304.7	204.1	652.3
165	Shinano, Chikuma	11158.8	1100.6	807.7	800.2	1837.3	1864.2	1290.9	1242.1	1006.9	1172.1	1174.8	1150.4	1044.7	1207.7
166	Roanoke	26801.0	1844.9	1467.9	1460.0	1085.8	725.1	448.3	316.0	238.3	184.2	159.1	332.5	853.9	759.7
167	Naktong	23325.2	508.2	274.7	755.4	983.6	697.6	959.3	1987.4	1879.2	1673.3	882.4	545.1	344.9	957.6
168	Indus	1139075.4	11918.9	9642.7	18198.4	21870.2	18514.4	18264.8	32379.1	40736.0	31344.3	19300.0	10858.9	6757.3	19982.1
169	Tone	15739.3	936.2	260.8	691.8	1057.7	983.0	973.7	1058.8	1240.9	1561.5	1541.0	979.8	683.0	997.3
170	Salinas	12654.6	11.8	34.5	58.5	36.9	32.6	47.2	66.4	68.2	42.9	9.6	2.7	1.6	34.4
171	Pee Dee	46531.3	2824.5	2381.4	2346.1	1609.2	944.6	589.6	563.3	455.2	460.3	379.9	545.7	1384.2	1207.0
172	Chelif	45249.3	234.5	283.5	281.9	175.3	103.2	88.2	81.1	66.7	44.4	17.2	8.8	39.5	118.7
173	Cape Fear	22652.7	1592.2	1301.8	1225.2	817.3	528.8	353.8	389.9	381.5	348.4	265.8	359.7	725.3	690.8
174	Tenryu	5769.0	584.8	291.0	511.3	839.0	748.4	861.4	816.6	724.3	1026.6	888.4	635.8	438.9	697.2
175	Santee	40035.3	804.8	794.4	752.0	496.5	298.4	193.4	174.2	169.8	140.6	124.1	150.6	395.6	374.5
176	Kiso	5419.1	652.3	218.1	352.4	848.3	836.4	896.9	961.9	750.1	967.8	780.0	608.0	420.8	691.1
177	Yangtze(Chang Jiang)	1745094.4	40746.3	20673.1	48340.7	86583.8	114954.9	136756.4	119291.0	113382.9	101024.6	67829.6	41278.9	25519.8	76365.2
178	Yodo	8424.2	1063.4	536.0	654.4	713.4	583.0	743.8	675.6	510.1	768.0	820.6	652.7	632.7	696.1
179	Sebou	36201.3	1058.8	1081.1	1248.4	934.9	533.2	346.0	284.6	201.0	141.9	76.4	197.1	563.6	555.6
180	Alabama River & Tombigbe	113117.4	9397.8	10770.1	12232.8	8612.8	4829.3	2684.6	1677.7	1055.0	671.1	421.7	465.0	3348.5	4680.5
181	Savannah	27740.4	1501.0	1570.4	1658.6	1058.3	583.9	360.8	272.1	190.7	187.5	165.1	238.5	572.9	696.6
182	Gono (Go)	3949.5	457.3	244.7	265.1	275.8	218.2	354.0	370.2	191.3	273.3	275.9	229.3	269.2	285.4
183	Huai He	174309.9	1271.4	1031.3	2377.3	4348.7	4430.9	4375.0	4755.1	3800.5	3689.6	1900.5	1153.0	823.3	2829.7
184	Apalachicola	51412.9	2889.5	3847.6	4385.4	3016.0	1572.5	912.7	651.6	531.5	402.8	235.8	202.2	1034.8	1640.2
185	Brazos	117853.1	747.2	874.4	717.7	986.3	929.9	587.4	962.4	828.0	508.3	179.4	80.2	367.2	647.4
186	Altamaha	37117.5	1328.3	2248.8	2575.1	1555.3	829.5	474.6	313.9	232.8	182.4	112.6	72.0	424.7	862.5
187	Mekong	787256.9	30683.5	405.8	584.3	939.8	5846.3	34283.6	85153.9	107075.0	107961.6	64294.1	35434.1	20497.8	41096.7
188	Colorado(Caribbean Sea)	110640.3	466.3	688.6	553.1	621.2	522.3	343.0	497.7	455.0	315.6	115.8	35.9	228.7	403.6
189	Trinity(Texas)	46168.2	588.6	822.5	766.2	973.4	839.8	365.7	234.6	152.1	98.0	66.6	52.7	255.9	434.7
190	Pearl	22423.0	1984.2	2012.8	2131.6	1713.4	1094.2	598.0	392.6	256.9	162.9	99.8	148.7	752.9	945.7
191	Sabine	25611.8	1109.9	1334.8	1286.5	1307.3	936.9	456.1	276.7	165.9	101.9	62.1	86.8	295.2	618.3
192	Suwannee	26400.9	1043.0	1262.4	1316.2	797.7	415.7	300.4	613.1	756.9	714.7	385.8	211.7	527.5	695.4
193	Yaqui	76181.7	15.8	30.2	63.2	72.9	36.0	24.2	49.0	61.5	44.6	29.0	14.8	15.8	38.1
194	Nile	3078088.1	20724.2	2310.3	6660.0	16300.5	18779.0	21757.3	48384.2	80541.4	66624.1	36444.6	22470.9	14322.2	29609.9
195	Brahmaputra	518011.4	28402.7	549.4	2782.9	16852.2	46782.8	103699.0	127521.0	128246.8	107215.1	58846.2	31386.4	18647.3	55911.0
196	St.Johns	22489.2	379.1	187.4	226.0	126.3	72.7	67.3	331.1	475.2	796.6	731.2	335.3	225.9	329.5
197	Nueces	43877.9	4.2	8.4	21.4	28.3	35.8	41.5	66.6	49.3	24.4	10.5	5.4	3.8	25.0
198	San Antonio	10952.4	12.3	13.2	14.4	23.4	25.7	19.4	25.1	18.3	9.9	5.7	4.2	8.8	15.0
199	Irrawaddy	411516.3	26908.9	342.4	994.5	4301.3	10976.0	59825.7	98546.8	111837.4	93122.6	59380.0	30238.9	17673.9	42845.7
200	Fuerte	36419.8	340.7	90.6	48.7	39.8	35.5	39.1	166.0	764.0	806.9	406.5	207.8	249.3	266.2
201	Xi Jiang	362894.3	8372.9	2682.6	4932.3	10762.2	25796.8	44596.4	41263.7	42397.2	25138.3	14767.9	8958.4	5447.9	19593.0
202	Bei Jiang	52915.4	629.5	353.9	1921.9	4546.4	6874.5	7013.0	3830.8	3230.7	2159.0	1100.4	668.5	413.5	2728.5

Basin ID	Basin name	Area (km^2)	Natural runoff (Mm^3/month)												
			Jan	Feb	Mar	Apr	May	Jun	Jul	Aug	Sep	Oct	Nov	Dec	Average
203	San Pedro	29358.8	192.9	2.6	4.4	6.2	7.1	3.4	137.0	732.2	987.8	369.0	206.3	130.3	231.6
204	Dong Jiang	32102.9	861.2	105.2	1116.0	3077.8	5758.0	6467.3	4881.8	4699.8	3338.0	1558.5	934.6	567.8	2780.5
205	Mahi	36237.7	884.2	157.9	246.0	223.2	137.5	37.8	2640.9	4426.7	3152.3	1389.2	865.8	573.5	1227.9
206	Damodar	43096.1	1257.9	111.7	160.8	44.8	15.0	253.7	1494.8	4751.2	4657.8	2147.4	1316.0	850.4	1421.8
207	Niger	2117888.7	21778.0	99.0	256.2	1297.8	4833.0	14794.5	38348.0	80634.7	90704.8	47626.3	23829.5	14278.6	28206.7
208	Narmada	95818.2	2407.1	430.8	900.5	1131.7	1138.1	445.1	7352.7	11663.1	8745.2	3757.6	2295.3	1586.4	3487.8
209	Brahmani River (Bhahmani	51973.4	1562.1	33.6	53.2	42.6	38.7	549.7	3888.5	7939.2	6035.6	3059.0	1695.6	1049.7	2162.3
210	Mahanadi(Mahahadi)	135061.1	3751.2	108.6	158.0	151.1	158.2	157.6	5209.0	21461.7	14877.9	6698.9	4119.4	2571.8	4951.9
211	Santiago	126222.3	681.2	116.1	243.6	265.3	168.1	205.1	1027.8	2651.8	3112.7	1413.6	791.4	492.4	930.8
212	Panuco	82929.1	1540.6	95.9	186.5	197.5	137.9	362.8	2013.6	2491.4	6156.6	3524.7	1770.8	1046.5	1627.1
213	Godavari	311698.7	6805.4	626.4	1240.0	1477.3	1571.4	891.7	13461.6	27528.4	26621.8	12095.3	7347.2	4834.4	8708.4
214	Tapti	65096.3	1243.3	168.4	306.4	365.6	395.0	149.1	3365.4	5169.5	5115.9	2124.1	1333.7	886.1	1718.5
215	Sittang	34265.3	2254.3	3.3	6.3	6.6	16.9	3852.3	7852.6	10043.8	8332.3	4900.8	2492.4	1478.4	3436.7
216	Armeria	9639.1	60.6	8.1	17.0	28.8	27.6	10.1	3.3	40.7	292.2	147.3	71.6	48.0	62.9
217	Ca	28747.0	1447.9	56.1	26.8	23.3	154.3	584.1	1876.9	2678.4	4330.1	2767.9	1652.1	965.9	1380.3
218	Chao Phraya	188419.1	4183.6	318.1	519.8	537.6	493.6	1640.2	5541.8	9607.1	16009.4	10072.0	5811.4	3059.8	4816.2
219	Krishna	269869.0	4249.9	610.7	1255.2	1355.6	1400.2	3256.5	16600.9	15185.1	11795.9	6710.3	4670.2	3427.8	5876.5
220	Senegal	436981.1	1629.1	10.0	15.2	11.7	12.0	447.8	3062.5	8464.3	6863.4	3270.9	1792.3	1076.7	2221.3
221	Papaloapan	39885.1	1877.2	12.4	16.0	15.8	11.5	477.3	2373.0	4411.2	5780.4	4426.4	2194.2	1261.7	1904.8
222	Grisalva	127675.5	11859.4	1204.2	664.4	610.2	1646.9	8622.6	11809.7	13230.5	20505.5	19300.8	10998.3	7877.1	9027.5
223	Verde	18342.8	378.7	2.9	6.5	6.9	4.5	10.1	365.4	850.4	1648.0	893.8	414.1	250.8	402.7
224	Mae Klong	28004.2	1554.4	20.9	34.2	33.5	1076.4	3580.0	5254.6	5831.8	5582.5	3551.6	1715.1	1031.1	2438.9
225	Tranh (Nr Thu Bon)	9459.9	2007.0	45.2	24.6	16.4	71.3	290.8	883.2	1278.8	1770.6	2724.1	2418.9	1615.2	1095.5
226	Penner	54976.4	568.7	34.4	52.1	45.1	43.8	41.5	158.5	151.2	182.9	360.5	1017.5	485.3	261.8
227	Volta	414004.1	2522.1	7.7	80.1	291.8	828.4	2616.6	3570.8	8402.5	11296.3	5427.0	2744.7	1654.9	3286.9
228	Lempa	18088.5	888.1	2.9	7.6	10.3	11.7	547.6	1582.1	1968.2	3042.2	2295.6	976.3	585.2	993.2
229	Gambia	69874.3	750.8	0.3	0.3	0.3	0.3	145.1	922.2	3078.2	3466.5	1537.8	816.1	492.4	934.2
230	Grande De Matagalpa	17991.9	1788.5	87.6	46.4	30.2	37.4	1350.9	2694.3	2443.9	2701.8	2950.2	1748.2	1248.2	1427.3
231	Cauvery	91159.4	2091.4	159.7	385.4	347.3	350.9	1080.8	3669.7	3305.4	2574.1	2347.6	2777.6	1849.0	1744.9
232	San Juan	41659.4	5223.3	533.6	282.7	261.5	1036.0	3952.8	4778.7	4793.9	6119.6	7350.0	4839.9	3921.9	3591.2
233	Geba	12774.4	537.0	3.5	4.3	4.3	3.5	79.2	413.4	1814.0	2305.1	1207.1	582.6	353.4	608.9
234	Corubal	24258.0	882.9	0.4	0.5	0.5	0.4	293.0	1767.6	3594.2	3165.9	2080.6	964.3	579.0	1110.8
235	Magdalena	261204.9	27118.0	3452.3	6430.2	14175.3	21211.1	18633.1	15055.6	15479.8	18291.2	31846.5	31789.4	20916.3	18699.9
236	Comoe	78506.9	447.9	3.4	4.6	105.1	306.0	923.2	676.9	1018.0	1509.8	1021.9	540.3	296.4	571.1
237	Orinoco	952173.4	73559.9	9908.6	16110.2	48197.5	96502.6	137370.7	156922.8	139130.4	112036.3	103445.6	77389.5	46830.9	84783.7
238	Bandama	98751.1	1337.1	4.0	5.7	118.4	306.8	1520.8	1055.3	2949.0	5574.4	3186.6	1473.0	881.5	1534.4
239	Oueme	59842.6	458.8	1.0	1.1	6.9	240.8	976.7	1265.1	1320.9	1893.3	1037.9	499.0	301.2	666.9
240	Sassandra	68097.5	2261.5	1.4	3.2	129.1	309.3	2250.3	3322.8	4638.5	8322.9	5416.1	2603.4	1487.3	2562.2
241	Shebelle	805077.0	1126.2	49.5	54.4	2532.2	1755.0	1025.3	1594.0	2005.1	1944.7	1681.9	1610.5	791.5	1347.5
242	Mono	23899.0	122.1	0.3	14.5	50.7	126.1	330.7	356.9	319.3	472.0	289.3	132.5	80.1	191.2
243	Congo	3698918.1	193908.5	92837.8	126684.3	138968.9	93475.1	62522.2	55481.6	71460.1	90395.6	111123.4	108901.0	123157.3	105743.0
244	Atrato	34619.5	8908.3	2297.1	2736.4	4317.7	5624.4	5876.9	5976.7	6096.9	6689.5	7140.9	7032.0	5537.1	5686.2
245	Cuyuni	85635.0	9798.1	3136.9	2829.9	4268.1	9871.7	13477.9	13021.6	10134.4	5445.8	3587.1	3665.6	6571.4	7150.7
246	Cavally	30665.2	2295.7	105.8	221.1	532.9	1553.0	3418.3	2447.3	1941.8	4204.8	4188.2	2935.7	1654.9	2125.0
247	Tano	15656.1	321.4	0.2	32.5	186.6	547.1	1356.6	700.0	337.1	480.9	810.9	421.8	218.4	451.1
248	Cross	52820.2	3986.4	1.4	608.2	1184.8	2346.5	4731.8	7828.2	9133.6	11624.3	10815.8	4452.2	2614.4	4944.0
249	Sanaga	134252.0	4812.3	3.0	236.5	2004.2	4068.5	5840.0	7929.0	9616.8	13725.9	13358.0	5383.1	3156.1	5844.5
250	Pra	23479.8	378.6	3.2	102.6	282.2	657.8	1316.6	691.0	327.9	645.8	1039.5	483.0	247.6	514.6
251	Davo	8460.3	133.7	0.2	0.2	2.0	9.6	542.8	283.0	126.7	238.1	288.6	192.8	89.5	158.9
252	Essequibo	68788.3	5069.1	1976.6	2110.6	2932.1	7163.7	13057.2	11720.0	7957.4	3936.3	2438.8	1874.9	3105.6	5278.5
253	Kelantan	14419.9	3574.8	439.2	342.5	417.5	434.1	481.7	494.1	561.2	1352.9	2084.8	2431.3	2719.0	1277.8
254	Corantijn	65527.6	1313.6	829.3	1710.9	3554.5	11297.6	13180.8	9711.4	6084.0	3012.4	1811.6	1094.1	692.7	4524.4
255	Coppename	24750.2	1551.1	1394.4	1540.4	2071.4	4023.6	4578.3	3861.0	2331.4	1160.6	695.4	420.0	321.1	1995.7
256	Kinabatangan	14101.7	2820.1	862.7	675.4	672.8	675.3	1148.9	787.8	1142.6	1468.5	1388.6	1249.3	1909.0	1233.4
257	Maroni	65944.9	3849.5	4583.6	5326.3	7656.9	11080.5	10145.5	7092.8	4342.7	2242.1	1349.5	815.1	565.6	4920.8
258	San Juan (Columbia - Paci	13898.0	6064.5	2203.5	2616.1	3456.3	4124.0	3912.7	3904.9	3971.2	4103.4	4529.7	4529.1	3905.9	3943.4
259	Amazonas	5880854.9	950375.6	705085.8	813922.6	857876.6	713184.6	564388.1	424106.1	299107.1	238076.7	243680.0	298076.1	455711.2	546965.9
260	Pahang	28436.7	5776.0	1292.5	1416.5	1962.1	1937.4	1260.3	863.4	823.6	1395.6	2714.8	3612.5	4175.9	2269.2
261	Nyong	34626.2	1269.1	0.1	386.2	1153.1	1817.1	1504.5	698.1	677.8	2434.6	3389.9	1699.9	832.3	1321.9
262	Oyapock	27075.7	4282.0	4043.5	4817.3	6249.8	6602.9	5755.6	3526.6	2069.9	1131.7	683.3	412.7	586.7	3346.8
263	Rajang	49943.5	20497.2	8513.0	9657.7	10211.2	9935.6	7769.6	6882.1	6855.9	9242.6	11129.0	11855.9	12276.9	10402.2
264	Ntem	33526.9	2055.6	8.3	442.8	1623.3	2595.7	1808.1	791.1	470.6	1558.6	4430.8	3192.8	1428.3	1700.5
265	Ogooue	222662.7	22726.7	7569.4	15733.8	20776.5	19147.2	7950.2	4618.0	2791.3	2505.1	8651.3	23592.1	17443.6	12792.1
266	Rio Araguari	33771.5	4727.0	5377.5	6935.9	8461.5	8118.0	7123.8	4272.9	2486.6	1376.3	831.0	501.9	398.5	4217.6
267	Mira	13264.8	2073.0	1259.4	1291.0	1430.0	2032.3	1959.3	1248.7	1229.1	1355.3	1165.3	1283.2	912.8	1436.6
268	Esmeraldas	19796.2	2922.0	4238.0	5676.0	6743.2	4668.6	2504.1	1398.8	876.1	589.2	579.1	755.5	966.5	2659.8
269	Tana	95715.0	290.5	14.7	38.9	512.9	706.1	333.2	183.6	113.4	69.9	103.6	260.5	305.1	244.4
270	Daule & Vinces	41993.5	2730.9	4080.6	5260.2	4696.3	2419.3	1482.0	915.4	644.8	472.3	447.2	458.1	400.9	2000.7
271	Rio Gurupi	32335.3	491.7	2048.7	4647.2	4513.5	3426.0	2151.5	1412.8	764.6	450.8	272.3	164.5	103.1	1703.9
272	Rio Capim	54888.3	1537.9	5606.4	8799.8	7926.1	5924.9	3782.3	2616.7	1569.4	878.6	525.8	317.7	205.5	3307.6
273	Tocantins	774718.3	82937.0	65826.8	71926.3	45721.6	24110.4	14567.5	8930.8	5585.4	3923.1	3979.0	16536.2	44543.4	32382.3
274	Kouilou	60000.0	4007.3	2400.3	3950.9	5306.6	3068.4	1426.7	862.7	522.5	316.7	191.7	1130.5	3001.1	2182.1
275	Nyanga	12369.1	1159.8	691.5	941.1	1044.4	523.6	267.4	161.5	97.5	58.9	35.6	692.8	805.1	539.9
276	Rio Parnaiba	336584.2	2039.6	3746.9	7333.0	6991.8	3124.0	1723.6	1047.6	641.5	394.0	242.4	157.1	558.8	2333.4
277	Rio Itapecuru	52672.0	84.3	1224.8	3260.0	3253.3	1651.3	883.6	520.8	314.3	190.4	115.4	69.7	42.3	967.5
278	Rio Acarau	14472.9	22.3	31.8	810.4	1076.9	646.7	301.3	179.6	110.0	67.9	42.1	26.2	16.4	277.6
279	Pangani	50364.8	34.7	22.7	34.9	203.1	493.3	239.9	152.6	87.1	59.8	38.7	28.7	27.5	118.6
280	Rio Pindare	39112.0	253.4	2304.7	4716.9	4525.5	2674.7	1415.4	814.8	488.9	295.4	178.6	108.0	65.3	1486.8
281	Sepik	81119.7	15375.9	9555.7	12665.7	12180.9	9397.5	7423.9	6795.9	6812.5	7707.7	8100.4	8084.8	9182.5	9440.3
282	Rio Mearim	56687.0	356.4	2713.7	5445.6	4657.5	2321.9	1253.1	745.7	450.8	272.8	165.1	99.9	61.7	1545.3
283	Chira	16699.6	76.0	208.7	379.5	341.5	138.2	86.6	62.2	51.9	37.8	21.8	23.1	17.1	120.4
284	Rufiji	204638.8	1836.2	3827.1	7683.8	8804.4	4027.1	2129.0	1285.4	779.2	473.6	288.1	176.3	418.8	2644.1
285	Rio Jaguaribe	72804.3	64.1	11.7	1619.3	2744.3	1562.6	861.9	515.6	319.8	213.3	142.0	92.4	60.4	683.9
286	Purari	32139.9	7089.0	4200.2	5204.7	5335.2	4459.8	3535.0	2952.7	3064.9	3853.1	3615.9	3635.9	4215.8	4263.5
287	Ruvu	17541.2	121.7	122.6	361.9	952.4	722.0	287.6	174.0	106.2	64.9	39.6	23.9	73.4	254.2
288	Rio Paraiba	18969.1	37.7	2.0	3.5	89.5	253.8	514.2	396.9	208.3	115.4	73.2	46.6	29.6	147.6
289	Solo (Bengawan Solo)	15146.1	2855.5	2415.3	2433.6	1722.1	949.3	531.7	332.1	222.4	171.0	106.2	250.0	1099.8	1090.8
290	Sao Francisco	628629.1	27716.3	15251.7	13981.8	7511.4	4656.8	5028.4	4947.7	3343.7	1665.4	1268.7	4929.8	18730.9	9086.0
291	Brantas	10822.6	1795.8	1741.1	1762.4	1247.5	696.8	395.2	243.3	163.6	122.2	71.2	160.7	531.2	744.2
292	Santa	11882.5	455.4	563.1	650.9	399.4	210.8	125.2	78.5	57.1	44.0	79.5	128.6	132.8	243.8
293	Zambezi	1388572.2	73279.9	82019.0	68514.8	32327.8	18268.1	10936.3	6663.6	4203.7	2618.2	1621.8	1305.4	21500.8	26938.3
294	Rio Vaza-Barris	15314.2	17.1	1.4	1.7	1.6	61.4	151.1	177.6	102.7	49.8	30.8	18.8	11.8	52.2
295	Rio Itapicuru	37593.4	56.5	2.3	2.1	7.2	99.2	263.0	645.3	362.8	169.7	102.3	62.2	38.4	150.9
296	Rio Paraguacu	54607.1	274.1	185.7	300.7	705.2	1494.5	1165.9	1466.7	878.0	455.0	267.7	194.3	163.9	629.3
297	Canete	5755.2	257.6	233.3	231.1	128.4	67.0	41.2	25.4	17.5	12.9	43.0	62.3	113.9	102.8
298	Rio De Contas	56526.5	297.0	120.1	258.0	556.9	550.1	447.9	457.9	292.5	179.2	110.9	165.1	224.8	305.0
299	Roper	79907.5	434.9	1537.1	1682.2	599.2	358.4	216.7	131.2	79.7	48.5	29.6	17.7	10.5	428.8
300	Daly	53414.6	783.7	2684.1	2482.2	919.0	552.0	333.8	202.0	122.6	74.6	45.3	27.1	16.9	686.9
301	Drysdale	26015.9	26.2	283.6	270.5	96.7	58.3	35.2	21.3	12.8	7.8	4.7	2.8	1.7	68.5
302	Parana	2640486.1	105979.7	72853.1	68346.4	50136.2	39988.6	34546.3	22504.9	18037.0	19546.7	24410.8	32882.5	57492.3	45560.4
303	Durack	29363.2	0.0	1.9	1.6	0.6	0.4	0.2	0.1	0.1	0.1	0.0	0.0	0.0	0.4
304	Rio Prado	31673.7	696.0	283.3	357.3	417.6	243.4	187.0	168.8	106.4	58.7	36.6	137.5	549.1	270.1
305	Victoria	78462.4	0.1	20.4	14.0	5.5	3.3	2.0	1.2	0.7	0.4	0.3	0.2	0.1	4.0

Basin ID	Basin name	Area (km^2)	Natural runoff (Mm^3/month)												
			Jan	Feb	Mar	Apr	May	Jun	Jul	Aug	Sep	Oct	Nov	Dec	Average
306	Mitchell(N. Au)	71725.2	1080.2	6217.0	5876.8	2616.6	1435.1	859.9	521.2	317.3	194.5	119.8	72.3	43.0	1612.8
307	Majes	18612.1	893.9	994.8	875.9	436.0	232.5	139.9	84.7	52.7	34.4	31.7	29.3	363.0	347.4
308	Ord	55686.1	0.0	3.9	1.5	2.7	4.2	5.2	6.4	7.5	8.1	6.6	3.6	0.2	4.2
309	Jequitinhonha	68548.9	4207.8	1648.8	1430.8	860.3	471.5	297.0	203.6	126.2	74.1	60.4	675.7	3152.4	1100.7
310	Macarthur	19673.6	0.4	1.1	55.6	14.5	8.8	5.3	3.2	1.9	1.2	0.7	0.4	0.3	7.8
311	Fitzroy	94043.9	5.5	446.6	490.3	167.3	101.0	61.0	36.9	22.3	13.5	8.1	4.9	3.0	113.4
312	Gilbert	46429.1	183.1	1374.9	1126.1	428.1	256.1	154.7	93.5	56.5	34.2	20.8	12.5	7.6	312.4
313	Mucuri	16732.2	1331.8	412.5	315.0	254.2	167.2	113.9	88.6	50.0	28.9	21.6	212.3	1046.2	336.8
314	Rio Doce	86085.9	12563.7	5298.4	4242.2	2461.5	1301.4	784.3	483.1	302.0	187.0	117.8	2334.5	9099.0	3264.6
315	Save	114957.8	2203.3	3356.1	2440.7	1065.6	627.7	386.7	242.8	173.8	130.9	85.0	41.4	348.7	925.2
316	Burdekin	130426.5	690.0	3679.3	3662.4	1887.1	1001.1	597.8	363.8	227.1	146.3	95.8	59.5	32.7	1036.9
317	Tsiribihina	61991.9	9631.8	9804.7	9049.7	4154.5	2354.3	1436.8	893.2	545.1	328.9	198.3	299.2	2841.4	3461.5
318	Buzi	27904.7	1304.7	1917.2	1888.8	752.6	437.8	265.0	160.8	98.8	61.6	38.5	22.6	110.9	588.3
319	Loa	50206.4	0.3	0.4	0.3	0.3	0.3	0.3	0.3	0.3	0.3	0.3	0.3	0.3	0.3
320	Limpopo	415623.1	1880.1	3058.9	2803.2	1433.2	767.0	501.7	359.2	334.0	330.6	246.8	159.6	308.4	1015.2
321	De Grey	56818.6	0.0	0.0	0.0	0.0	0.0	0.0	0.0	0.0	0.0	0.0	0.0	0.0	0.0
322	Paraiba Do Sul	58027.2	7384.0	4105.7	3823.9	2180.1	1239.1	759.3	470.0	308.5	264.6	590.0	1633.4	4400.3	2263.2
323	Fortescue	49924.5	0.0	0.0	0.0	0.0	0.0	0.0	0.0	0.0	0.0	0.0	0.0	0.0	0.0
324	Mangoky	43141.1	1857.9	2203.9	1852.5	898.9	518.2	330.4	220.7	136.7	83.0	50.1	56.6	333.7	711.9
325	Fitzroy	142915.3	72.4	1909.6	2134.9	885.3	493.9	300.8	192.9	132.5	101.8	79.8	54.4	35.2	532.8
326	Orange	972388.4	1857.2	2095.4	2246.2	1402.3	807.0	489.5	342.5	319.8	311.7	362.3	534.8	884.1	971.1
327	Ashburton	75842.1	0.0	0.0	0.0	0.0	0.0	0.0	0.0	0.0	0.0	0.0	0.0	0.0	0.0
328	Gascoyne	75998.4	0.0	0.0	0.0	0.0	0.0	0.0	0.0	0.0	0.0	0.0	0.0	0.0	0.0
329	Rio Ribeira Do Iguape	25697.5	2174.8	1657.5	1425.9	887.3	735.4	782.1	538.1	442.8	601.1	860.6	773.8	987.3	988.9
330	Incomati	46295.7	1039.9	1118.5	1027.4	517.1	276.9	173.5	113.5	83.8	67.7	43.3	129.4	456.6	420.6
331	Murray	1059507.7	2868.3	1379.7	1501.2	1110.5	1279.6	2153.4	2511.6	3164.9	3371.2	3337.6	2314.5	2000.4	2249.4
332	Murchison	91416.1	0.0	0.0	0.0	0.0	0.0	0.0	0.0	0.0	0.0	0.0	0.0	0.0	0.0
333	Maputo	30937.8	924.9	719.0	618.4	324.5	179.7	114.6	75.2	58.1	46.2	33.2	112.3	467.1	306.1
334	Uruguay	265504.6	15702.5	5633.7	8112.2	13990.4	16949.4	19158.1	16344.5	15620.3	18876.7	20160.7	12864.3	9587.2	14416.7
335	Tugela	30079.3	758.7	786.8	752.8	372.3	204.5	126.4	86.3	74.3	66.9	64.9	86.0	376.1	313.0
336	Colorado (Argentinia)	390631.1	3500.8	346.2	220.9	135.4	373.1	707.2	841.7	929.8	908.5	2371.9	3152.6	2575.4	1338.6
337	Rio Jacui	70798.0	5005.1	2492.1	2901.2	3920.9	4897.0	5793.0	5339.7	5145.1	5743.1	5136.4	3405.9	2794.2	4381.1
338	Huasco	9871.6	92.1	16.5	10.1	6.1	3.7	2.4	1.6	1.4	1.6	1.5	0.6	46.0	15.3
339	Limari	11780.3	118.1	101.5	40.8	21.1	12.6	27.9	18.7	17.6	14.6	15.5	11.4	31.8	36.0
340	Negro (Uruguay)	70756.4	1301.6	86.9	492.1	1499.0	2274.1	3311.9	3222.8	3290.1	3379.1	2823.2	1553.8	846.5	2006.8
341	Groot-Vis	30441.2	10.7	24.1	23.0	13.9	11.7	9.5	9.9	13.2	21.7	27.3	18.9	18.9	16.9
342	Salado	266263.9	1011.5	24.7	73.2	787.5	1228.1	1234.8	1105.6	959.3	1261.2	1567.9	1418.0	767.6	953.3
343	Blackwood	22584.8	79.6	0.6	0.7	0.4	0.1	29.9	254.5	407.6	301.6	166.9	86.8	52.5	115.1
344	Rapel	15689.5	1119.4	178.1	113.7	66.3	510.5	1373.7	1356.5	1185.4	876.8	701.4	412.7	681.5	714.7
345	Negro (Argentinia)	130062.1	2461.3	98.8	347.1	1009.8	4025.3	5859.2	6210.5	6075.1	5030.4	4368.0	3162.3	1740.6	3365.7
346	Biobio	24108.6	1512.9	29.1	298.7	919.1	3736.5	4786.4	5042.5	4670.4	4131.1	2973.2	1809.7	1045.2	2579.6
347	Waikato	15358.7	1209.9	436.0	382.1	601.3	1271.4	1642.5	1617.1	1551.8	1388.8	1356.2	1080.3	781.6	1109.9
348	South Esk	10842.5	186.5	8.9	10.5	32.6	76.3	208.6	392.9	471.5	403.2	358.1	217.0	135.7	208.5
349	Chubut	145351.9	837.4	70.0	172.1	336.2	1273.9	2263.3	2596.8	3116.1	2246.4	1454.2	895.8	580.3	1320.2
350	Clutha	17118.9	1029.1	421.2	482.8	693.8	684.7	694.4	642.0	723.7	898.4	957.1	762.4	659.5	720.8
351	Baker	30760.3	1928.9	630.2	1099.0	1638.6	2259.5	2536.2	2753.8	2648.6	2149.0	1882.5	1579.4	1282.9	1865.7
352	Santa Cruz	30599.9	1652.2	385.0	560.0	1181.8	1590.7	1850.7	1577.2	2464.8	3221.0	3226.9	1605.8	1042.5	1696.6
353	Ganges	1024462.6	32182.1	10981.6	16447.4	12896.7	12922.0	27823.6	78624.5	128519.9	96972.9	47842.1	32621.7	19626.1	43121.7
354	Salween	258475.2	8366.0	95.5	592.3	1511.4	2846.6	12055.4	24649.1	32068.1	27586.6	18159.4	9737.1	5522.3	11932.5
355	Hong(Red River)	157656.9	4779.9	80.7	104.5	254.5	1566.3	7439.6	18447.1	22644.5	16383.8	9602.6	5433.7	3149.0	7490.5
356	Lake Chad	2391218.9	6870.6	135.4	144.7	180.2	263.0	1227.0	7989.1	36416.6	27951.2	14050.2	7408.8	4490.9	8927.3
357	Okavango	705055.7	4075.2	6488.8	8619.1	3971.7	2041.0	1233.1	745.9	452.1	274.8	167.1	100.7	882.0	2421.0
358	Tarim	1051731.4	241.9	77.0	269.6	593.6	1657.0	2845.3	3326.7	2360.3	1351.4	540.9	295.2	164.1	1143.6
359	Horton	23926.2	12.4	0.3	0.2	0.1	78.8	314.9	93.6	54.9	33.2	20.0	12.1	7.3	52.3
360	Hornaday	14778.0	12.4	0.0	0.0	0.0	0.0	181.3	145.7	70.2	37.1	22.3	13.5	8.1	40.9
361	Conception	25569.5	0.6	1.8	3.6	4.8	3.8	4.7	4.3	5.9	5.5	4.2	1.5	1.1	3.5
362	Ulua	26392.0	1735.8	77.7	42.4	31.4	19.3	510.5	1791.6	1997.4	3135.9	2748.5	1916.8	1262.0	1272.4
363	Patacua	24232.4	1710.9	118.6	53.8	32.7	19.7	70.9	693.4	892.9	1438.3	2176.2	1802.4	1293.7	858.6
364	Coco	25502.0	2767.8	143.3	70.7	43.3	48.4	1698.7	3244.2	2959.2	3280.5	3939.1	2788.7	2041.2	1918.8
365	Ocona	16063.9	539.4	582.1	517.1	245.1	134.9	80.9	48.8	30.6	19.9	49.2	71.2	235.0	212.9
366	Cuanza	141391.1	10360.7	7565.1	10296.0	8746.9	3552.8	2127.7	1286.3	779.0	472.4	307.3	304.1	5458.5	4271.4
367	Cunene	110545.5	1828.9	2579.9	5240.5	2925.0	1322.8	798.9	482.7	291.8	176.4	112.8	98.1	642.6	1375.0
368	Doring	48855.5	44.0	20.3	25.5	14.8	4.2	92.2	148.6	176.0	136.2	105.1	61.5	43.1	72.6
369	Gamka	45676.2	65.3	14.4	28.8	40.5	41.1	51.1	45.0	62.9	111.5	105.9	87.0	55.8	59.1
370	Groot- Kei	18678.3	2.7	6.5	16.9	12.9	8.5	5.9	5.6	6.2	7.9	8.3	6.0	4.4	7.6
371	Lurio	61172.2	4604.7	6185.0	6089.2	2522.2	1435.2	866.8	523.5	316.3	191.1	115.4	69.7	468.0	1948.9
372	Messalo	24810.9	941.3	1818.6	2197.0	1098.8	548.0	330.8	199.8	120.7	72.9	44.1	26.6	16.1	617.9
373	Rovuma	151948.6	9106.2	15265.1	18092.1	9319.0	4605.3	2779.5	1678.9	1014.2	612.7	370.2	223.6	516.4	5298.6
374	Galana	51921.7	187.2	7.8	34.8	597.5	654.3	327.5	177.5	104.7	64.5	40.0	203.0	196.1	216.2
375	Pyasina	63888.8	470.7	4.9	3.1	2.0	1.3	8582.4	2828.9	1874.0	1803.8	814.2	491.9	297.2	1431.2
376	Popigay	48954.2	84.9	0.8	0.5	0.3	0.2	2052.3	754.1	410.5	251.2	146.9	88.7	53.6	320.3
377	Fuchun Jiang	37697.9	1253.4	1966.9	3249.4	3037.5	4025.8	5573.0	2420.0	1437.2	1282.3	886.7	707.7	572.7	2201.1
378	Min Jiang	60039.7	1710.0	2261.2	6444.7	6263.4	9721.2	10643.9	4850.2	3725.5	2882.6	2049.4	1340.9	880.7	4397.8
379	Han Jiang	30741.5	429.5	189.6	1241.1	2010.5	3792.4	4718.0	2447.5	2125.6	1654.9	782.8	466.3	283.9	1678.5
380	Mamberamo	75416.0	15446.4	9453.9	12610.4	11467.8	9723.6	7842.7	8385.4	7964.6	8697.3	6547.6	7173.3	8630.9	9495.3
381	Lorentz	4299.3	493.1	445.0	515.7	475.1	346.5	253.8	280.8	253.8	345.8	199.3	277.0	262.3	345.7
382	Eilanden	20077.5	5431.2	3334.4	3835.7	3751.0	3567.3	3244.7	3235.3	3090.1	3371.3	2701.3	2824.9	3278.9	3472.2
383	Uwimbu	29373.9	8952.7	5395.2	6379.0	5992.2	6010.0	5432.3	5263.6	5126.4	5387.6	4552.8	4357.4	5401.7	5687.6
384	Sungai Kajan	33171.8	10769.4	4092.4	5488.6	6619.3	6870.7	5795.5	5104.8	4917.8	6431.1	6999.8	7819.9	6829.4	6478.2
385	Sungai Mahakam	75822.7	18606.0	7876.5	10134.8	13756.5	12600.1	9668.4	6831.4	5649.9	5951.6	7402.7	11152.8	12616.4	10187.3
386	Sungai Kapuas	84902.3	29491.3	13356.0	15227.7	15516.0	13513.3	10291.5	7393.4	6608.1	8936.3	13775.6	17293.6	17622.4	14085.4
387	Batang Kuantan	16739.0	3820.3	1460.7	1731.9	2316.9	1825.4	1075.7	640.6	525.6	838.5	1724.9	2579.6	2602.7	1761.9
388	Batang Hari	42872.4	10918.4	4536.0	5450.1	6252.7	4790.1	2820.9	1767.2	1540.6	2438.0	4411.4	6272.8	7176.5	4864.6
389	Flinders	110041.3	0.4	89.8	28.2	15.5	9.4	5.7	3.5	2.2	1.4	0.9	0.6	0.4	13.2
390	Leichhardt	33399.2	31.9	34.8	11.9	7.2	4.4	2.6	1.6	1.0	0.6	0.4	0.2	0.1	8.1
391	Escaut (Schelde)	21498.7	1351.5	705.9	604.0	472.4	284.9	168.8	111.6	79.8	54.8	63.7	373.3	746.1	418.1
392	Issyk-Kul	191032.5	300.5	48.6	2132.1	3385.1	4295.9	3990.2	3002.3	1671.5	1072.0	585.2	384.5	205.0	1756.1
393	Balkhash	423657.4	273.5	5.8	2199.0	4046.6	4089.9	2907.0	2102.5	1368.5	905.0	412.3	445.2	181.7	1578.1
394	Eyre Lake	1188841.3	0.2	0.3	0.6	0.5	0.5	0.4	0.4	0.6	0.7	0.7	0.5	0.4	0.5
395	Lake Mar Chiquita	154330.1	490.2	594.7	1026.2	504.2	261.0	162.6	112.7	87.1	80.9	85.5	95.2	137.7	303.2
396	Lake Turkana	181536.0	2082.5	9.0	100.4	1988.3	3300.7	4415.2	6718.5	7827.8	6549.2	4135.2	2501.6	1394.5	3418.6
397	Dead Sea	35444.0	605.0	681.7	454.0	289.3	257.0	203.5	196.8	168.4	96.6	58.6	31.9	117.9	263.4
398	Suriname	24867.7	2186.8	1915.0	2088.7	2922.6	4729.6	5013.8	3973.9	2420.7	1213.0	729.1	440.4	526.6	2346.7
399	Lake Titicaca	107215.3	7124.5	5963.3	4974.8	2951.8	1893.3	1052.7	658.3	552.1	469.3	688.1	678.7	2594.8	2466.8
400	Lake Vattern	11336.5	109.1	0.5	645.9	535.8	246.0	136.0	81.3	60.0	40.2	82.5	196.1	85.7	184.9
401	Great Salt Lake	74114.4	65.3	237.4	354.3	782.1	915.8	634.2	481.7	350.5	213.3	121.5	68.5	66.7	357.6
402	Lake Taymur	138782.9	723.7	0.0	0.0	0.0	0.0	14735.9	6387.8	3298.4	2513.9	1300.5	785.5	474.4	2518.3
403	Daryacheh-Ye Orumieh	30335.7	156.1	103.1	506.3	1527.4	1539.9	737.8	467.8	347.1	213.8	126.4	137.4	116.8	498.3
404	Van Golu	17736.8	117.9	0.6	127.0	1118.2	1378.3	489.2	294.9	184.2	112.7	115.2	230.6	77.6	353.9
405	Ozero Sevan	4765.3	8.6	0.7	3.8	76.9	106.9	55.9	31.2	21.0	12.9	13.1	13.9	5.9	29.2
