# Supplementary material for: Global Monthly Water Scarcity: Blue Water Footprints versus Blue Water Availability
Source: PLoS One. 2012 Feb 29;7(2):e32688. doi: 10.1371/journal.pone.0032688 (PMC3290560; doi:10.1371/journal.pone.0032688)
Supplement: Table S3 — Monthly blue water availability for the world's major river basins. (PDF) [file pone.0032688.s007.pdf]

Table S3. Monthly blue water availability for the world's major river basins

Basin ID	Basin name	Blue water availability (Mm^3/month)												
		Jan	Feb	Mar	Apr	May	Jun	Jul	Aug	Sep	Oct	Nov	Dec	Average
1	Khatanga	314.3	11.7	7.1	4.3	44.2	5079.7	2577.0	1389.0	871.4	483.8	292.2	176.5	937.6
2	Olenek	246.2	32.0	19.3	11.7	131.7	3286.4	1102.1	614.1	374.1	221.3	133.7	80.7	521.1
3	Anabar	105.2	17.1	10.3	6.2	3.8	825.6	416.5	202.3	120.5	71.0	42.9	25.9	153.9
4	Yana	179.2	14.6	8.8	5.3	31.8	1507.2	1364.1	761.5	366.5	221.4	133.7	80.8	389.6
5	Yenisei	3227.0	148.4	90.5	1550.5	32543.0	32409.6	19438.0	12848.2	9618.3	4888.2	2891.2	1747.0	10116.7
6	Indigirka	380.5	35.9	21.7	13.1	90.2	3362.0	2901.5	1357.2	726.2	435.2	262.9	158.8	812.1
7	Lena	3154.4	131.0	79.3	72.4	17418.4	24981.6	16930.1	12609.2	10727.2	4767.9	2878.1	1738.4	7957.3
8	Omoloy	5.4	0.1	0.1	0.0	0.0	85.2	55.4	25.8	14.7	8.7	5.3	3.2	17.0
9	Tana (NO, FI)	14.4	0.0	0.0	0.0	570.3	155.7	91.4	55.2	43.5	29.8	15.6	9.4	82.1
10	Colville	37.2	0.3	0.2	0.1	9.1	387.6	395.5	206.9	115.8	64.9	39.2	23.7	106.7
11	Alazeya	36.8	6.2	3.8	2.3	1.4	379.4	111.0	62.8	38.0	22.9	13.8	8.4	57.2
12	Anderson	10.9	0.1	0.0	0.0	509.7	159.5	87.0	52.5	31.7	19.2	11.6	7.0	74.1
13	Kolyma	744.2	32.2	19.4	11.8	1068.5	5088.2	7188.3	3241.6	2103.5	1115.2	673.6	406.8	1807.8
14	Tuloma	18.9	2.3	1.4	0.9	346.4	109.5	60.0	36.1	23.8	24.3	11.0	6.7	53.4
15	Muonio	28.8	2.6	1.6	42.8	401.1	222.1	150.0	77.7	59.0	37.5	20.3	12.3	88.0
16	Yukon	970.1	50.5	30.5	188.7	10633.3	9753.4	5955.3	3321.4	2581.2	1529.6	842.4	508.9	3030.4
17	Palyavaam	21.3	0.0	0.0	0.0	0.0	375.7	207.3	105.3	71.0	38.2	23.1	13.9	71.3
18	Kemijoki	97.4	0.6	0.4	118.8	1797.4	489.6	291.6	186.9	216.4	273.3	103.3	62.4	303.2
19	Mackenzie	1127.6	17.4	10.7	1812.7	15880.7	15834.5	9200.4	4975.5	3146.6	2089.5	1153.4	696.8	4662.1
20	Noatak	31.3	0.2	0.1	0.1	80.4	396.1	219.9	124.9	125.9	55.1	33.3	20.1	90.6
21	Anadyr	236.4	0.3	0.2	0.1	490.4	4399.7	2076.1	1092.3	821.1	423.2	255.6	154.4	829.2
22	Pechora	491.8	6.2	3.8	289.8	11661.2	7759.0	3254.0	1926.2	1571.1	890.0	508.6	307.2	2389.1
23	Lule	62.6	0.2	0.1	223.8	770.3	756.9	379.1	222.4	190.3	136.6	67.2	40.6	237.5
24	Kalixaelven	15.7	1.3	0.8	77.7	247.6	151.5	70.3	40.6	27.5	23.7	11.5	7.0	56.3
25	Ob	1386.0	39.6	27.2	16168.4	29723.9	13645.7	7368.0	4708.1	3655.1	2815.0	1373.0	832.4	6811.9
26	Ellice	6.1	0.0	0.0	0.0	0.0	191.7	49.8	30.1	18.2	11.0	6.6	4.0	26.4
27	Taz	198.0	1.2	0.7	0.4	2584.4	4632.7	1464.3	874.0	638.6	347.6	209.9	126.8	923.2
28	Kobuk	42.2	6.4	3.8	2.3	412.7	220.1	123.9	74.7	67.7	31.9	19.3	11.6	84.7
29	Coppermine	5.8	0.0	0.0	0.0	78.0	143.0	49.3	28.0	16.9	10.2	6.2	3.7	28.4
30	Hayes(Trib. Arctic Ocean)	5.4	0.0	0.0	0.0	0.0	166.9	45.0	26.6	16.1	9.7	5.9	3.5	23.3
31	Pur	148.1	0.1	0.1	0.1	1437.3	3032.3	923.0	559.0	579.3	266.2	160.8	97.2	600.3
32	Varzuga	9.4	0.0	0.0	0.0	136.8	37.7	22.0	13.3	22.5	28.0	10.2	6.2	23.9
33	Ponoy	25.4	0.0	0.0	0.0	317.0	87.6	51.2	35.7	53.7	78.8	27.6	16.7	57.8
34	Kovda	6.1	0.0	0.0	0.0	176.4	56.1	30.5	18.5	14.2	15.6	6.6	4.0	27.3
35	Back	65.4	0.0	0.0	0.0	1114.3	1643.1	527.6	318.2	198.1	117.6	71.0	42.9	341.5
36	Kem	47.1	0.1	0.1	528.5	780.4	270.5	156.3	98.0	86.0	139.2	50.7	30.6	182.3
37	Nadym	76.6	0.0	0.0	0.0	900.9	1469.2	470.9	292.2	300.6	137.5	83.1	50.2	315.1
38	Quoich	8.3	0.0	0.0	0.0	0.0	243.3	69.9	39.9	25.6	15.0	9.0	5.5	34.7
39	Mezen	74.6	0.9	0.5	783.4	2104.3	771.1	416.2	250.1	159.9	173.5	77.3	46.7	404.9
40	Iijoki	18.8	0.0	0.0	265.2	199.4	79.2	46.6	30.2	31.2	60.0	20.5	12.4	63.6
41	Joekulsa A Fjoellum	15.0	0.0	0.0	1.1	150.8	118.6	47.4	29.6	29.4	44.3	16.5	9.8	38.5
42	Svarta, Skagafiroi	11.0	0.0	5.9	24.7	72.8	78.5	29.6	17.8	15.2	30.4	13.8	7.2	25.6
43	Oulujoki	46.2	0.2	0.2	1079.7	340.6	187.9	112.5	74.6	83.3	142.1	49.5	29.9	178.9
44	Lagarfljot	22.6	0.0	0.0	4.5	238.1	148.6	64.8	46.3	51.5	62.0	25.6	14.8	56.6
45	Thelon	95.7	0.0	0.0	0.0	1238.2	2149.9	674.0	405.4	337.3	171.9	103.8	62.7	436.6
46	Angerman	68.7	0.1	0.1	805.4	704.9	537.1	246.5	166.7	152.9	191.0	74.2	44.8	249.4
47	Thjorsa	84.4	6.6	18.3	224.6	344.7	264.5	132.4	116.8	133.7	168.2	95.7	57.8	137.3
48	Northern Dvina(Severnaya I	204.3	7.0	4.4	8913.4	3875.8	1908.4	1118.5	675.2	411.7	419.5	194.4	117.6	1487.5
49	Oelfusa	75.7	0.0	64.6	345.3	138.1	137.0	113.1	79.6	94.2	131.7	86.9	64.0	110.9
50	Nizhny Vyg (Soroka)	41.3	0.0	0.0	1090.2	324.6	185.3	110.7	66.9	84.1	125.2	44.8	27.1	175.0
51	Kuskokwim	253.9	0.3	0.2	0.1	3324.1	1599.7	1094.0	1094.7	1058.3	479.7	274.5	165.8	778.8
52	Vuoksi	67.0	0.2	0.2	2105.8	593.7	345.7	207.8	128.7	112.1	192.1	77.4	44.0	322.9
53	Onega	45.0	0.6	0.4	2126.8	680.5	380.4	225.2	136.0	87.4	111.3	47.0	28.2	322.4
54	Susitna	254.1	0.4	0.3	730.8	1663.8	1781.1	1053.9	819.0	968.5	547.7	274.1	165.6	688.3
55	Kymijoki	37.3	0.2	0.2	914.1	264.0	151.6	91.3	55.4	43.3	61.7	62.3	24.5	142.1
56	Neva	239.0	2.1	1.8	6433.8	1912.0	1093.8	654.5	396.8	340.7	517.3	337.3	154.8	1007.0
57	Ferguson	8.1	0.0	0.0	0.0	0.0	210.7	59.2	34.2	28.4	14.5	8.8	5.3	30.8
58	Copper	218.2	0.0	0.0	3.5	1509.3	2042.9	1488.1	837.2	792.6	443.1	236.8	143.0	642.9
59	Gloma	112.6	0.3	2.4	687.9	673.6	723.1	409.7	297.6	280.1	268.8	135.5	73.9	305.5
60	Kokemaenjoki	47.3	0.3	0.3	732.4	218.6	123.2	74.3	45.1	29.4	30.6	100.4	31.0	119.4
61	Vaenern-Goeta	239.7	0.6	452.0	1075.6	459.5	275.9	163.8	141.4	158.4	295.1	323.4	207.4	316.1
62	Thlewiaza	18.4	2.1	1.2	0.7	366.8	133.2	67.9	40.8	39.7	18.8	11.4	6.9	59.0
63	Alsek	57.2	0.0	0.0	116.9	522.3	554.2	220.1	147.7	192.4	136.0	62.1	37.5	170.5
64	Volga	618.3	29.4	149.5	28026.4	10217.8	5716.4	3446.0	2149.4	1377.5	1297.4	633.1	380.4	4503.5
65	Dramselv	52.0	0.1	19.4	246.7	291.6	280.2	146.8	133.2	144.0	126.6	61.0	34.1	128.0
66	Arnaud	112.9	0.0	0.0	0.0	126.5	1128.4	412.9	308.7	375.7	272.6	122.5	74.0	244.5
67	Nushagak	103.5	0.0	0.0	261.7	987.4	354.0	208.5	258.6	309.7	276.6	112.3	67.8	245.0
68	Seal	25.3	0.3	0.2	0.1	706.9	287.0	145.7	86.5	83.1	48.3	26.1	15.8	118.8
69	Taku	86.0	0.0	0.0	161.4	501.6	459.4	206.6	156.7	205.5	252.7	93.4	56.4	181.7
70	Narva	142.1	0.2	0.2	1203.7	387.4	215.9	127.9	84.0	74.6	146.4	291.9	93.3	230.6
71	Stikine	365.4	120.7	101.6	331.4	1940.0	2386.6	1153.3	745.7	763.1	675.8	389.7	274.1	770.6
72	Churchill	157.9	4.0	2.5	680.0	2624.2	1544.9	752.3	426.3	392.0	344.9	155.0	93.6	598.1
73	Feuilles (Riviere Aux)	134.7	0.0	0.0	0.0	507.9	1039.9	410.2	351.5	386.4	354.7	146.3	88.3	285.0
74	George	151.6	0.0	0.0	0.0	991.6	1007.9	792.3	523.3	523.7	340.2	164.6	99.4	382.9
75	Caniapiscau	459.4	0.2	0.1	0.1	3234.1	2495.1	1361.1	1246.0	1333.9	1204.8	498.1	300.8	1011.1
76	Western Dvina (Daugava)	154.5	0.4	0.4	1836.2	595.4	342.7	203.3	123.9	85.1	169.9	309.6	101.5	326.9
77	Aux Melezes	127.5	0.0	0.0	0.0	1086.7	650.2	368.3	345.4	357.4	339.2	138.3	83.5	291.4
78	Baleine, Grande Riviere De	76.0	0.0	0.0	0.0	854.0	301.2	224.4	196.6	226.2	198.9	82.5	49.8	184.1
79	Spey	95.6	40.7	35.0	27.3	19.8	11.6	8.8	10.5	16.3	31.5	49.2	58.4	33.7
80	Kamchatka	138.0	0.0	0.0	0.0	1920.8	1439.9	1079.8	520.8	383.8	309.4	149.8	90.5	502.7
81	Nass	210.8	0.0	162.1	688.3	1204.1	926.8	409.5	303.2	367.9	521.5	284.1	138.2	434.7
82	Skeena	215.6	0.0	208.4	898.3	1763.9	1432.2	616.5	402.1	387.5	462.7	303.7	141.4	569.4
83	Nelson	339.7	8.1	7.2	5862.3	4172.9	3028.8	1614.9	1003.0	915.9	822.0	360.0	218.1	1529.4
84	Hayes(Trib. Hudson Bay)	68.0	1.4	0.9	0.5	1402.4	755.7	410.9	222.3	159.6	146.2	68.0	41.1	273.1
85	Gudena	51.9	23.1	20.7	15.4	8.4	4.9	3.2	2.1	3.0	11.6	25.0	31.5	16.7
86	Skjern A	70.3	26.6	24.2	17.8	10.2	5.8	3.7	4.5	14.1	33.0	38.5	45.7	24.5
87	Neman	148.0	0.7	508.0	1762.7	628.2	349.0	207.6	128.4	81.2	93.5	319.4	97.3	360.3
88	Fraser	791.0	209.7	472.1	3071.4	5379.4	3099.4	1486.4	888.0	650.8	723.7	628.1	501.3	1491.8
89	Severn(Trib. Hudson Bay)	118.6	0.0	0.0	194.0	1593.0	665.1	362.8	231.7	286.0	337.8	128.8	77.8	333.0
90	Amur	2619.6	11.8	11.9	8383.6	11458.9	10773.1	9089.3	9961.6	10071.5	5691.1	2835.3	1714.5	6051.8
91	Tweed	115.2	48.0	45.0	31.6	22.1	14.5	10.9	12.3	18.2	34.4	62.3	73.2	40.6
92	Grande Riviere De La Balei	96.4	0.0	0.0	0.0	611.5	395.7	228.2	219.5	248.2	273.8	104.7	63.2	186.8
93	Grande Riviere	545.8	0.2	0.1	0.1	4147.7	2038.4	1308.9	1228.8	1437.5	1536.5	591.8	357.5	1099.4
94	Winisk	178.0	0.4	0.3	694.1	2038.0	886.7	489.9	295.8	470.2	497.3	191.6	115.7	488.2
95	Churchill, Fleuve (Labrador)	340.5	0.0	0.0	0.0	3044.8	2194.2	1205.7	937.9	912.7	903.4	369.7	223.3	844.4
96	Dniepr	169.9	9.0	1972.1	4102.7	1572.0	896.9	562.3	356.6	212.7	178.4	289.9	117.8	870.0
97	Ural	64.8	1.1	33.0	2151.9	763.5	432.4	294.2	189.6	105.6	64.4	117.6	42.9	355.1
98	Wisla	189.4	7.6	2562.6	1362.2	838.2	515.5	356.3	248.6	171.6	155.9	231.5	169.6	567.4
99	Don	61.6	5.9	827.7	2873.8	1141.8	656.1	454.5	303.4	160.2	88.8	86.2	42.4	558.5
100	Oder	206.5	370.5	1179.3	663.9	432.1	292.4	189.5	136.4	101.1	95.7	125.4	209.2	333.5

Basin ID	Basin name	Blue water availability (Mm^3/month)												
		Jan	Feb	Mar	Apr	May	Jun	Jul	Aug	Sep	Oct	Nov	Dec	Average
101	Elbe	571.7	518.9	979.9	740.4	454.5	315.4	229.6	178.5	145.2	173.1	290.9	371.5	414.1
102	Trent	138.3	73.6	60.6	42.7	27.5	16.1	11.3	10.2	9.8	13.4	39.6	80.5	43.6
103	Weser	702.3	401.7	379.1	279.6	180.5	123.5	100.9	96.8	100.4	163.9	320.3	458.8	275.6
104	Attawapiskat	24.3	0.0	0.0	0.0	439.1	122.6	71.1	42.9	70.1	65.8	26.4	16.0	73.2
105	Eastmain	236.6	0.0	0.0	511.6	1837.7	979.9	584.5	507.2	597.6	675.9	256.8	155.1	528.6
106	Manicouagan (Riviere)	250.6	0.1	0.0	104.9	1733.3	1384.9	738.2	596.7	644.0	696.0	271.8	164.2	548.7
107	Columbia	2392.2	2251.9	4180.7	7637.6	11038.1	7369.9	3710.4	2336.7	1458.1	1092.4	1336.4	1584.7	3865.8
108	Little Mecatina	88.9	0.0	0.0	0.0	1231.3	435.7	301.1	215.8	200.8	252.6	96.5	58.3	240.1
109	Natashquan (Riviere)	58.2	0.0	0.0	31.2	745.4	324.0	241.7	161.6	138.2	158.5	63.2	38.2	163.3
110	Rhine	2635.8	1531.5	1618.9	1920.5	1602.6	1211.0	955.9	836.7	794.4	902.9	1309.3	1672.7	1416.0
111	Albany	162.6	0.0	0.0	1682.4	1840.8	765.4	429.7	261.2	355.3	486.4	176.5	106.6	522.2
112	Saguenay (Riviere)	396.8	0.3	0.3	2249.0	2250.3	1627.5	1011.7	820.1	933.1	1149.7	434.7	260.3	927.8
113	Thames	145.2	89.5	72.2	47.5	27.4	15.7	9.9	6.5	4.4	3.7	26.4	79.0	44.0
114	Nottaway	437.7	0.0	0.0	2690.3	3197.5	1528.0	1037.6	854.3	1016.7	1258.3	487.6	287.0	1066.3
115	Rupert	62.3	0.0	0.0	158.9	640.2	237.8	159.2	135.5	151.7	178.9	67.6	40.8	152.8
116	Moose(Trib. Hudson Bay)	200.0	0.1	0.1	2266.8	1995.8	860.2	505.5	318.9	427.3	604.9	217.2	131.2	627.3
117	St.Lawrence	2767.0	70.2	5921.1	26475.1	10246.2	6389.5	3920.6	2606.4	3061.0	4207.6	4579.8	1943.1	6015.6
118	Danube	3073.8	2594.0	6011.3	6879.8	5415.4	3830.1	2816.6	2249.6	2042.7	2537.2	3013.0	2515.1	3581.6
119	Seine	685.4	498.2	436.7	332.8	201.0	119.0	80.5	60.9	40.5	46.8	139.2	340.2	248.4
120	Dniestr	81.6	2.7	629.5	520.4	305.7	226.1	158.4	125.3	102.2	125.9	140.5	54.4	206.1
121	Southern Bug	7.8	1.6	368.2	193.4	103.9	62.3	41.1	27.8	15.4	8.7	5.5	3.6	69.9
122	Mississippi	15984.8	13314.3	22387.2	20429.5	16710.6	11375.6	7271.9	5466.0	3619.5	2440.0	4002.6	7786.6	10899.0
123	Skagit	190.3	68.6	290.6	349.6	195.0	98.2	57.4	34.7	21.7	81.8	150.3	120.1	138.2
124	Aral Drainage	509.4	832.3	2756.8	4028.2	4787.4	4275.2	3593.2	2586.1	1620.9	770.0	334.9	308.3	2200.2
125	Loire	1138.4	793.2	782.4	638.6	456.1	295.0	187.4	142.0	109.2	160.9	367.9	652.5	477.0
126	Rhone	1463.4	673.0	1179.2	1265.1	1117.7	889.3	538.0	442.5	455.4	695.4	1046.5	1030.9	899.7
127	Saint John	308.6	0.4	0.4	2672.8	895.7	612.2	385.8	252.2	276.9	435.9	574.3	202.5	551.5
128	Po	855.3	400.0	707.2	1106.1	1279.5	890.5	588.3	478.9	462.8	589.6	696.5	571.4	718.8
129	Penobscot	131.0	0.1	96.4	1110.9	375.6	245.0	146.7	90.3	84.3	136.9	265.4	86.0	230.7
130	St.Croix	34.1	0.0	0.0	288.4	95.1	60.3	34.3	20.4	17.7	33.4	70.5	22.4	56.4
131	Kuban	201.8	255.1	332.9	424.8	388.7	318.9	278.7	165.2	124.4	94.2	105.6	125.2	234.6
132	Connecticut	186.9	1.6	623.2	995.8	505.7	311.2	197.1	132.1	152.2	209.9	339.9	133.0	315.7
133	Liao He	135.6	4.1	44.1	331.4	453.3	434.2	532.2	797.6	521.3	276.7	156.4	90.9	314.8
134	Garonne	624.5	383.6	433.9	457.9	382.3	222.7	151.8	118.8	94.9	131.5	215.0	383.2	300.0
135	Ishikari	171.9	0.5	0.5	878.0	383.7	216.1	158.8	155.3	235.6	287.0	296.4	112.9	241.4
136	Merrimack	76.2	1.7	631.6	357.9	207.2	126.9	75.6	46.8	42.5	72.3	156.6	50.6	153.8
137	Hudson	200.1	5.3	895.4	994.1	549.1	331.2	212.7	145.1	148.5	209.0	353.0	145.7	349.1
138	Colorado(Pacific Ocean)	64.7	19.9	147.6	609.3	1180.7	864.0	478.1	330.7	225.4	148.1	74.1	46.3	349.1
139	Klamath	602.1	714.8	709.3	609.3	400.3	210.3	139.7	91.1	55.2	29.3	79.0	299.0	328.3
140	Ebro	844.0	564.0	557.2	575.2	524.0	326.4	238.4	170.8	102.0	112.3	174.7	484.9	389.5
141	Rogue	218.0	217.7	181.8	171.7	124.5	60.5	38.1	24.0	14.6	8.3	42.4	102.9	100.4
142	Douro	782.7	606.4	820.9	596.8	411.5	250.4	210.1	164.1	80.8	43.0	107.9	330.0	367.0
143	Susquehanna	418.4	248.0	1762.9	1183.4	777.5	489.6	304.5	200.5	173.5	267.4	499.9	331.8	554.8
144	Luan He	34.9	9.4	29.6	56.0	81.6	42.9	192.8	243.8	145.0	71.6	38.0	23.2	80.7
145	Kura	111.9	37.4	141.8	607.1	822.3	553.8	380.4	279.3	182.7	153.2	138.5	82.7	290.9
146	Dalinghe	11.7	0.6	1.3	6.7	13.3	16.0	17.0	77.3	42.0	24.7	12.9	7.9	19.3
147	Delaware	328.1	160.3	812.5	451.1	358.0	208.6	142.5	113.6	117.5	152.4	281.3	258.8	282.1
148	Sacramento	1075.2	1225.5	1249.8	1013.6	627.4	473.8	412.9	341.6	234.1	102.3	68.7	287.9	592.7
149	Huang He (Yellow River)	540.5	121.7	464.0	1033.2	1635.5	1820.4	2061.9	1961.0	1986.1	1174.6	610.4	360.6	1147.5
150	Kizilirmak	39.8	167.3	240.4	479.4	315.7	160.9	107.5	78.7	48.2	26.4	16.7	25.7	142.2
151	Yongding He	34.9	80.8	294.6	505.9	500.4	255.1	345.9	479.5	214.4	88.6	37.0	27.6	238.7
152	Tejo	542.7	440.4	652.3	412.4	268.7	166.9	144.4	111.9	58.6	24.9	27.3	236.4	257.2
153	Sakarya	69.7	203.4	225.2	177.7	101.6	69.0	54.7	48.5	31.4	13.8	5.3	15.0	84.6
154	Eel (Calif.)	273.2	251.6	185.4	108.1	60.8	34.7	21.1	12.8	7.7	4.6	3.1	115.9	89.9
155	Tigris & Euphrates	3102.9	3322.0	4428.0	5129.6	3747.0	2056.3	1473.7	1147.6	708.8	458.3	715.1	1524.7	2317.8
156	Potomac	271.2	232.7	341.0	274.8	189.7	125.0	74.1	51.0	37.7	42.7	78.5	150.5	155.7
157	Guadiana	49.3	143.2	323.8	204.5	114.3	114.2	140.6	123.0	60.4	21.1	6.1	3.2	108.6
158	Kitakami	138.0	34.5	271.5	216.5	160.4	110.4	116.2	118.0	126.4	155.2	165.7	105.7	143.2
159	Mogami	193.9	165.5	207.9	155.0	111.2	75.3	78.6	71.3	86.1	104.0	148.2	204.8	133.5
160	Han-Gang (Han River)	162.9	2.5	274.5	367.7	233.4	240.1	862.6	782.9	539.4	277.7	202.1	108.1	337.8
161	Guadalquivir	135.4	261.3	628.0	395.5	211.0	200.8	222.1	191.1	96.2	37.1	13.0	32.1	202.0
162	San Joaquin	141.4	165.9	257.1	267.1	227.4	219.7	253.6	238.7	163.7	64.4	15.0	20.5	169.5
163	James	320.1	247.9	249.6	190.8	134.2	85.8	51.7	33.8	24.5	32.0	76.3	171.5	134.9
164	Bravo	52.7	17.0	40.9	131.4	267.3	187.5	182.7	215.9	240.5	128.0	60.9	40.8	130.5
165	Shinano, Chikuma	220.1	161.5	160.0	367.5	372.8	258.2	248.4	201.4	234.4	235.0	230.1	208.9	241.5
166	Roanoke	369.0	293.6	292.0	217.2	145.0	89.7	63.2	47.7	36.8	31.8	66.5	170.8	151.9
167	Naktong	101.6	54.9	151.1	196.7	139.5	191.9	397.5	375.8	334.7	176.5	109.0	69.0	191.5
168	Indus	2383.8	1928.5	3639.7	4374.0	3702.9	3653.0	6475.8	8147.2	6268.9	3860.0	2171.8	1351.5	3996.4
169	Tone	187.2	52.2	138.4	211.5	196.6	194.7	211.8	248.2	312.3	308.2	196.0	136.6	199.5
170	Salinas	2.4	6.9	11.7	7.4	6.5	9.4	13.3	13.6	8.6	1.9	0.5	0.3	6.9
171	Pee Dee	564.9	476.3	469.2	321.8	188.9	117.9	112.7	91.0	92.1	76.0	109.1	276.8	241.4
172	Chelif	46.9	56.7	56.4	35.1	20.6	17.6	16.2	13.3	8.9	3.4	1.8	7.9	23.7
173	Cape Fear	318.4	260.4	245.0	163.5	105.8	70.8	78.0	76.3	69.7	53.2	71.9	145.1	138.2
174	Tenryu	117.0	58.2	102.3	167.8	149.7	172.3	163.3	144.9	205.3	177.7	127.2	87.8	139.4
175	Santee	161.0	158.9	150.4	99.3	59.7	38.7	34.8	34.0	28.1	24.8	30.1	79.1	74.9
176	Kiso	130.5	43.6	70.5	169.7	167.3	179.4	192.4	150.0	193.6	156.0	121.6	84.2	138.2
177	Yangtze(Chang Jiang)	8149.3	4134.6	9668.1	17316.8	22991.0	27351.3	23858.2	22676.6	20204.9	13565.9	8255.8	5104.0	15273.0
178	Yodo	212.7	107.2	130.9	142.7	116.6	148.8	135.1	102.0	153.6	164.1	130.5	126.5	139.2
179	Sebou	211.8	216.2	249.7	187.0	106.6	69.2	56.9	40.2	28.4	15.3	39.4	112.7	111.1
180	Alabama River & Tombigbee	1879.6	2154.0	2446.6	1722.6	965.9	536.9	335.5	211.0	134.2	84.3	93.0	669.7	936.1
181	Savannah	300.2	314.1	331.7	211.7	116.8	72.2	54.4	38.1	37.5	33.0	47.7	114.6	139.3
182	Gono (Go)	91.5	48.9	53.0	55.2	43.6	70.8	74.0	38.3	54.7	55.2	45.9	53.8	57.1
183	Huai He	254.3	206.3	475.5	869.7	886.2	875.0	951.0	760.1	737.9	380.1	230.6	164.7	565.9
184	Apalachicola	577.9	769.5	877.1	603.2	314.5	182.5	130.3	106.3	80.6	47.2	40.4	207.0	328.0
185	Brazos	149.4	174.9	143.5	197.3	186.0	117.5	192.5	165.6	101.7	35.9	16.0	73.4	129.5
186	Altamaha	265.7	449.8	515.0	311.1	165.9	94.9	62.8	46.6	36.5	22.5	14.4	84.9	172.5
187	Mekong	6136.7	81.2	116.9	188.0	1169.3	6856.7	17030.8	21415.0	21592.3	12858.8	7086.8	4099.6	8219.3
188	Colorado(Caribbean Sea)	93.3	137.7	110.6	124.2	104.5	68.6	99.5	91.0	63.1	23.2	7.2	45.7	80.7
189	Trinity(Texas)	117.7	164.5	153.2	194.7	168.0	73.1	46.9	30.4	19.6	13.3	10.5	51.2	86.9
190	Pearl	396.8	402.6	426.3	342.7	218.8	119.6	78.5	51.4	32.6	20.0	29.7	150.6	189.1
191	Sabine	222.0	267.0	257.3	261.5	187.4	91.2	55.3	33.2	20.4	12.4	17.4	59.0	123.7
192	Suwannee	208.6	252.5	263.2	159.5	83.1	60.1	122.6	151.4	142.9	77.2	42.3	105.5	139.1
193	Yaqui	3.2	6.0	12.6	14.6	7.2	4.8	9.8	12.3	8.9	5.8	3.0	3.2	7.6
194	Nile	4144.8	462.1	1332.0	3260.1	3755.8	4351.5	9676.8	16108.3	13324.8	7288.9	4494.2	2864.4	5922.0
195	Brahmaputra	5680.5	109.9	556.6	3370.4	9356.6	20739.8	25504.2	25649.4	21443.0	11769.2	6277.3	3729.5	11182.2
196	St.Johns	75.8	37.5	45.2	25.3	14.5	13.5	66.2	95.0	159.3	146.2	67.1	45.2	65.9
197	Nueces	0.8	1.7	4.3	5.7	7.2	8.3	13.3	9.9	4.9	2.1	1.1	0.8	5.0
198	San Antonio	2.5	2.6	2.9	4.7	5.1	3.9	5.0	3.7	2.0	1.1	0.8	1.8	3.0
199	Irrawaddy	5381.8	68.5	198.9	860.3	2195.2	11965.1	19709.4	22367.5	18624.5	11876.0	6047.8	3534.8	8569.1
200	Fuerte	68.1	18.1	9.7	8.0	7.1	7.8	33.2	152.8	161.4	81.3	41.6	49.9	53.2
201	Xi Jiang	1674.6	536.5	986.5	2152.4	5159.4	8919.3	8252.7	8479.4	5027.7	2953.6	1791.7	1089.6	3918.6
202	Bei Jiang	125.9	70.8	384.4	909.3	1374.9	1402.6	766.2	646.1	431.8	220.1	133.7	82.7	545.7
203	San Pedro	38.6	0.5	0.9	1.2	1.4	0.7	27.4	146.4	197.6	73.8	41.3	26.1	46.3

Basin ID	Basin name	Blue water availability (Mm^3/month)												
		Jan	Feb	Mar	Apr	May	Jun	Jul	Aug	Sep	Oct	Nov	Dec	Average
204	Dong Jiang	172.2	21.0	223.2	615.6	1151.6	1293.5	976.4	940.0	667.6	311.7	186.9	113.6	556.1
205	Mahi	176.8	31.6	49.2	44.6	27.5	7.6	528.2	885.3	630.5	277.8	173.2	114.7	245.6
206	Damodar	251.6	22.3	32.2	9.0	3.0	50.7	299.0	950.2	931.6	429.5	263.2	170.1	284.4
207	Niger	4355.6	19.8	51.2	259.6	966.6	2958.9	7669.6	16126.9	18141.0	9525.3	4765.9	2855.7	5641.3
208	Narmada	481.4	86.2	180.1	226.3	227.6	89.0	1470.5	2332.6	1749.0	751.5	459.1	317.3	697.6
209	Brahmani River (Bhahmani)	312.4	6.7	10.6	8.5	7.7	109.9	777.7	1587.8	1207.1	611.8	339.1	209.9	432.5
210	Mahanadi(Mahahadi)	750.2	21.7	31.6	30.2	31.6	31.5	1041.8	4292.3	2975.6	1339.8	823.9	514.4	990.4
211	Santiago	136.2	23.2	48.7	53.1	33.6	41.0	205.6	530.4	622.5	282.7	158.3	98.5	186.2
212	Panuco	308.1	19.2	37.3	39.5	27.6	72.6	402.7	498.3	1231.3	704.9	354.2	209.3	325.4
213	Godavari	1361.1	125.3	248.0	295.5	314.3	178.3	2692.3	5505.7	5324.4	2419.1	1469.4	966.9	1741.7
214	Tapti	248.7	33.7	61.3	73.1	79.0	29.8	673.1	1033.9	1023.2	424.8	266.7	177.2	343.7
215	Sittang	450.9	0.7	1.3	1.3	3.4	770.5	1570.5	2008.8	1666.5	980.2	498.5	295.7	687.3
216	Armeria	12.1	1.6	3.4	5.8	5.5	2.0	0.7	8.1	58.4	29.5	14.3	9.6	12.6
217	Ca	289.6	11.2	5.4	4.7	30.9	116.8	375.4	535.7	866.0	553.6	330.4	193.2	276.1
218	Chao Phraya	836.7	63.6	104.0	107.5	98.7	328.0	1108.4	1921.4	3201.9	2014.4	1162.3	612.0	963.2
219	Krishna	850.0	122.1	251.0	271.1	280.0	651.3	3320.2	3037.0	2359.2	1342.1	934.0	685.6	1175.3
220	Senegal	325.8	2.0	3.0	2.3	2.4	89.6	612.5	1692.9	1372.7	654.2	358.5	215.3	444.3
221	Papaloapan	375.4	2.5	3.2	3.2	2.3	95.5	474.6	882.2	1156.1	885.3	438.8	252.3	381.0
222	Grisalva	2371.9	240.8	132.9	122.0	329.4	1724.5	2361.9	2646.1	4101.1	3860.2	2199.7	1575.4	1805.5
223	Verde	75.7	0.6	1.3	1.4	0.9	2.0	73.1	170.1	329.6	178.8	82.8	50.2	80.5
224	Mae Klong	310.9	4.2	6.8	6.7	215.3	716.0	1050.9	1166.4	1116.5	710.3	343.0	206.2	487.8
225	Tranh (Nr Thu Bon)	401.4	9.0	4.9	3.3	14.3	58.2	176.6	255.8	354.1	544.8	483.8	323.0	219.1
226	Penner	113.7	6.9	10.4	9.0	8.8	8.3	31.7	30.2	36.6	72.1	203.5	97.1	52.4
227	Volta	504.4	1.5	16.0	58.4	165.7	523.3	714.2	1680.5	2259.3	1085.4	548.9	331.0	657.4
228	Lempa	177.6	0.6	1.5	2.1	2.3	109.5	316.4	393.6	608.4	459.1	195.3	117.0	198.6
229	Gambia	150.2	0.1	0.1	0.1	0.1	29.0	184.4	615.6	693.3	307.6	163.2	98.5	186.8
230	Grande De Matagalpa	357.7	17.5	9.3	6.0	7.5	270.2	538.9	488.8	540.4	590.0	349.6	249.6	285.5
231	Cauvery	418.3	31.9	77.1	69.5	70.2	216.2	733.9	661.1	514.8	469.5	555.5	369.8	349.0
232	San Juan	1044.7	106.7	56.5	52.3	207.2	790.6	955.7	958.8	1223.9	1470.0	968.0	784.4	718.2
233	Geba	107.4	0.7	0.9	0.9	0.7	15.8	82.7	362.8	461.0	241.4	116.5	70.7	121.8
234	Corubal	176.6	0.1	0.1	0.1	0.1	58.6	353.5	718.8	633.2	416.1	192.9	115.8	222.2
235	Magdalena	5423.6	690.5	1286.0	2835.1	4242.2	3726.6	3011.1	3096.0	3658.2	6369.3	6357.9	4183.3	3740.0
236	Comoe	89.6	0.7	0.9	21.0	61.2	184.6	135.4	203.6	302.0	204.4	108.1	59.3	114.2
237	Orinoco	14712.0	1981.7	3222.0	9639.5	19300.5	27474.1	31384.6	27826.1	22407.3	20689.1	15477.9	9366.2	16956.7
238	Bandama	267.4	0.8	1.1	23.7	61.4	304.2	211.1	589.8	1114.9	637.3	294.6	176.3	306.9
239	Oueme	91.8	0.2	0.2	1.4	48.2	195.3	253.0	264.2	378.7	207.6	99.8	60.2	133.4
240	Sassandra	452.3	0.3	0.6	25.8	61.9	450.1	664.6	927.7	1664.6	1083.2	520.7	297.5	512.4
241	Shebelle	225.2	9.9	10.9	506.4	351.0	205.1	318.8	401.0	388.9	336.4	322.1	158.3	269.5
242	Mono	24.4	0.1	2.9	10.1	25.2	66.1	71.4	63.9	94.4	57.9	26.5	16.0	38.2
243	Congo	38781.7	18567.6	25336.9	27793.8	18695.0	12504.4	11096.3	14292.0	18079.1	22224.7	21780.2	24631.5	21148.6
244	Atrato	1781.7	459.4	547.3	863.5	1124.9	1175.4	1195.3	1219.4	1337.9	1428.2	1406.4	1107.4	1137.2
245	Cuyuni	1959.6	627.4	566.0	853.6	1974.3	2695.6	2604.3	2026.9	1089.2	717.4	733.1	1314.3	1430.1
246	Cavally	459.1	21.2	44.2	106.6	310.6	683.7	489.5	388.4	841.0	837.6	587.1	331.0	425.0
247	Tano	64.3	0.0	6.5	37.3	109.4	271.3	140.0	67.4	96.2	162.2	84.4	43.7	90.2
248	Cross	797.3	0.3	121.6	237.0	469.3	946.4	1565.6	1826.7	2324.9	2163.2	890.4	522.9	988.8
249	Sanaga	962.5	0.6	47.3	400.8	813.7	1168.0	1585.8	1923.4	2745.2	2671.6	1076.6	631.2	1168.9
250	Pra	75.7	0.6	20.5	56.4	131.6	263.3	138.2	65.6	129.2	207.9	96.6	49.5	102.9
251	Davo	26.7	0.0	0.0	0.4	1.9	108.6	56.6	25.3	47.6	57.7	38.6	17.9	31.8
252	Essequibo	1013.8	395.3	422.1	586.4	1432.7	2611.4	2344.0	1591.5	787.3	487.8	375.0	621.1	1055.7
253	Kelantan	715.0	87.8	68.5	83.5	86.8	96.3	98.8	112.2	270.6	417.0	486.3	543.8	255.6
254	Corantijn	262.7	165.9	342.2	710.9	2259.5	2636.2	1942.3	1216.8	602.5	362.3	218.8	138.5	904.9
255	Coppename	310.2	278.9	308.1	414.3	804.7	915.7	772.2	466.3	232.1	139.1	84.0	64.2	399.1
256	Kinabatangan	564.0	172.5	135.1	134.6	135.1	229.8	157.6	228.5	293.7	277.7	249.9	381.8	246.7
257	Maroni	769.9	916.7	1065.3	1531.4	2216.1	2029.1	1418.6	868.5	448.4	269.9	163.0	113.1	984.2
258	San Juan (Columbia - Pacifi	1212.9	440.7	523.2	691.3	824.8	782.5	781.0	794.2	820.7	905.9	905.8	781.2	788.7
259	Amazonas	190075.1	141017.2	162784.5	171575.3	142636.9	112877.6	84821.2	59821.4	47615.3	48736.0	59615.2	91142.2	109393.2
260	Pahang	1155.2	258.5	283.3	392.4	387.5	252.1	172.7	164.7	279.1	543.0	722.5	835.2	453.8
261	Nyong	253.8	0.0	77.2	230.6	363.4	300.9	139.6	135.6	486.9	678.0	340.0	166.5	264.4
262	Oyapock	856.4	808.7	963.5	1250.0	1320.6	1151.1	705.3	414.0	226.3	136.7	82.5	117.3	669.4
263	Rajang	4099.4	1702.6	1931.5	2042.2	1987.1	1553.9	1376.4	1371.2	1848.5	2225.8	2371.2	2455.4	2080.4
264	Ntem	411.1	1.7	88.6	324.7	519.1	361.6	158.2	94.1	311.7	886.2	638.6	285.7	340.1
265	Ogooue	4545.3	1513.9	3146.8	4155.3	3829.4	1590.0	923.6	558.3	501.0	1730.3	4718.4	3488.7	2558.4
266	Rio Araguari	945.4	1075.5	1387.2	1692.3	1623.6	1424.8	854.6	497.3	275.3	166.2	100.4	79.7	843.5
267	Mira	414.6	251.9	258.2	286.0	406.5	391.9	249.7	245.8	271.1	233.1	256.6	182.6	287.3
268	Esmeraldas	584.4	847.6	1135.2	1348.6	933.7	500.8	279.8	175.2	117.8	115.8	151.1	193.3	532.0
269	Tana	58.1	2.9	7.8	102.6	141.2	66.6	36.7	22.7	14.0	20.7	52.1	61.0	48.9
270	Daule & Vinces	546.2	816.1	1052.0	939.3	483.9	296.4	183.1	129.0	94.5	89.4	91.6	80.2	400.1
271	Rio Gurupi	98.3	409.7	929.4	902.7	685.2	430.3	282.6	152.9	90.2	54.5	32.9	20.6	340.8
272	Rio Capim	307.6	1121.3	1760.0	1585.2	1185.0	756.5	523.3	313.9	175.7	105.2	63.5	41.1	661.5
273	Tocantins	16587.4	13165.4	14385.3	9144.3	4822.1	2913.5	1786.2	1117.1	784.6	795.8	3307.2	8908.7	6476.5
274	Kouilou	801.5	480.1	790.2	1061.3	613.7	285.3	172.5	104.5	63.3	38.3	226.1	600.2	436.4
275	Nyanga	232.0	138.3	188.2	208.9	104.7	53.5	32.3	19.5	11.8	7.1	138.6	161.0	108.0
276	Rio Parnaiba	407.9	749.4	1466.6	1398.4	624.8	344.7	209.5	128.3	78.8	48.5	31.4	111.8	466.7
277	Rio Itapecuru	16.9	245.0	652.0	650.7	330.3	176.7	104.2	62.9	38.1	23.1	13.9	8.5	193.5
278	Rio Acarau	4.5	6.4	162.1	215.4	129.3	60.3	35.9	22.0	13.6	8.4	5.2	3.3	55.5
279	Pangani	6.9	4.5	7.0	40.6	98.7	48.0	30.5	17.4	12.0	7.7	5.7	5.5	23.7
280	Rio Pindare	50.7	460.9	943.4	905.1	534.9	283.1	163.0	97.8	59.1	35.7	21.6	13.1	297.4
281	Sepik	3075.2	1911.1	2533.1	2436.2	1879.5	1484.8	1359.2	1362.5	1541.5	1620.1	1617.0	1836.5	1888.1
282	Rio Mearim	71.3	542.7	1089.1	931.5	464.4	250.6	149.1	90.2	54.6	33.0	20.0	12.3	309.1
283	Chira	15.2	41.7	75.9	68.3	27.6	17.3	12.4	10.4	7.6	4.4	4.6	3.4	24.1
284	Rufiji	367.2	765.4	1536.8	1760.9	805.4	425.8	257.1	155.8	94.7	57.6	35.3	83.8	528.8
285	Rio Jaguaribe	12.8	2.3	323.9	548.9	312.5	172.4	103.1	64.0	42.7	28.4	18.5	12.1	136.8
286	Purari	1417.8	840.0	1040.9	1067.0	892.0	707.0	590.5	613.0	770.6	723.2	727.2	843.2	852.7
287	Ruvu	24.3	24.5	72.4	190.5	144.4	57.5	34.8	21.2	13.0	7.9	4.8	14.7	50.8
288	Rio Paraiba	7.5	0.4	0.7	17.9	50.8	102.8	79.4	41.7	23.1	14.6	9.3	5.9	29.5
289	Solo (Bengawan Solo)	571.1	483.1	486.7	344.4	189.9	106.3	66.4	44.5	34.2	21.2	50.0	220.0	218.2
290	Sao Francisco	5543.3	3050.3	2796.4	1502.3	931.4	1005.7	989.5	668.7	333.1	253.7	986.0	3746.2	1817.2
291	Brantas	359.2	348.2	352.5	249.5	139.4	79.0	48.7	32.7	24.4	14.2	32.1	106.2	148.8
292	Santa	91.1	112.6	130.2	79.9	42.2	25.0	15.7	11.4	8.8	15.9	25.7	26.6	48.8
293	Zambezi	14656.0	16403.8	13703.0	6465.6	3653.6	2187.3	1332.7	840.7	523.6	324.4	261.1	4300.2	5387.7
294	Rio Vaza-Barris	3.4	0.3	0.3	0.3	12.3	30.2	35.5	20.5	10.0	6.2	3.8	2.4	10.4
295	Rio Itapicuru	11.3	0.5	0.4	1.4	19.8	52.6	129.1	72.6	33.9	20.5	12.4	7.7	30.2
296	Rio Paraguacu	54.8	37.1	60.1	141.0	298.9	233.2	293.3	175.6	91.0	53.5	38.9	32.8	125.9
297	Canete	51.5	46.7	46.2	25.7	13.4	8.2	5.1	3.5	2.6	8.6	12.5	22.8	20.6
298	Rio De Contas	59.4	24.0	51.6	111.4	110.0	89.6	91.6	58.5	35.8	22.2	33.0	45.0	61.0
299	Roper	87.0	307.4	336.4	119.8	71.7	43.3	26.2	15.9	9.7	5.9	3.5	2.1	85.8
300	Daly	156.7	536.8	496.4	183.8	110.4	66.8	40.4	24.5	14.9	9.1	5.4	3.4	137.4
301	Drysdale	5.2	56.7	54.1	19.3	11.7	7.0	4.3	2.6	1.6	0.9	0.6	0.3	13.7
302	Parana	21195.9	14570.6	13669.3	10027.2	7997.7	6909.3	4501.0	3607.4	3909.3	4882.2	6576.5	11498.5	9112.1
303	Durack	0.0	0.4	0.3	0.1	0.1	0.0	0.0	0.0	0.0	0.0	0.0	0.0	0.1
304	Rio Prado	139.2	56.7	71.5	83.5	48.7	37.4	33.8	21.3	11.7	7.3	27.5	109.8	54.0
305	Victoria	0.0	4.1	2.8	1.1	0.7	0.4	0.2	0.1	0.1	0.1	0.0	0.0	0.8
306	Mitchell(N. Au)	216.0	1243.4	1175.4	523.3	287.0	172.0	104.2	63.5	38.9	24.0	14.5	8.6	322.6

Basin ID	Basin name	Blue water availability (Mm^3/month)												
		Jan	Feb	Mar	Apr	May	Jun	Jul	Aug	Sep	Oct	Nov	Dec	Average
307	Majes	178.8	199.0	175.2	87.2	46.5	28.0	16.9	10.5	6.9	6.3	5.9	72.6	69.5
308	Ord	0.0	0.8	0.3	0.5	0.8	1.0	1.3	1.5	1.6	1.3	0.7	0.0	0.8
309	Jequitinhonha	841.6	329.8	286.2	172.1	94.3	59.4	40.7	25.2	14.8	12.1	135.1	630.5	220.1
310	Macarthur	0.1	0.2	11.1	2.9	1.8	1.1	0.6	0.4	0.2	0.1	0.1	0.1	1.6
311	Fitzroy	1.1	89.3	98.1	33.5	20.2	12.2	7.4	4.5	2.7	1.6	1.0	0.6	22.7
312	Gilbert	36.6	275.0	225.2	85.6	51.2	30.9	18.7	11.3	6.8	4.2	2.5	1.5	62.5
313	Mucuri	266.4	82.5	63.0	50.8	33.4	22.8	17.7	10.0	5.8	4.3	42.5	209.2	67.4
314	Rio Doce	2512.7	1059.7	848.4	492.3	260.3	156.9	96.6	60.4	37.4	23.6	466.9	1819.8	652.9
315	Save	440.7	671.2	488.1	213.1	125.5	77.3	48.6	34.8	26.2	17.0	8.3	69.7	185.0
316	Burdekin	138.0	735.9	732.5	377.4	200.2	119.6	72.8	45.4	29.3	19.2	11.9	6.5	207.4
317	Tsiribihina	1926.4	1960.9	1809.9	830.9	470.9	287.4	178.6	109.0	65.8	39.7	59.8	568.3	692.3
318	Buzi	260.9	383.4	377.8	150.5	87.6	53.0	32.2	19.8	12.3	7.7	4.5	22.2	117.7
319	Loa	0.1	0.1	0.1	0.1	0.1	0.1	0.1	0.1	0.1	0.1	0.1	0.1	0.1
320	Limpopo	376.0	611.8	560.6	286.6	153.4	100.3	71.8	66.8	66.1	49.4	31.9	61.7	203.0
321	De Grey	0.0	0.0	0.0	0.0	0.0	0.0	0.0	0.0	0.0	0.0	0.0	0.0	0.0
322	Paraiba Do Sul	1476.8	821.1	764.8	436.0	247.8	151.9	94.0	61.7	52.9	118.0	326.7	880.1	452.6
323	Fortescue	0.0	0.0	0.0	0.0	0.0	0.0	0.0	0.0	0.0	0.0	0.0	0.0	0.0
324	Mangoky	371.6	440.8	370.5	179.8	103.6	66.1	44.1	27.3	16.6	10.0	11.3	66.7	142.4
325	Fitzroy	14.5	381.9	427.0	177.1	98.8	60.2	38.6	26.5	20.4	16.0	10.9	7.0	106.6
326	Orange	371.4	419.1	449.2	280.5	161.4	97.9	68.5	64.0	62.3	72.5	107.0	176.8	194.2
327	Ashburton	0.0	0.0	0.0	0.0	0.0	0.0	0.0	0.0	0.0	0.0	0.0	0.0	0.0
328	Gascoyne	0.0	0.0	0.0	0.0	0.0	0.0	0.0	0.0	0.0	0.0	0.0	0.0	0.0
329	Rio Ribeira Do Iguape	435.0	331.5	285.2	177.5	147.1	156.4	107.6	88.6	120.2	172.1	154.8	197.5	197.8
330	Incomati	208.0	223.7	205.5	103.4	55.4	34.7	22.7	16.8	13.5	8.7	25.9	91.3	84.1
331	Murray	573.7	275.9	300.2	222.1	255.9	430.7	502.3	633.0	674.2	667.5	462.9	400.1	449.9
332	Murchison	0.0	0.0	0.0	0.0	0.0	0.0	0.0	0.0	0.0	0.0	0.0	0.0	0.0
333	Maputo	185.0	143.8	123.7	64.9	35.9	22.9	15.0	11.6	9.2	6.6	22.5	93.4	61.2
334	Uruguay	3140.5	1126.7	1622.4	2798.1	3389.9	3831.6	3268.9	3124.1	3775.3	4032.1	2572.9	1917.4	2883.3
335	Tugela	151.7	157.4	150.6	74.5	40.9	25.3	17.3	14.9	13.4	13.0	17.2	75.2	62.6
336	Colorado (Argentinia)	700.2	69.2	44.2	27.1	74.6	141.4	168.3	186.0	181.7	474.4	630.5	515.1	267.7
337	Rio Jacui	1001.0	498.4	580.2	784.2	979.4	1158.6	1067.9	1029.0	1148.6	1027.3	681.2	558.8	876.2
338	Huasco	18.4	3.3	2.0	1.2	0.7	0.5	0.3	0.3	0.3	0.3	0.1	9.2	3.1
339	Limari	23.6	20.3	8.2	4.2	2.5	5.6	3.7	3.5	2.9	3.1	2.3	6.4	7.2
340	Negro (Uruguay)	260.3	17.4	98.4	299.8	454.8	662.4	644.6	658.0	675.8	564.6	310.8	169.3	401.4
341	Groot-Vis	2.1	4.8	4.6	2.8	2.3	1.9	2.0	2.6	4.3	5.5	3.8	3.8	3.4
342	Salado	202.3	4.9	14.6	157.5	245.6	247.0	221.1	191.9	252.2	313.6	283.6	153.5	190.7
343	Blackwood	15.9	0.1	0.1	0.1	0.0	6.0	50.9	81.5	60.3	33.4	17.4	10.5	23.0
344	Rapel	223.9	35.6	22.7	13.3	102.1	274.7	271.3	237.1	175.4	140.3	82.5	136.3	142.9
345	Negro (Argentinia)	492.3	19.8	69.4	202.0	805.1	1171.8	1242.1	1215.0	1006.1	873.6	632.5	348.1	673.1
346	Biobio	302.6	5.8	59.7	183.8	747.3	957.3	1008.5	934.1	826.2	594.6	361.9	209.0	515.9
347	Waikato	242.0	87.2	76.4	120.3	254.3	328.5	323.4	310.4	277.8	271.2	216.1	156.3	222.0
348	South Esk	37.3	1.8	2.1	6.5	15.3	41.7	78.6	94.3	80.6	71.6	43.4	27.1	41.7
349	Chubut	167.5	14.0	34.4	67.2	254.8	452.7	519.4	623.2	449.3	290.8	179.2	116.1	264.0
350	Clutha	205.8	84.2	96.6	138.8	136.9	138.9	128.4	144.7	179.7	191.4	152.5	131.9	144.2
351	Baker	385.8	126.0	219.8	327.7	451.9	507.2	550.8	529.7	429.8	376.5	315.9	256.6	373.1
352	Santa Cruz	330.4	77.0	112.0	236.4	318.1	370.1	315.4	493.0	644.2	645.4	321.2	208.5	339.3
353	Ganges	6436.4	2196.3	3289.5	2579.3	2584.4	5564.7	15724.9	25704.0	19394.6	9568.4	6524.3	3925.2	8624.3
354	Salween	1673.2	19.1	118.5	302.3	569.3	2411.1	4929.8	6413.6	5517.3	3631.9	1947.4	1104.5	2386.5
355	Hong(Red River)	956.0	16.1	20.9	50.9	313.3	1487.9	3689.4	4528.9	3276.8	1920.5	1086.7	629.8	1498.1
356	Lake Chad	1374.1	27.1	28.9	36.0	52.6	245.4	1597.8	7283.3	5590.2	2810.0	1481.8	898.2	1785.5
357	Okavango	815.0	1297.8	1723.8	794.3	408.2	246.6	149.2	90.4	55.0	33.4	20.1	176.4	484.2
358	Tarim	48.4	15.4	53.9	118.7	331.4	569.1	665.3	472.1	270.3	108.2	59.0	32.8	228.7
359	Horton	2.5	0.1	0.0	0.0	15.8	63.0	18.7	11.0	6.6	4.0	2.4	1.5	10.5
360	Hornaday	2.5	0.0	0.0	0.0	0.0	36.3	29.1	14.0	7.4	4.5	2.7	1.6	8.2
361	Conception	0.1	0.4	0.7	1.0	0.8	0.9	0.9	1.2	1.1	0.8	0.3	0.2	0.7
362	Ulua	347.2	15.5	8.5	6.3	3.9	102.1	358.3	399.5	627.2	549.7	383.4	252.4	254.5
363	Patacua	342.2	23.7	10.8	6.5	3.9	14.2	138.7	178.6	287.7	435.2	360.5	258.7	171.7
364	Coco	553.6	28.7	14.1	8.7	9.7	339.7	648.8	591.8	656.1	787.8	557.7	408.2	383.8
365	Ocona	107.9	116.4	103.4	49.0	27.0	16.2	9.8	6.1	4.0	9.8	14.2	47.0	42.6
366	Cuanza	2072.1	1513.0	2059.2	1749.4	710.6	425.5	257.3	155.8	94.5	61.5	60.8	1091.7	854.3
367	Cunene	365.8	516.0	1048.1	585.0	264.6	159.8	96.5	58.4	35.3	22.6	19.6	128.5	275.0
368	Doring	8.8	4.1	5.1	3.0	0.8	18.4	29.7	35.2	27.2	21.0	12.3	8.6	14.5
369	Gamka	13.1	2.9	5.8	8.1	8.2	10.2	9.0	12.6	22.3	21.2	17.4	11.2	11.8
370	Groot- Kei	0.5	1.3	3.4	2.6	1.7	1.2	1.1	1.2	1.6	1.7	1.2	0.9	1.5
371	Lurio	920.9	1237.0	1217.8	504.4	287.0	173.4	104.7	63.3	38.2	23.1	13.9	93.6	389.8
372	Messalo	188.3	363.7	439.4	219.8	109.6	66.2	40.0	24.1	14.6	8.8	5.3	3.2	123.6
373	Rovuma	1821.2	3053.0	3618.4	1863.8	921.1	555.9	335.8	202.8	122.5	74.0	44.7	103.3	1059.7
374	Galana	37.4	1.6	7.0	119.5	130.9	65.5	35.5	20.9	12.9	8.0	40.6	39.2	43.2
375	Pyasina	94.1	1.0	0.6	0.4	0.3	1716.5	565.8	374.8	360.8	162.8	98.4	59.4	286.2
376	Popigay	17.0	0.2	0.1	0.1	0.0	410.5	150.8	82.1	50.2	29.4	17.7	10.7	64.1
377	Fuchun Jiang	250.7	393.4	649.9	607.5	805.2	1114.6	484.0	287.4	256.5	177.3	141.5	114.5	440.2
378	Min Jiang	342.0	452.2	1288.9	1252.7	1944.2	2128.8	970.0	745.1	576.5	409.9	268.2	176.1	879.6
379	Han Jiang	85.9	37.9	248.2	402.1	758.5	943.6	489.5	425.1	331.0	156.6	93.3	56.8	335.7
380	Mamberamo	3089.3	1890.8	2522.1	2293.6	1944.7	1568.5	1677.1	1592.9	1739.5	1309.5	1434.7	1726.2	1899.1
381	Lorentz	98.6	89.0	103.1	95.0	69.3	50.8	56.2	50.8	69.2	39.9	55.4	52.5	69.1
382	Eilanden	1086.2	666.9	767.1	750.2	713.5	648.9	647.1	618.0	674.3	540.3	565.0	655.8	694.4
383	Uwimbu	1790.5	1079.0	1275.8	1198.4	1202.0	1086.5	1052.7	1025.3	1077.5	910.6	871.5	1080.3	1137.5
384	Sungai Kajan	2153.9	818.5	1097.7	1323.9	1374.1	1159.1	1021.0	983.6	1286.2	1400.0	1564.0	1365.9	1295.6
385	Sungai Mahakam	3721.2	1575.3	2027.0	2751.3	2520.0	1933.7	1366.3	1130.0	1190.3	1480.5	2230.6	2523.3	2037.5
386	Sungai Kapuas	5898.3	2671.2	3045.5	3103.2	2702.7	2058.3	1478.7	1321.6	1787.3	2755.1	3458.7	3524.5	2817.1
387	Batang Kuantan	764.1	292.1	346.4	463.4	365.1	215.1	128.1	105.1	167.7	345.0	515.9	520.5	352.4
388	Batang Hari	2183.7	907.2	1090.0	1250.5	958.0	564.2	353.4	308.1	487.6	882.3	1254.6	1435.3	972.9
389	Flinders	0.1	18.0	5.6	3.1	1.9	1.1	0.7	0.4	0.3	0.2	0.1	0.1	2.6
390	Leichhardt	6.4	7.0	2.4	1.4	0.9	0.5	0.3	0.2	0.1	0.1	0.0	0.0	1.6
391	Escaut (Schelde)	270.3	141.2	120.8	94.5	57.0	33.8	22.3	16.0	11.0	12.7	74.7	149.2	83.6
392	Issyk-Kul	60.1	9.7	426.4	677.0	859.2	798.0	600.5	334.3	214.4	117.0	76.9	41.0	351.2
393	Balkhash	54.7	1.2	439.8	809.3	818.0	581.4	420.5	273.7	181.0	82.5	89.0	36.3	315.6
394	Eyre Lake	0.0	0.1	0.1	0.1	0.1	0.1	0.1	0.1	0.1	0.1	0.1	0.1	0.1
395	Lake Mar Chiquita	98.0	118.9	205.2	100.8	52.2	32.5	22.5	17.4	16.2	17.1	19.0	27.5	60.6
396	Lake Turkana	416.5	1.8	20.1	397.7	660.1	883.0	1343.7	1565.6	1309.8	827.0	500.3	278.9	683.7
397	Dead Sea	121.0	136.3	90.8	57.9	51.4	40.7	39.4	33.7	19.3	11.7	6.4	23.6	52.7
398	Suriname	437.4	383.0	417.7	584.5	945.9	1002.8	794.8	484.1	242.6	145.8	88.1	105.3	469.3
399	Lake Titicaca	1424.9	1192.7	995.0	590.4	378.7	210.5	131.7	110.4	93.9	137.6	135.7	519.0	493.4
400	Lake Vattern	21.8	0.1	129.2	107.2	49.2	27.2	16.3	12.0	8.0	16.5	39.2	17.1	37.0
401	Great Salt Lake	13.1	47.5	70.9	156.4	183.2	126.8	96.3	70.1	42.7	24.3	13.7	13.3	71.5
402	Lake Taymur	144.7	0.0	0.0	0.0	0.0	2947.2	1277.6	659.7	502.8	260.1	157.1	94.9	503.7
403	Daryacheh-Ye Orumieh	31.2	20.6	101.3	305.5	308.0	147.6	93.6	69.4	42.8	25.3	27.5	23.4	99.7
404	Van Golu	23.6	0.1	25.4	223.6	275.7	97.8	59.0	36.8	22.5	23.0	46.1	15.5	70.8
405	Ozero Sevan	1.7	0.1	0.8	15.4	21.4	11.2	6.2	4.2	2.6	2.6	2.8	1.2	5.8
