# Supplementary material for: Global Monthly Water Scarcity: Blue Water Footprints versus Blue Water Availability
Source: PLoS One. 2012 Feb 29;7(2):e32688. doi: 10.1371/journal.pone.0032688 (PMC3290560; doi:10.1371/journal.pone.0032688)
Supplement: Table S4 — Monthly blue water scarcity for the world's major river basins. (PDF) [file pone.0032688.s008.pdf]

Table S4. Monthly blue water scarcity for the world's major river basins

Period: 1996-2005

Basin ID	Basin name	Population (thousands)	Water scarcity (%)													Number of months per year that a basin faces low, moderate, significant or severe water scarcity			
			Jan	Feb	Mar	Apr	May	Jun	Jul	Aug	Sep	Oct	Nov	Dec	Average	Low	Moderate	Significant	Severe
1	Khatanga	4.63	0.0028	0.0748	0.124	0.205	0.0198	0.0002	0.0003	0.0006	0.001	0.0018	0.003	0.005	0.0365	12	0	0	0
2	Olenek	5.96	0.0046	0.0353	0.0585	0.0968	0.0086	0.0003	0.001	0.0018	0.003	0.0051	0.0084	0.014	0.0198	12	0	0	0
3	Anabar	1.4	0.0025	0.0155	0.0257	0.0425	0.0704	0.0003	0.0006	0.0013	0.0022	0.0037	0.0062	0.0102	0.0151	12	0	0	0
4	Yana	24.5	0.0259	0.319	0.528	0.873	0.146	0.0031	0.0034	0.0061	0.0127	0.021	0.0347	0.0575	0.169	12	0	0	0
5	Yenisei	8453	0.434	9.43	15.5	1.46	0.207	0.27	0.407	0.441	0.338	0.375	0.494	0.802	2.51	12	0	0	0
6	Indigirka	41.8	0.0208	0.22	0.364	0.603	0.0877	0.0024	0.0027	0.0058	0.0109	0.0182	0.0301	0.0498	0.118	12	0	0	0
7	Lena	1285	0.0771	1.86	3.07	3.36	0.014	0.0098	0.0145	0.0196	0.0228	0.051	0.0845	0.14	0.727	12	0	0	0
8	Omoloy	2.88	0.102	4.07	6.71	11	18.1	0.0064	0.0098	0.0212	0.0371	0.0624	0.103	0.171	3.37	12	0	0	0
9	Tana (NO, FI)	6.52	0.102	675	675	675	0.0026	0.0094	0.016	0.0265	0.0337	0.0491	0.094	0.156	169	9	0	0	3
10	Colville	0.98	0.0134	1.82	3	4.96	0.0546	0.0013	0.0013	0.0024	0.0043	0.0077	0.0127	0.021	0.825	12	0	0	0
11	Alazeya	6.65	0.0342	0.202	0.334	0.552	0.914	0.0033	0.0113	0.02	0.0332	0.0549	0.0909	0.151	0.2	12	0	0	0
12	Anderson	0.09	0.0055	0.861	1.42	2.36	0.0001	0.0004	0.0007	0.0011	0.0019	0.0031	0.0052	0.0086	0.389	12	0	0	0
13	Kolyma	138	0.0352	0.813	1.35	2.23	0.0245	0.0051	0.0036	0.0082	0.0125	0.0235	0.0389	0.0643	0.383	12	0	0	0
14	Tuloma	209	2.1	17.1	27.8	44.8	0.115	0.363	0.663	1.1	1.67	1.64	3.6	5.94	8.91	12	0	0	0
15	Muonio	57.8	0.382	4.18	6.9	0.257	0.0274	0.0495	0.0733	0.142	0.186	0.294	0.541	0.895	1.16	12	0	0	0
16	Yukon	131	0.0732	1.41	2.32	0.388	0.0081	0.0089	0.0138	0.0228	0.0291	0.0475	0.0846	0.14	0.379	12	0	0	0
17	Palyavaam	7.83	0.0697	624	643	656	664	0.0039	0.0072	0.0141	0.0209	0.0388	0.0642	0.106	216	8	0	0	4
18	Kemijoki	149	0.331	50	79	0.271	0.0179	0.0659	0.111	0.173	0.149	0.118	0.312	0.517	10.9	12	0	0	0
19	Mackenzie	494	0.293	19	30.9	0.194	0.0244	0.0237	0.0397	0.0741	0.112	0.164	0.288	0.474	4.3	12	0	0	0
20	Noatak	2.04	0.0331	6.28	10.3	17	0.0129	0.0026	0.0047	0.0083	0.0082	0.0188	0.0311	0.0514	2.81	12	0	0	0
21	Anadyr	11.2	0.009	8.37	13.7	22.5	0.0043	0.0005	0.001	0.0019	0.0026	0.005	0.0083	0.0138	3.72	12	0	0	0
22	Pechora	606	0.233	18.4	30	0.396	0.0098	0.0148	0.0353	0.0596	0.073	0.129	0.226	0.374	4.16	12	0	0	0
23	Lule	35.6	0.103	32.6	52.3	0.0287	0.0083	0.0085	0.0169	0.0289	0.0337	0.047	0.0956	0.158	7.12	12	0	0	0
24	Kalixaelven	35	0.396	4.7	7.75	0.0799	0.0251	0.041	0.0883	0.153	0.225	0.262	0.539	0.892	1.26	12	0	0	0
25	Ob	29372	4.01	140	205	0.593	1.02	2.93	7.26	9.79	6.64	3.66	4.17	6.68	32.7	10	1	0	1
26	Ellice	0	0	0	0	0	0	0	0	0	0	0	0	0	0	12	0	0	0
27	Taz	15.1	0.0144	2.38	3.94	6.49	0.0011	0.0006	0.002	0.0033	0.0045	0.0082	0.0136	0.0225	1.07	12	0	0	0
28	Kobuk	2.2	0.0264	0.175	0.29	0.48	0.0027	0.0051	0.009	0.0149	0.0164	0.0349	0.0578	0.0956	0.101	12	0	0	0
29	Coppermine	0.431	0.0499	11.7	19.2	31.2	0.0037	0.002	0.0058	0.0103	0.0171	0.0282	0.0468	0.0774	5.2	12	0	0	0
30	Hayes(Trib. Arctic Ocea	0	0	0	0	0	0	0	0	0	0	0	0	0	0	12	0	0	0
31	Pur	197	0.252	676	676	676	0.0259	0.0123	0.0404	0.0667	0.0643	0.14	0.232	0.384	169	9	0	0	3
32	Varzuga	4.14	0.0832	676	676	676	0.0057	0.0208	0.0356	0.0589	0.0349	0.028	0.0766	0.127	169	9	0	0	3
33	Ponoy	3.41	0.0254	676	676	676	0.002	0.0074	0.0126	0.0181	0.012	0.0082	0.0234	0.0387	169	9	0	0	3
34	Kovda	33.2	1.03	676	676	676	0.0356	0.112	0.206	0.34	0.441	0.403	0.945	1.56	169	9	0	0	3
35	Back	0.011	0.0001	676	676	676	0	0	0	0	0	0.0001	0.0001	0.0002	169	9	0	0	3
36	Kem	75.6	0.314	135	198	0.028	0.0189	0.0546	0.0945	0.151	0.172	0.106	0.291	0.482	27.9	10	1	1	0
37	Nadym	43.6	0.108	207	286	370	0.0092	0.0056	0.0176	0.0283	0.0275	0.0601	0.099	0.165	72	9	0	0	3
38	Quoich	0	0	0	0	0	0	0	0	0	0	0	0	0	0	12	0	0	0
39	Mezen	42.1	0.107	8.87	14.6	0.0102	0.0038	0.0103	0.0192	0.0319	0.0499	0.046	0.103	0.171	2	12	0	0	0
40	Iijoki	62.8	0.737	676	676	0.0524	0.0697	0.176	0.299	0.462	0.446	0.232	0.679	1.12	113	10	0	0	2
41	Joekulsa A Fjoellum	0.762	0.015	676	676	0.198	0.0015	0.0019	0.0048	0.0076	0.0077	0.0051	0.0137	0.0229	113	10	0	0	2
42	Svarta, Skagafiroi	2.06	0.0554	676	0.104	0.0246	0.0084	0.0077	0.0206	0.0342	0.04	0.02	0.0442	0.0845	56.3	11	0	0	1
43	Oulujoki	197	0.942	195	271	0.0403	0.131	0.247	0.436	0.704	0.577	0.31	0.879	1.45	39.3	10	0	1	1
44	Lagarfljot	3.05	0.04	676	676	0.202	0.0038	0.0061	0.0139	0.0195	0.0175	0.0146	0.0352	0.0609	113	10	0	0	2
45	Thelon	2.17	0.0151	104	157	225	0.0012	0.0007	0.0021	0.0036	0.0043	0.0084	0.014	0.0231	40.5	9	1	1	1
46	Angerman	66.6	0.171	109	163	0.0146	0.0167	0.0219	0.0476	0.0704	0.0768	0.0615	0.158	0.262	22.7	10	1	1	0
47	Thjorsa	1.99	0.0065	0.0826	0.0299	0.0024	0.0016	0.0021	0.0041	0.0047	0.0041	0.0033	0.0057	0.0095	0.013	12	0	0	0
48	Northern Dvina(Severna	1718	1.59	46.3	73.4	0.0365	0.0942	0.206	0.349	0.55	0.811	0.776	1.67	2.77	10.7	12	0	0	0
49	Oelfusa	7.59	0.0296	676	0.0347	0.0065	0.0162	0.0163	0.0198	0.0281	0.0238	0.017	0.0258	0.035	56.3	11	0	0	1
50	Nizhny Vyg (Soroka)	86.9	0.398	676	676	0.0151	0.0507	0.0888	0.149	0.246	0.196	0.131	0.367	0.608	113	10	0	0	2
51	Kuskokwim	11.4	0.0227	20.5	33.3	53.4	0.0018	0.0037	0.0053	0.0053	0.0054	0.012	0.021	0.0347	8.94	12	0	0	0
52	Vuoksi	750	2.47	676	676	0.0784	0.289	0.521	0.94	1.67	1.68	0.868	2.13	3.75	114	10	0	0	2
53	Onega	176	0.741	59.1	92.6	0.0157	0.0561	0.112	0.188	0.282	0.388	0.3	0.71	1.18	13	12	0	0	0
54	Susitna	30.1	0.06	34	54.5	0.021	0.0113	0.0107	0.0156	0.0188	0.0158	0.0279	0.0557	0.0921	7.4	12	0	0	0
55	Kymijoki	588	3.45	676	676	0.141	0.517	0.937	1.63	2.93	3.37	2.1	2.06	5.25	114	10	0	0	2
56	Neva	4245	3.36	379	458	0.125	0.553	1.1	1.68	2.95	2.6	1.55	2.38	5.19	71.5	10	0	0	2
57	Ferguson	0.007	0.0006	676	676	676	676	0	0.0001	0.0001	0.0002	0.0003	0.0005	0.0008	225	8	0	0	4
58	Copper	4.95	0.0114	676	676	0.72	0.0017	0.0012	0.0017	0.003	0.0031	0.0056	0.0105	0.0174	113	10	0	0	2
59	Gloma	759	1.53	676	72	0.251	0.283	0.413	1.04	1.56	0.778	0.643	1.28	2.34	63.2	11	0	0	1

Basin ID	Basin name	Population (thousands)	Water scarcity (%)													Number of months per year that a basin faces low, moderate, significant or severe water scarcity			
			Jan	Feb	Mar	Apr	May	Jun	Jul	Aug	Sep	Oct	Nov	Dec	Average	Low	Moderate	Significant	Severe
60	Kokemaenjoki	773	3.61	532	581	0.233	0.876	1.69	3.04	5.76	7.29	5.64	1.7	5.52	95.7	10	0	0	2
61	Vaenern-Goeta	1486	1.11	408	0.586	0.247	0.622	1.16	2.11	2.25	1.74	0.898	0.82	1.28	35	11	0	0	1
62	Thlewiaza	0.052	0.0019	0.0168	0.0279	0.0462	0.0001	0.0003	0.0005	0.0008	0.0009	0.0018	0.003	0.005	0.0088	12	0	0	0
63	Alsek	1.03	0.0115	676	676	0.0056	0.0013	0.0012	0.003	0.0045	0.0034	0.0048	0.0106	0.0175	113	10	0	0	2
64	Volga	61274	18.8	394	77.7	0.541	5.95	14	32.6	44.8	25.8	12.6	19.1	30.5	56.4	11	0	0	1
65	Dramselv	282	1.24	676	3.31	0.26	0.224	0.295	0.756	0.68	0.488	0.508	1.05	1.88	57.2	11	0	0	1
66	Arnaud	0	0	0	0	0	0	0	0	0	0	0	0	0	0	12	0	0	0
67	Nushagak	1.47	0.0072	676	676	0.0028	0.0008	0.0021	0.0036	0.0029	0.0024	0.0027	0.0066	0.011	113	10	0	0	2
68	Seal	1.07	0.0284	2.29	3.78	6.23	0.001	0.0025	0.0049	0.0083	0.0086	0.0149	0.0275	0.0455	1.04	12	0	0	0
69	Taku	1.9	0.0137	676	676	0.0073	0.0024	0.0026	0.0057	0.0075	0.0057	0.0047	0.0126	0.0209	113	10	0	0	2
70	Narva	1217	1.13	676	676	0.133	0.489	0.899	1.6	2.74	2.4	1.1	0.549	1.72	114	10	0	0	2
71	Stikine	1.57	0.0027	0.0083	0.0098	0.003	0.0005	0.0004	0.0009	0.0013	0.0013	0.0015	0.0026	0.0036	0.003	12	0	0	0
72	Churchill	90.5	0.383	15.1	24.6	0.1	0.031	0.0486	0.101	0.179	0.18	0.193	0.398	0.647	3.49	12	0	0	0
73	Feuilles (Riviere Aux)	0	0	0	0	0	0	0	0	0	0	0	0	0	0	12	0	0	0
74	George	0.032	0.0001	676	676	676	0	0	0	0	0	0.0001	0.0001	0.0002	169	9	0	0	3
75	Caniapiscau	0.921	0.0013	3.57	5.89	9.7	0.0002	0.0002	0.0005	0.0005	0.0005	0.0005	0.0012	0.002	1.6	12	0	0	0
76	Western Dvina (Daugav	2723	1.88	676	676	0.168	0.832	1.32	2.13	4	4.18	1.72	0.937	2.86	114	10	0	0	2
77	Aux Melezes	0	0	0.0001	0.0001	0.0002	0	0	0	0	0	0	0	0	0	12	0	0	0
78	Baleine, Grande Riviere	0	0	0	0	0	0	0	0	0	0	0	0	0	0	12	0	0	0
79	Spey	33.2	0.0276	0.0648	0.0754	0.0967	0.133	0.228	0.302	0.252	0.162	0.0838	0.0536	0.0452	0.127	12	0	0	0
80	Kamchatka	25.8	0.0354	676	676	676	0.0025	0.0034	0.0045	0.0094	0.0128	0.0158	0.0326	0.054	169	9	0	0	3
81	Nass	2.98	0.0095	676	0.0123	0.0029	0.0017	0.0022	0.0049	0.0066	0.0054	0.0038	0.007	0.0144	56.3	11	0	0	1
82	Skeena	44.9	0.139	676	0.144	0.0334	0.017	0.021	0.0487	0.0746	0.0774	0.0649	0.0988	0.212	56.4	11	0	0	1
83	Nelson	5566	10.6	445	518	1.67	4.34	6.75	22	53.2	30.8	13.2	15.4	18.2	94.9	10	0	0	2
84	Hayes(Trib. Hudson Bay	14.5	0.143	6.89	11.3	18.6	0.0069	0.0128	0.0236	0.0436	0.0607	0.0662	0.143	0.236	3.13	12	0	0	0
85	Gudena	450	0.772	1.73	1.94	2.61	14	59.6	112	88.2	34.2	3.58	1.61	1.27	26.8	11	1	0	0
86	Skjern A	165	0.205	0.543	0.595	0.811	2.99	23.6	71.8	25	2.33	0.436	0.374	0.315	10.8	12	0	0	0
87	Neman	5487	3.08	676	0.897	0.277	1.33	2.41	3.98	8.64	9.03	5.01	1.43	4.69	59.7	11	0	0	1
88	Fraser	1294	1.09	4.11	1.83	0.317	0.211	0.393	1.05	2.06	1.97	1.25	1.37	1.72	1.45	12	0	0	0
89	Severn(Trib. Hudson Ba	6.79	0.0383	676	676	0.0234	0.0028	0.0068	0.0125	0.0196	0.0159	0.0134	0.0353	0.0584	113	10	0	0	2
90	Amur	66165	2.34	521	583	5.2	13.2	21.5	13.9	7.61	5.18	1.63	2.45	3.78	98.4	10	0	0	2
91	Tweed	410	0.283	0.68	0.725	1.03	1.48	2.3	3.63	3.3	2.02	0.951	0.524	0.446	1.45	12	0	0	0
92	Grande Riviere De La B	0.558	0.0039	676	676	676	0.0006	0.0009	0.0016	0.0017	0.0015	0.0014	0.0036	0.0059	169	9	0	0	3
93	Grande Riviere	1.49	0.0018	5.77	9.51	15.6	0.0002	0.0005	0.0008	0.0008	0.0007	0.0006	0.0017	0.0028	2.57	12	0	0	0
94	Winisk	6.08	0.0228	9.83	16.1	0.0059	0.002	0.0046	0.0083	0.0137	0.0086	0.0082	0.0212	0.0351	2.17	12	0	0	0
95	Churchill, Fleuve (Labra	8.63	0.0169	676	676	676	0.0019	0.0026	0.0048	0.0061	0.0063	0.0064	0.0156	0.0258	169	9	0	0	3
96	Dniepr	33021	34.3	645	2.95	1.85	14.7	31.8	64.6	95	78.3	40.4	20.3	49.4	89.9	11	0	0	1
97	Ural	4063	11.9	676	23.4	1.39	18.1	52.7	129	161	107	53.2	7.66	18	105	8	2	1	1
98	Wisla	23550	22.6	566	1.67	3.18	5.99	10.4	15.1	25.9	32.3	28.8	18.5	25.3	62.9	11	0	0	1
99	Don	20898	64.5	676	4.8	3.64	44.5	98.7	174	222	156	87.4	47.5	93.6	139	8	0	2	2
100	Oder	16526	14.5	8.09	2.54	4.58	7.9	12.7	21.5	33.9	40.2	33.3	23.9	14.3	18.1	12	0	0	0
101	Elbe	22408	7.83	8.63	4.57	6.21	10.5	16.7	31.3	48	53	29.2	15.4	12	20.3	12	0	0	0
102	Trent	4841	2.79	5.23	6.36	9.06	14.9	29.3	70	73.5	53.6	29.2	9.73	4.78	25.7	12	0	0	0
103	Weser	8503	2.68	4.68	4.96	6.76	10.7	17.2	28.9	37.7	30.5	12.1	5.86	4.09	13.8	12	0	0	0
104	Attawapiskat	1.41	0.0388	676	676	676	0.0022	0.0077	0.0133	0.022	0.0135	0.0144	0.0358	0.0592	169	9	0	0	3
105	Eastmain	0.441	0.0012	676	676	0.0006	0.0002	0.0003	0.0005	0.0006	0.0005	0.0004	0.0011	0.0019	113	10	0	0	2
106	Manicouagan (Riviere)	13.9	0.037	134	196	0.0883	0.0053	0.0067	0.0126	0.0156	0.0144	0.0133	0.0341	0.0564	27.5	10	1	1	0
107	Columbia	6607	1.43	1.57	4.33	11.1	13.1	31.4	91.9	125	106	56.3	9.71	2.65	37.8	10	2	0	0
108	Little Mecatina	0.148	0.0011	676	676	676	0.0001	0.0002	0.0003	0.0005	0.0005	0.0004	0.001	0.0017	169	9	0	0	3
109	Natashquan (Riviere)	0.512	0.0059	676	676	0.011	0.0005	0.0011	0.0014	0.0021	0.0025	0.0022	0.0054	0.009	113	10	0	0	2
110	Rhine	56922	4.64	7.99	7.56	6.42	8.44	11.6	15.2	21	18.9	13.8	9.34	7.31	11	12	0	0	0
111	Albany	19.2	0.0789	597	626	0.0076	0.007	0.0168	0.03	0.0493	0.0361	0.0264	0.0726	0.12	102	10	0	0	2
112	Saguenay (Riviere)	317	0.526	676	676	0.0929	0.0934	0.136	0.213	0.26	0.225	0.182	0.48	0.802	113	10	0	0	2
113	Thames	9674	5.3	8.6	10.7	16.2	28.2	50.2	82.7	126	181	210	29.2	9.74	63.1	9	1	1	1
114	Nottaway	43.8	0.0669	676	676	0.0109	0.0092	0.0192	0.0283	0.0343	0.0288	0.0233	0.0601	0.102	113	10	0	0	2
115	Rupert	0.405	0.0043	676	676	0.0017	0.0004	0.0011	0.0017	0.002	0.0018	0.0015	0.004	0.0066	113	10	0	0	2
116	Moose(Trib. Hudson Ba	122	0.408	676	676	0.036	0.0411	0.0958	0.164	0.258	0.191	0.135	0.376	0.622	113	10	0	0	2
117	St.Lawrence	67620	13.8	545	6.47	1.46	3.99	7.07	13.7	21.7	15.6	9.57	8.37	19.7	55.6	11	0	0	1
118	Danube	81753	5.62	6.66	2.93	3.12	6.46	11.2	22.7	30.8	21	9.66	5.79	6.87	11.1	12	0	0	0
119	Seine	15598	6.75	9.29	10.6	14.7	29.6	61.1	147	256	288	121	33.3	13.6	82.6	8	2	0	2
120	Dniestr	7442	16.9	517	2.2	4.05	22.6	35.5	38	90.2	61.9	16.3	9.9	25.4	70	11	0	0	1
121	Southern Bug	3142	76.9	376	1.62	4.59	27.5	55.2	122	181	137	96.9	111	165	113	6	3	2	1

Basin ID	Basin name	Population (thousands)	Water scarcity (%)													Number of months per year that a basin faces low, moderate, significant or severe water scarcity			
			Jan	Feb	Mar	Apr	May	Jun	Jul	Aug	Sep	Oct	Nov	Dec	Average	Low	Moderate	Significant	Severe
122	Mississippi	74637	2.98	4.16	4.76	8.21	15.4	32.3	136	234	230	111	17	6.59	66.9	8	2	0	2
123	Skagit	84	0.225	0.626	0.148	0.123	0.224	0.924	2.55	4.45	3.78	0.528	0.285	0.357	1.18	12	0	0	0
124	Aral Drainage	41543	10.1	5.78	7.83	29.4	48.5	106	239	345	378	298	84	32.5	132	7	1	0	4
125	Loire	7807	2.03	2.92	3.03	4.16	8.7	22.1	88.1	177	157	30.3	6.33	3.55	42.1	10	0	2	0
126	Rhone	10015	1.94	4.24	2.51	2.53	3.71	6.53	26.2	34	15.8	4.62	2.75	2.75	8.97	12	0	0	0
127	Saint John	413	0.837	676	676	0.0966	0.289	0.447	0.834	1.83	1.07	0.595	0.45	1.28	113	10	0	0	2
128	Po	17513	4.79	10.2	5.93	3.98	9.89	22.8	105	130	45.6	9.1	5.88	7.16	30	10	2	0	0
129	Penobscot	154	0.584	676	0.794	0.0689	0.204	0.317	0.59	1.16	0.98	0.563	0.288	0.89	56.8	11	0	0	1
130	St.Croix	20.3	0.352	676	676	0.0416	0.126	0.206	0.377	0.666	0.701	0.36	0.17	0.537	113	10	0	0	2
131	Kuban	3471	3.26	2.58	1.97	2.42	19.8	50.5	105	100	29.9	10.5	6.25	5.25	28.1	10	2	0	0
132	Connecticut	2069	5.62	676	1.68	1.06	2.15	3.95	6.44	8.29	6.99	5.01	3.09	7.89	60.7	11	0	0	1
133	Liao He	30133	19.1	676	99.6	127	305	439	210	83.7	89.6	18	21.3	33.5	177	7	1	0	4
134	Garonne	3328	1.57	2.56	2.56	2.93	5.35	17.5	143	243	197	34.5	5.15	2.55	54.9	9	1	1	1
135	Ishikari	1942	1.88	676	676	0.371	0.881	6.76	9.58	12.8	4.91	1.39	1.09	2.86	116	10	0	0	2
136	Merrimack	2246	14.9	676	1.8	3.19	5.56	9.23	15.6	24.6	26.9	15.8	7.27	22.5	68.6	11	0	0	1
137	Hudson	4380	9.85	375	2.2	1.99	3.63	6.1	9.98	14.6	13.6	9.46	5.59	13.5	38.8	11	0	0	1
138	Colorado(Pacific Ocean	7755	79.7	396	175	76.4	58.3	96.5	182	237	266	248	206	191	184	4	0	3	5
139	Klamath	137	0.115	0.0972	0.123	4.72	20.4	60.7	126	167	168	98.3	3.15	0.232	54.1	9	1	2	0
140	Ebro	2922	0.571	1.95	8.37	13.6	23.4	84.4	247	308	238	61.2	6.42	1.16	82.8	9	0	0	3
141	Rogue	260	0.604	0.605	0.751	2.48	9.3	33.5	71.4	96.2	101	59.3	3.15	1.28	31.6	11	1	0	0
142	Douro	3744	0.752	1.28	2.46	6.88	18.1	101	286	375	300	106	6.79	1.78	101	7	2	0	3
143	Susquehanna	4004	4.85	8.18	1.15	1.73	2.69	4.41	8.08	13	13.2	7.85	4.06	6.12	6.28	12	0	0	0
144	Luan He	11172	40.6	676	670	659	538	529	99.8	85.1	110	87.9	38	49	299	6	1	0	5
145	Kura	13774	23.6	82.4	75.5	46.6	37.5	94.1	193	289	249	109	38.7	46.1	107	8	1	1	2
146	Dalinghe	4435	32.7	676	531	375	501	607	298	47.6	64.8	24.3	35.4	54.6	271	6	0	0	6
147	Delaware	6416	9.83	20.1	3.97	7.22	9.57	17.8	26.5	30.7	28.3	21.3	11.5	12.5	16.6	12	0	0	0
148	Sacramento	3015	1.42	1.24	3.9	28.4	106	261	386	458	462	293	59.1	5.41	172	6	1	0	5
149	Huang He (Yellow River	160715	40.3	607	512	413	260	187	168	110	50	37	30.8	48.9	205	5	1	2	4
150	Kizilirmak	4460	10.6	2.54	2.96	8.19	37.8	105	175	262	268	186	89.5	21	97.4	7	1	2	2
151	Yongding He	91200	286	676	676	676	671	671	533	402	476	398	277	352	508	0	0	0	12
152	Tejo	6899	2.07	3.26	4.64	11.6	30.5	134	306	387	343	204	51.8	4.76	124	7	1	0	4
153	Sakarya	5655	7.7	2.65	3.64	17.7	94	202	319	432	449	364	185	39.5	176	6	0	1	5
154	Eel (Calif.)	37.1	0.0689	0.0748	0.102	0.218	1.08	3.18	6.84	9.43	10.9	6.08	6.09	0.162	3.69	12	0	0	0
155	Tigris & Euphrates	49256	6.62	21.6	61.6	99.2	178	236	315	396	402	337	92.9	17.4	180	6	0	1	5
156	Potomac	3494	6.3	7.35	5.02	6.37	9.49	15.1	26.7	40	49.4	40.9	21.8	11.4	20	12	0	0	0
157	Guadiana	1601	5.25	8.13	16.3	45.4	139	368	525	571	547	454	209	99.1	249	5	1	0	6
158	Kitakami	1281	1.54	6.18	0.786	0.987	1.35	6.76	16.4	38.8	22.7	2.53	1.29	2.02	8.45	12	0	0	0
159	Mogami	1118	0.959	1.12	0.895	1.21	1.73	9.19	13.7	44.5	16.9	3.58	1.26	0.908	8	12	0	0	0
160	Han-Gang (Han River)	11656	10.4	676	6.18	4.7	9.4	15.7	3.23	2.84	5.04	6.14	8.38	15.7	63.6	11	0	0	1
161	Guadalquivir	3947	4.82	13	19.7	48	132	343	494	548	523	436	264	33.3	238	5	1	0	6
162	San Joaquin	1681	5.98	5.83	36.1	149	290	484	576	611	619	589	447	67.8	323	4	1	0	7
163	James	910	1.42	1.83	1.82	2.58	3.84	6.38	11.8	18.7	20.9	15.5	5.99	2.65	7.78	12	0	0	0
164	Bravo	9249	99.8	593	607	299	197	266	328	263	193	224	173	177	285	1	0	4	7
165	Shinano, Chikuma	2133	1.61	2.2	2.22	0.976	0.987	2.5	6.55	18.6	5.85	1.9	1.54	1.7	3.89	12	0	0	0
166	Roanoke	1472	2.02	2.54	2.57	4.27	7.78	13.6	22.6	33.4	29.9	31.2	11.5	4.37	13.8	12	0	0	0
167	Naktong	8178	11.8	22	8.12	6.35	14.7	40.7	14.7	14.8	17.1	7.23	11.2	17.6	15.5	12	0	0	0
168	Indus	212208	271	399	411	316	167	171	136	162	256	340	328	290	271	0	1	3	8
169	Tone	10011	8.89	31.9	12.1	8	8.87	15.7	25.6	42.7	16.1	6.75	8.5	12.2	16.4	12	0	0	0
170	Salinas	308	65.9	22.6	14.6	113	378	552	623	644	646	595	502	498	388	3	1	0	8
171	Pee Dee	2599	2.33	2.76	2.82	4.63	9.51	16.3	18.4	23.8	18.7	19.7	12.2	4.75	11.3	12	0	0	0
172	Chelif	3855	4.78	7.91	21.1	55	155	314	438	501	517	429	339	50.1	236	5	0	1	6
173	Cape Fear	1626	2.59	3.17	3.46	6.32	14.3	21.4	17.9	19	15.9	17.9	11.7	5.68	11.6	12	0	0	0
174	Tenryu	1398	1.92	3.86	2.2	1.34	1.51	1.76	2.6	6.56	2.11	1.34	1.77	2.56	2.46	12	0	0	0
175	Santee	3127	9.85	9.98	10.6	16.7	29.8	46.7	56.6	58.3	62	67	53.3	20	36.7	12	0	0	0
176	Kiso	1899	2.42	7.24	4.48	1.86	1.89	1.91	2.46	5.88	2.44	2.22	2.6	3.75	3.26	12	0	0	0
177	Yangtze(Chang Jiang)	384680	5.53	17.2	11.8	11.6	13.3	8.55	15.7	17.1	16.9	3.77	4.32	7.27	11.1	12	0	0	0
178	Yodo	9645	7.54	15	12.3	11.3	14	14.1	27.3	69.8	20.7	12.8	12.3	12.7	19.2	12	0	0	0
179	Sebou	5479	1.78	9.93	31.5	100	190	244	359	405	444	416	54.7	4.51	188	5	1	1	5
180	Alabama River & Tombi	4335	1.17	1.02	0.899	1.29	2.46	4.57	8.57	15.2	19.5	27.8	23.7	3.28	9.13	12	0	0	0
181	Savannah	1169	1.97	1.89	1.83	3.11	6.38	11.7	20.2	35.1	22.5	22.2	12.8	5.2	12.1	12	0	0	0
182	Gono (Go)	401	0.73	1.37	1.26	1.22	1.6	1.6	1.97	9.41	2.85	2.11	1.46	1.24	2.23	12	0	0	0
183	Huai He	97813	33.4	71.6	119	188	220	181	171	175	157	61.8	40.1	55.2	123	5	1	5	1

Basin ID	Basin name	Population (thousands)	Water scarcity (%)													Number of months per year that a basin faces low, moderate, significant or severe water scarcity			
			Jan	Feb	Mar	Apr	May	Jun	Jul	Aug	Sep	Oct	Nov	Dec	Average	Low	Moderate	Significant	Severe
184	Apalachicola	2955	2.6	1.96	1.75	3.22	9.03	24.4	57.9	118	70	84.8	39.6	7.31	35.1	11	1	0	0
185	Brazos	2820	19.8	27.8	82	85.4	152	337	551	588	589	528	235	33.2	269	5	0	1	6
186	Altamaha	2411	4.6	2.73	2.46	4.69	10.8	22.1	57.8	97.3	71.5	87.6	86.9	14.4	38.6	12	0	0	0
187	Mekong	57932	12.3	543	498	401	106	11.7	3.87	2.47	1.31	5.75	16.7	18.7	135	8	1	0	3
188	Colorado(Caribbean Sea	1667	16.6	17.3	49.9	63.8	128	313	525	576	589	533	279	30.8	260	5	1	0	6
189	Trinity(Texas)	5421	23.4	16.8	19	16.8	20.4	50.2	96	136	170	219	265	53.8	90.5	8	1	1	2
190	Pearl	623	0.795	0.784	0.741	0.931	1.52	2.79	4.39	7.06	10.2	16	10.6	2.1	4.83	12	0	0	0
191	Sabine	574	1.31	1.1	1.26	1.91	4.26	9.26	22.3	26.8	23.5	27.4	17.7	4.94	11.8	12	0	0	0
192	Suwannee	591	1.44	1.21	1.31	4.12	17.2	28.3	24	28.2	13.8	16.3	8.77	2.89	12.3	12	0	0	0
193	Yaqui	651	404	676	676	676	676	676	251	311	503	516	486	403	521	0	0	0	12
194	Nile	162346	38.5	355	202	86.5	82.8	48.3	30.2	19.9	26.5	45.6	45.3	34.9	84.6	10	0	0	2
195	Brahmaputra	67163	1.68	70	23.8	3.03	0.733	0.169	0.307	0.215	0.442	4.06	7.25	2.36	9.51	12	0	0	0
196	St.Johns	2905	19.4	42.8	39.7	74.2	162	144	26.6	19.1	9.96	10.8	22.4	32.7	50.3	10	1	1	0
197	Nueces	614	676	676	676	676	676	676	676	676	676	676	676	676	676	0	0	0	12
198	San Antonio	915	199	241	434	342	339	514	601	613	606	603	616	273	448	0	0	1	11
199	Irrawaddy	33594	1.15	91	55.1	17.5	4.91	2.83	0.676	0.395	1.3	4.67	2.44	1.15	15.2	12	0	0	0
200	Fuerte	452	4.39	38.8	149	340	448	551	126	29.2	14.5	34.5	18.9	12.4	147	7	2	0	3
201	Xi Jiang	64673	6.69	27.7	19.1	18.3	10.4	3.52	3.59	3.57	10.6	2.82	3.74	7.23	9.78	12	0	0	0
202	Bei Jiang	20751	14.1	25.2	4.65	2.17	2.61	2.88	10.4	9.45	17.4	9.47	14.3	22.3	11.2	12	0	0	0
203	San Pedro	655	5.11	603	650	665	670	668	13.4	8.24	11.5	20.6	9.29	15	278	7	0	0	5
204	Dong Jiang	13461	6.79	55.2	5.2	2.13	2.11	2	5.14	3.78	6.24	4.16	6.66	10.7	9.18	12	0	0	0
205	Mahi	11043	133	676	676	676	676	676	4.71	4.57	12.8	54.6	73.8	127	316	5	2	0	5
206	Damodar	28680	128	676	676	676	676	35.5	19.2	4.04	5.46	29.8	107	144	265	5	3	0	4
207	Niger	76931	2.69	676	312	39.4	25.6	7.69	2.49	0.883	1.06	2.17	1.36	2.55	89.5	10	0	0	2
208	Narmada	17017	123	676	676	676	676	459	2.62	2.15	6.43	33.2	46.2	126	292	5	2	0	5
209	Brahmani River (Bhahm	12476	34.6	676	676	676	676	21.2	3.01	1.15	2.57	11.4	32.7	50.2	238	8	0	0	4
210	Mahanadi(Mahahadi)	27697	65.8	676	676	676	676	242	8.98	1.56	7.05	36.9	72.6	92.4	269	7	0	0	5
211	Santiago	17992	51	675	676	676	676	216	31	15.2	28.6	81.6	88.2	109	277	6	1	0	5
212	Panuco	17860	19.2	669	674	675	675	111	14.9	15.2	7.82	14.8	19.6	38.5	245	7	1	0	4
213	Godavari	62327	103	676	676	676	676	462	20.5	9.8	12.5	48.3	95.8	147	300	5	2	0	5
214	Tapti	16928	111	676	676	676	676	676	13.4	11.5	19.4	73.2	104	156	322	4	2	1	5
215	Sittang	3191	0.769	676	676	676	171	4.55	1.09	0.386	1.86	4.54	1.83	0.867	184	8	0	1	3
216	Armeria	527	28.8	676	676	676	676	676	676	31.8	3.67	45.2	79.8	141	365	5	1	0	6
217	Ca	2652	3.47	86.5	147	239	133	16.2	3.28	0.791	0.47	0.886	1.79	3.28	53	9	2	0	1
218	Chao Phraya	26782	59.8	676	676	676	453	94.4	117	68.4	30.5	58.4	185	123	268	5	2	1	4
219	Krishna	76933	245	676	676	676	676	167	41.8	55.3	99.5	131	245	326	334	3	1	1	7
220	Senegal	5134	8.04	676	676	676	676	17.8	6.71	2.13	2.57	9.06	14.5	13.3	231	8	0	0	4
221	Papaloapan	2582	1.53	441	590	623	632	7.85	1.58	1.25	0.605	1.44	2.06	3.96	192	8	0	0	4
222	Grisalva	7037	0.382	5.24	29.3	49.3	12.9	0.878	0.415	0.521	0.195	0.21	0.67	1.57	8.46	12	0	0	0
223	Verde	862	2.23	676	676	676	676	128	3.5	1.54	0.986	1.52	4.56	8.85	238	7	1	0	4
224	Mae Klong	1568	8.26	676	676	676	9.63	1.61	4.99	5.69	4	3.45	16.1	16	175	9	0	0	3
225	Tranh (Nr Thu Bon)	1025	1.1	55.1	68.5	127	184	49.4	15.2	5.01	0.436	0.282	0.337	0.752	42.2	10	1	1	0
226	Penner	10924	162	676	676	676	676	676	676	676	676	266	120	194	512	0	1	2	9
227	Volta	19863	1.9	676	78.7	14.1	3.61	1.67	1.03	0.34	0.283	0.944	1.43	2.39	65.2	11	0	0	1
228	Lempa	4213	4.74	676	676	676	258	2.81	0.913	0.737	0.424	0.677	3.3	8.11	192	8	0	0	4
229	Gambia	1355	0.22	676	676	676	676	1	0.595	0.139	0.118	0.413	0.21	0.344	225	8	0	0	4
230	Grande De Matagalpa	547	0.158	3.39	32.6	78.4	20.9	0.149	0.118	0.249	0.0814	0.0468	0.0571	0.21	11.4	12	0	0	0
231	Cauvery	35203	110	648	669	661	630	208	206	228	281	165	101	126	336	0	3	1	8
232	San Juan	3736	0.924	8.57	33.1	53.1	4.74	0.534	0.596	0.957	0.503	0.251	0.346	0.8	8.7	12	0	0	0
233	Geba	388	3.22	676	676	676	676	10.5	0.451	0.0222	0.0606	0.132	2.54	5.7	227	8	0	0	4
234	Corubal	540	0.219	676	676	676	676	0.404	0.0306	0.0125	0.0176	0.0341	0.171	0.385	225	8	0	0	4
235	Magdalena	25486	0.682	5.88	8.48	4.3	2.98	3.86	9.22	10.4	3.4	0.736	0.57	0.908	4.28	12	0	0	0
236	Comoe	2531	4.16	676	676	18.1	4.73	1.55	1.96	1.14	0.712	2	4.1	9.31	117	10	0	0	2
237	Orinoco	12008	0.348	3.79	4.59	1.21	0.304	0.205	0.27	0.424	0.388	0.141	0.174	0.669	1.04	12	0	0	0
238	Bandama	4222	1.35	676	676	28.3	8.24	0.559	0.843	0.252	0.109	0.398	2.02	4.88	117	10	0	0	2
239	Oueme	5845	1.05	676	676	83.2	1.79	0.332	0.258	0.23	0.168	0.283	0.958	1.99	120	10	0	0	2
240	Sassandra	3066	0.26	676	676	14	3.53	0.165	0.0842	0.0627	0.0329	0.0779	0.387	1.09	114	10	0	0	2
241	Shebelle	16004	34.9	612	356	3.68	5.38	69.1	68.3	30.8	12.3	11.3	8.99	30.4	104	10	0	0	2
242	Mono	1580	1.64	676	14.9	3.07	1.13	0.39	0.355	0.433	0.272	0.422	1.05	2.1	58.5	11	0	0	1
243	Congo	67996	0.0191	0.0519	0.0391	0.0337	0.104	0.256	0.302	0.247	0.181	0.112	0.0369	0.0235	0.117	12	0	0	0
244	Atrato	511	0.0335	0.13	0.113	0.0791	0.0551	0.0506	0.0498	0.049	0.0445	0.0417	0.0423	0.0538	0.0619	12	0	0	0
245	Cuyuni	145	0.0096	0.0327	0.0457	0.0286	0.0098	0.0064	0.0067	0.0099	0.02	0.0294	0.0274	0.0152	0.0201	12	0	0	0

Basin ID	Basin name	Population (thousands)	Water scarcity (%)													Number of months per year that a basin faces low, moderate, significant or severe water scarcity			
			Jan	Feb	Mar	Apr	May	Jun	Jul	Aug	Sep	Oct	Nov	Dec	Average	Low	Moderate	Significant	Severe
246	Cavally	1043	0.049	1.25	0.767	0.261	0.0524	0.0199	0.0316	0.0449	0.02	0.0227	0.0385	0.0817	0.22	12	0	0	0
247	Tano	1228	0.322	676	3.18	0.447	0.141	0.0551	0.111	0.252	0.197	0.0975	0.191	0.461	56.8	11	0	0	1
248	Cross	8986	0.199	676	1.42	0.575	0.271	0.131	0.0782	0.067	0.0526	0.0567	0.155	0.338	56.6	11	0	0	1
249	Sanaga	3878	0.169	346	2.85	0.259	0.0698	0.0455	0.0294	0.0231	0.0163	0.019	0.134	0.325	29.2	11	0	0	1
250	Pra	4090	5.99	676	17.4	2.37	0.669	0.235	0.705	1.78	0.662	0.234	0.504	4.25	59.2	11	0	0	1
251	Davo	588	0.662	676	676	50.1	5.49	0.078	0.189	0.638	0.309	0.247	0.381	1.33	118	10	0	0	2
252	Essequibo	54.1	0.0021	0.0053	0.0049	0.0035	0.0015	0.0008	0.0009	0.0013	0.0026	0.0043	0.0055	0.0033	0.003	12	0	0	0
253	Kelantan	628	5.48	33.8	2.62	1.41	1.82	3.3	4.68	4.49	11.4	3.87	1.72	0.896	6.29	12	0	0	0
254	Corantijn	115	0.0204	0.0323	0.0157	0.0075	0.0032	0.0022	0.008	0.0205	0.153	0.151	0.0883	0.0493	0.046	12	0	0	0
255	Coppename	14.2	0.0032	0.0036	0.0033	0.0024	0.0012	0.0011	0.0013	0.0022	0.0043	0.0072	0.012	0.0157	0.0048	12	0	0	0
256	Kinabatangan	230	0.0417	0.117	0.149	0.152	0.156	0.0914	0.133	0.112	0.11	0.0911	0.0927	0.0615	0.109	12	0	0	0
257	Maroni	35.4	0.0011	0.001	0.0008	0.0006	0.0004	0.0004	0.0006	0.001	0.002	0.0032	0.0054	0.0078	0.002	12	0	0	0
258	San Juan (Columbia - P	480	0.0817	0.291	0.544	0.355	0.339	0.432	0.808	0.802	0.306	0.118	0.0962	0.127	0.358	12	0	0	0
259	Amazonas	24647	0.0474	0.0583	0.0602	0.146	0.2	0.193	0.226	0.479	0.606	0.422	0.249	0.0907	0.232	12	0	0	0
260	Pahang	1772	1.85	3.78	0.696	0.554	0.622	1.8	3.95	3.86	7.13	1.92	1.3	0.756	2.35	12	0	0	0
261	Nyong	1097	0.0496	676	0.162	0.0543	0.0344	0.0416	0.0896	0.0923	0.0257	0.0184	0.0368	0.0753	56.4	11	0	0	1
262	Oyapock	10.6	0.0007	0.0008	0.0006	0.0005	0.0005	0.0005	0.0009	0.0015	0.0027	0.0045	0.0075	0.0053	0.0022	12	0	0	0
263	Rajang	318	0.0337	0.0236	0.0143	0.0135	0.0142	0.0178	0.0216	0.0217	0.0348	0.0204	0.0273	0.0434	0.0239	12	0	0	0
264	Ntem	406	0.0443	11.1	0.204	0.0553	0.0345	0.0495	0.113	0.19	0.0574	0.0202	0.028	0.0631	0.99	12	0	0	0
265	Ogooue	606	0.0132	0.0361	0.0152	0.0073	0.0069	0.0232	0.0773	0.207	0.168	0.0276	0.0052	0.0097	0.0497	12	0	0	0
266	Rio Araguari	30	0.0026	0.0023	0.0018	0.0015	0.0015	0.0017	0.0029	0.005	0.009	0.0149	0.0247	0.0311	0.0083	12	0	0	0
267	Mira	617	1.37	1.82	1.3	0.962	0.937	1.46	5.04	10.1	7.03	2.62	2.15	1.85	3.05	12	0	0	0
268	Esmeraldas	2571	2.48	1.32	0.74	0.564	0.941	2.31	10	30.5	33	15.5	16.1	6.74	10	12	0	0	0
269	Tana	4247	19.2	376	48.1	0.977	0.652	4.16	17	35.9	57.1	23.4	2.44	6.91	49.3	11	0	0	1
270	Daule & Vinces	3752	14	3.34	1.48	1.11	4.48	16.4	60.7	140	159	85.5	144	89.1	59.9	9	2	1	0
271	Rio Gurupi	224	0.203	0.0453	0.0199	0.0206	0.0281	0.0531	0.0896	0.175	0.289	0.433	0.655	0.97	0.248	12	0	0	0
272	Rio Capim	571	0.157	0.042	0.0268	0.0297	0.04	0.0635	0.0931	0.159	0.298	0.485	0.804	1.18	0.282	12	0	0	0
273	Tocantins	4744	0.106	0.0878	0.0751	0.219	0.276	0.594	1.18	2.14	2.61	1.4	0.328	0.13	0.763	12	0	0	0
274	Kouilou	807	0.01	0.0168	0.01	0.0074	0.0536	0.714	1.69	3.47	5.87	7.51	0.282	0.0131	1.64	12	0	0	0
275	Nyanga	30.5	0.0054	0.0091	0.0067	0.006	0.012	0.0235	0.0388	0.0643	0.106	0.176	0.0091	0.0078	0.0388	12	0	0	0
276	Rio Parnaiba	3700	1.8	0.768	0.318	0.478	2.22	5.8	11	20.1	30.9	42.7	41.6	10.4	14	12	0	0	0
277	Rio Itapecuru	971	6.16	0.375	0.132	0.15	0.558	1.39	2.6	4.43	6.43	8.56	9.15	12.5	4.37	12	0	0	0
278	Rio Acarau	462	15.9	12.7	0.359	0.223	0.774	4.41	8.58	17.5	32.2	48.1	67.4	89	24.8	12	0	0	0
279	Pangani	2174	88.2	473	295	33.3	24.1	120	223	203	260	287	123	108	186	3	3	0	6
280	Rio Pindare	517	0.926	0.0941	0.0455	0.0482	0.0861	0.187	0.344	0.588	0.967	1.51	2.25	3.63	0.89	12	0	0	0
281	Sepik	785	0.0022	0.0036	0.0027	0.0028	0.0037	0.0046	0.0051	0.0051	0.0045	0.0043	0.0043	0.0038	0.0039	12	0	0	0
282	Rio Mearim	932	1.48	0.162	0.0757	0.102	0.256	0.642	1.18	2.02	3.25	4.82	5.71	8.71	2.37	12	0	0	0
283	Chira	651	175	33.9	6.34	8.78	40.3	68.8	165	306	369	356	493	526	212	5	0	2	5
284	Rufiji	4582	2.44	1.05	0.402	0.851	4.41	4.47	5.19	7.77	12	16.8	13.6	6.85	6.33	12	0	0	0
285	Rio Jaguaribe	2097	68.8	467	2.43	1.7	7.98	20.6	38.3	76.3	133	183	219	253	123	7	1	1	3
286	Purari	797	0.0047	0.008	0.0064	0.0063	0.0075	0.0095	0.0113	0.0109	0.0087	0.0092	0.0092	0.0079	0.0083	12	0	0	0
287	Ruvu	699	15.2	8.13	2.09	0.422	1.89	4.08	6.92	13.5	21	28.3	28.9	12.1	11.9	12	0	0	0
288	Rio Paraiba	1212	32.7	676	420	12.4	5.8	2.89	5.2	14.3	49.8	79.9	110	137	129	8	2	0	2
289	Solo (Bengawan Solo)	11103	58.4	20	7.93	0.899	2.2	14.3	46.6	108	230	242	549	78.4	113	8	1	0	3
290	Sao Francisco	12443	0.497	1.22	2.07	6.3	13	12.2	13.3	25.3	53.4	47.6	5.91	1.07	15.2	12	0	0	0
291	Brantas	8996	50.2	15	5.46	1.01	2.07	11	43	107	216	199	528	96.7	106	8	1	1	2
292	Santa	452	4.88	4.19	4.5	22.4	43.7	67	89	189	276	76.9	34.9	12.6	68.7	10	0	1	1
293	Zambezi	31680	0.129	0.11	0.233	1.32	3.22	5.67	11.2	25.5	49.8	67.5	38.9	0.789	17	12	0	0	0
294	Rio Vaza-Barris	422	47.1	676	676	676	15.4	5.34	5.11	11.3	31.3	47.9	55.3	78.5	194	9	0	0	3
295	Rio Itapicuru	958	23.3	676	676	220	16.4	5.42	2.56	5.76	16.4	27.7	32.5	47.1	146	9	0	0	3
296	Rio Paraguacu	1629	7.67	14	10.9	8.51	5.14	5.21	4.12	10.1	22.5	31.1	21.4	20.4	13.4	12	0	0	0
297	Canete	121	4.93	3.81	7.01	25.8	56.3	67.2	78.7	152	214	65.2	34.9	13.1	60.3	10	0	1	1
298	Rio De Contas	1402	9.89	26.4	19.4	15	20.5	20.9	25.2	57.5	103	115	29.9	16.8	38.3	10	2	0	0
299	Roper	4.05	0.0116	0.0028	0.0321	0.552	1.39	2.17	3.8	7.28	12.9	18.7	12.2	3.58	5.22	12	0	0	0
300	Daly	14.9	0.0208	0.0057	0.0298	0.488	1.23	1.98	3.45	6.31	11.1	15.2	7.8	2.28	4.16	12	0	0	0
301	Drysdale	2.15	0.0833	0.0077	0.0081	0.0227	0.0375	0.0621	0.103	0.17	0.282	0.466	0.771	1.28	0.274	12	0	0	0
302	Parana	67514	1.76	1.93	1.7	3.09	2.81	4.09	8.66	13.4	11.3	7.19	4.29	1.83	5.17	12	0	0	0
303	Durack	2.23	116	1.22	1.38	3.81	6.28	10.3	16.9	27.6	44.5	70.6	109	164	47.7	9	2	1	0
304	Rio Prado	613	0.608	2.02	1.74	2.09	4.44	4.9	6.49	15.4	29.1	36.6	4.83	0.881	9.09	12	0	0	0
305	Victoria	1.37	9.29	0.0681	0.099	0.255	0.421	0.697	1.15	1.91	3.15	5.2	8.57	14.1	3.74	12	0	0	0
306	Mitchell(N. Au)	24.4	0.158	0.021	0.0625	0.757	1.93	3.14	5.61	10.9	20.8	33.4	33.3	23.2	11.1	12	0	0	0
307	Majes	103	1.85	1.17	1.27	7.16	14.1	11.9	13.1	32.4	68.6	67.6	60.3	3.74	23.6	12	0	0	0

Basin ID	Basin name	Population (thousands)	Water scarcity (%)													Number of months per year that a basin faces low, moderate, significant or severe water scarcity			
			Jan	Feb	Mar	Apr	May	Jun	Jul	Aug	Sep	Oct	Nov	Dec	Average	Low	Moderate	Significant	Severe
308	Ord	2.47	187	0.647	31.5	493	604	641	659	667	671	672	672	637	495	2	0	1	9
309	Jequitinhonha	887	0.112	0.451	0.544	1.16	2.24	3.57	5.87	11.7	20	17.9	0.81	0.136	5.37	12	0	0	0
310	Macarthur	0.542	1.4	0.512	0.0099	0.038	0.0629	0.104	0.172	0.285	0.472	0.781	1.29	2.14	0.606	12	0	0	0
311	Fitzroy	5.86	1.09	0.0134	0.0126	0.0422	0.07	0.114	0.194	0.335	0.565	0.942	1.47	2.1	0.579	12	0	0	0
312	Gilbert	2.71	0.0201	0.0025	0.0307	0.224	0.375	0.538	0.949	1.89	3.59	5.9	6.63	5.68	2.15	12	0	0	0
313	Mucuri	300	0.15	0.846	1.15	1.76	3.12	5.59	7.9	19.9	30.9	31	1.26	0.18	8.65	12	0	0	0
314	Rio Doce	3858	0.16	1.08	1.22	2.65	5.78	12.6	26.1	49.6	65.2	80.5	1.3	0.203	20.5	12	0	0	0
315	Save	3185	1.6	1.09	2.55	10.7	14.4	27.8	52.5	150	254	284	190	10.8	83.2	8	1	1	2
316	Burdekin	69.3	0.775	0.145	0.83	4.24	6.71	10.9	21.5	43	82.6	129	143	86.9	44.1	10	2	0	0
317	Tsiribihina	2397	3.15	2.16	4.86	20.5	12.2	1.27	1.99	3.38	5.85	10	4.64	3.45	6.12	12	0	0	0
318	Buzi	1034	0.13	0.044	0.151	1.3	2.71	4.31	7.5	18.7	39.3	60.4	41.7	3.13	15	12	0	0	0
319	Loa	196	674	472	608	632	648	659	666	670	672	674	674	675	644	0	0	0	12
320	Limpopo	15637	26.3	20.3	39.2	74.6	78.3	125	211	374	492	527	454	147	214	5	2	0	5
321	De Grey	5.41	673	674	674	674	675	676	676	676	676	676	676	674	675	0	0	0	12
322	Paraiba Do Sul	6928	0.548	0.962	1.29	3.25	4.59	8.69	17.3	30.6	28.1	12.2	2.72	0.828	9.26	12	0	0	0
323	Fortescue	4.79	675	674	674	675	675	676	676	676	676	676	676	674	675	0	0	0	12
324	Mangoky	587	9.91	6.74	13.8	34.7	18.6	5.28	6.91	11.2	18.1	32.9	19.9	19.1	16.4	12	0	0	0
325	Fitzroy	150	42.3	2.53	10.9	26.7	31.1	37.9	75.7	148	261	356	392	412	150	7	1	0	4
326	Orange	12666	32.9	49.7	53.5	61.9	51.6	102	186	306	383	324	135	62.5	146	6	2	1	3
327	Ashburton	4.97	673	673	672	674	675	675	676	675	676	676	676	675	675	0	0	0	12
328	Gascoyne	2.33	675	675	675	675	676	675	676	675	676	676	676	676	675	0	0	0	12
329	Rio Ribeira Do Iguape	2463	0.495	0.625	0.752	1.33	1.42	1.31	1.94	2.43	1.75	1.21	1.39	1.09	1.31	12	0	0	0
330	Incomati	2416	3.24	4.49	8.66	28	36.3	60.5	108	211	328	348	64.5	11.3	101	8	1	0	3
331	Murray	2348	313	591	559	383	110	32.9	29.4	46.1	84	135	216	306	234	4	2	0	6
332	Murchison	4.82	675	676	675	675	676	675	676	675	676	676	676	676	675	0	0	0	12
333	Maputo	1265	0.896	1.3	5.18	24.9	42.9	77.9	128	248	351	403	61	10.6	113	8	1	0	3
334	Uruguay	5047	21.5	26.9	6.48	0.327	0.133	0.118	0.143	0.22	0.187	1.88	8.66	18	7.04	12	0	0	0
335	Tugela	1784	8.46	20.5	34.3	42.8	43.9	71.9	142	258	341	322	148	17.7	121	7	2	0	3
336	Colorado (Argentinia)	3268	23.5	185	234	168	47.2	19.5	37.5	63.7	93.6	44.8	36.5	29.4	81.9	9	0	2	1
337	Rio Jacui	2578	25.9	21.9	8.23	0.301	0.218	0.184	0.199	0.209	0.191	2.74	12.1	23.5	7.97	12	0	0	0
338	Huasco	26	4.78	20.3	29.5	26.4	31.6	78.3	132	312	474	552	482	7.38	179	7	1	0	4
339	Limari	142	140	101	186	103	98.2	20.2	36.3	140	384	510	539	267	210	3	4	1	4
340	Negro (Uruguay)	531	29.8	231	25.1	0.568	0.0372	0.0255	0.0262	0.0263	0.0275	0.409	5.78	19.1	26	11	0	0	1
341	Groot-Vis	299	676	676	676	676	676	676	676	676	676	676	676	676	676	0	0	0	12
342	Salado	1880	15.2	642	87.3	2.65	1.13	1.09	1.42	1.74	2.56	2.25	2.5	5.01	63.7	11	0	0	1
343	Blackwood	27.7	4.26	676	676	676	676	0.97	0.111	0.0761	0.144	1.2	3.58	7.41	227	8	0	0	4
344	Rapel	740	71.9	259	281	108	2.62	0.438	0.44	1.39	14.4	54.4	68.4	66.3	77.3	9	1	0	2
345	Negro (Argentinia)	710	4.62	115	24.2	3.77	0.416	0.145	0.18	0.406	0.864	1.5	3.32	6.65	13.4	11	1	0	0
346	Biobio	655	9.39	259	19.4	2.13	0.782	0.597	0.587	0.661	1.44	2.67	1.63	7.51	25.5	11	0	0	1
347	Waikato	323	0.333	1.01	1.92	0.778	0.304	0.235	0.238	0.248	0.278	0.285	0.367	0.552	0.546	12	0	0	0
348	South Esk	55.7	11.7	434	428	53.5	6.91	0.581	0.145	0.399	2.12	5.05	9.63	21.8	81.1	10	0	0	2
349	Chubut	212	2.73	65.3	31	9.64	1.26	0.277	0.305	0.604	1.43	3.24	5.8	9.35	10.9	12	0	0	0
350	Clutha	33.9	1.23	20.5	18.4	8.09	1.19	0.147	0.147	0.639	3.97	5.48	8.68	8.15	6.39	12	0	0	0
351	Baker	14.5	0.0066	0.0526	0.049	0.0197	0.0075	0.0053	0.0053	0.0073	0.0163	0.0364	0.0388	0.0435	0.024	12	0	0	0
352	Santa Cruz	10	0.0088	0.178	0.157	0.0403	0.0109	0.005	0.0057	0.0075	0.0142	0.0281	0.0656	0.0797	0.05	12	0	0	0
353	Ganges	454094	204	639	605	482	389	99.6	25.1	9.27	18.9	78.7	174	172	241	5	0	2	5
354	Salween	6599	1.13	111	29.4	24.2	12.5	3.11	0.812	0.499	0.866	1.28	1.03	1.22	15.6	11	1	0	0
355	Hong(Red River)	25632	9.08	588	509	427	165	17.3	3.12	1.98	2.34	1.86	3.61	8.77	145	8	0	1	3
356	Lake Chad	34285	9.01	673	674	535	86.7	19.3	2.14	0.335	0.622	2.04	3.05	7.09	168	9	0	0	3
357	Okavango	1774	0.104	0.0724	0.0788	0.292	0.663	1.13	2.13	4.53	8.72	13.4	11.7	0.835	3.64	12	0	0	0
358	Tarim	9311	95.8	673	675	670	413	250	231	293	363	212	163	119	346	1	1	1	9
359	Horton	0.036	0.0097	0.375	0.621	1.03	0.0015	0.0004	0.0013	0.0022	0.0036	0.006	0.0099	0.0164	0.173	12	0	0	0
360	Hornaday	0.054	0.0145	25.7	41.5	66.1	103	0.001	0.0012	0.0026	0.0049	0.0081	0.0134	0.0222	19.7	11	1	0	0
361	Conception	193	676	676	676	676	676	676	676	676	676	676	676	676	676	0	0	0	12
362	Ulua	2716	2.13	33.2	137	245	251	5.9	1.03	0.65	0.244	0.159	0.32	2.11	56.6	9	1	0	2
363	Patacua	538	0.2	1.34	8.94	21.8	18.4	2.84	0.373	0.162	0.1	0.0343	0.0465	0.223	4.53	12	0	0	0
364	Coco	694	0.125	2.03	8.35	17.2	5.8	0.0852	0.0433	0.0463	0.039	0.0284	0.0418	0.125	2.83	12	0	0	0
365	Ocona	68.3	1.29	1.14	1.41	11.4	22.4	17.3	17.1	41.9	87.2	32.6	17.4	3.29	21.2	12	0	0	0
366	Cuanza	2845	0.0419	0.0779	0.0359	0.0377	0.184	0.632	1.28	3.07	5.74	8.22	3.27	0.146	1.89	12	0	0	0
367	Cunene	1370	0.0505	0.0418	0.0165	0.0367	0.146	0.304	0.568	1.1	1.86	2.11	1.53	0.154	0.66	12	0	0	0
368	Doring	167	153	676	676	676	676	13.1	7.01	19	81	214	264	326	315	4	0	1	7
369	Gamka	279	20.8	454	307	158	90.1	60.8	50.2	45	70.2	107	72.7	105	128	7	2	1	2

Basin ID	Basin name	Population (thousands)	Water scarcity (%)													Number of months per year that a basin faces low, moderate, significant or severe water scarcity			
			Jan	Feb	Mar	Apr	May	Jun	Jul	Aug	Sep	Oct	Nov	Dec	Average	Low	Moderate	Significant	Severe
370	Groot- Kei	874	454	456	205	192	286	378	491	575	628	648	612	569	458	0	0	1	11
371	Lurio	1250	0.0046	0.0034	0.0035	0.0131	0.0276	0.0455	0.0813	0.165	0.315	0.525	0.521	0.0528	0.146	12	0	0	0
372	Messalo	288	0.0053	0.0027	0.0023	0.0047	0.0139	0.0233	0.0643	0.171	0.382	0.633	0.257	0.34	0.158	12	0	0	0
373	Rovuma	1994	0.0202	0.0091	0.0065	0.0204	0.0656	0.0789	0.119	0.229	0.409	0.688	0.773	0.321	0.228	12	0	0	0
374	Galana	5589	10.9	282	30.5	0.928	1	4.53	13.5	24.6	38.1	55	4.45	5.36	39.2	11	0	0	1
375	Pyasina	244	0.492	47.1	74.6	115	171	0.027	0.0818	0.123	0.128	0.284	0.47	0.778	34.2	10	1	1	0
376	Popigay	0.845	0.0094	0.967	1.6	2.64	4.37	0.0004	0.0011	0.0019	0.0032	0.0054	0.009	0.0149	0.802	12	0	0	0
377	Fuchun Jiang	10914	3.74	2.39	1.45	2.37	3.9	2.32	24.9	32.7	38.5	11.5	7.38	8.23	11.6	12	0	0	0
378	Min Jiang	9730	2.39	1.8	0.633	0.7	0.765	0.798	9.51	9.53	10.9	2.79	3.48	4.78	4.01	12	0	0	0
379	Han Jiang	9672	9.79	21.7	3.31	2.36	2.35	2.3	12.3	9.29	12.4	6.61	9.9	15.2	8.96	12	0	0	0
380	Mamberamo	442	0.004	0.0066	0.0049	0.0054	0.0063	0.0079	0.0073	0.0078	0.0073	0.0097	0.0088	0.0072	0.0069	12	0	0	0
381	Lorentz	16.1	0.0045	0.0051	0.0044	0.0048	0.0065	0.0089	0.0081	0.009	0.0068	0.0118	0.0083	0.0086	0.0072	12	0	0	0
382	Eilanden	55.7	0.0015	0.0025	0.0021	0.0021	0.0022	0.0024	0.0025	0.0026	0.0025	0.0031	0.0031	0.0025	0.0024	12	0	0	0
383	Uwimbu	58.8	0.0009	0.0017	0.0015	0.0015	0.0019	0.0027	0.0036	0.0041	0.0038	0.0038	0.0032	0.0019	0.0026	12	0	0	0
384	Sungai Kajan	93	0.0192	0.0152	0.0025	0.0021	0.002	0.0025	0.003	0.0029	0.0432	0.0315	0.0077	0.0202	0.0127	12	0	0	0
385	Sungai Mahakam	892	0.0431	0.0521	0.0142	0.0097	0.0117	0.0144	0.0369	0.0538	0.284	0.152	0.0606	0.0393	0.0644	12	0	0	0
386	Sungai Kapuas	1607	0.0191	0.0289	0.0162	0.0147	0.0171	0.0227	0.034	0.0372	0.0618	0.0271	0.0187	0.0186	0.0263	12	0	0	0
387	Batang Kuantan	1520	2.82	3.06	0.24	0.208	0.315	1.04	2.32	2.91	9.91	5.1	2.78	1.94	2.72	12	0	0	0
388	Batang Hari	2049	0.861	0.929	0.0969	0.0792	0.168	0.493	0.963	1.4	3.64	2.02	1.01	0.65	1.03	12	0	0	0
389	Flinders	6.33	16.4	0.148	2.31	6.2	9.4	12.9	23.2	45.3	83.9	129	149	159	53.1	9	2	1	0
390	Leichhardt	6.43	0.206	0.198	1.01	2.22	3.58	5.33	9.29	17.1	30.5	48.6	63.5	76.8	21.5	12	0	0	0
391	Escaut (Schelde)	9448	10.6	20.2	23.7	30.5	56	99.7	164	244	292	227	38.3	19.2	102	8	0	1	3
392	Issyk-Kul	3325	6.44	39.8	1.32	12.6	38.6	69.1	109	204	227	165	31.1	11.4	76.3	8	1	1	2
393	Balkhash	5182	14.4	676	6.01	21.7	45	77.6	157	262	300	180	17.5	23.2	148	7	0	2	3
394	Eyre Lake	86.2	674	675	675	676	676	676	676	676	676	676	676	675	675	0	0	0	12
395	Lake Mar Chiquita	4097	75.1	61.2	24.5	42.4	41.9	74.3	155	269	411	324	235	113	152	6	1	1	4
396	Lake Turkana	8701	4.01	676	34.7	0.5	0.4	0.287	0.234	0.472	0.639	0.762	0.99	3.03	60.1	11	0	0	1
397	Dead Sea	6150	4.11	7.78	51.5	214	372	444	531	574	568	569	532	68.6	328	4	0	0	8
398	Suriname	103	0.0166	0.019	0.0174	0.0125	0.0077	0.0073	0.0092	0.015	0.03	0.0499	0.0826	0.0691	0.028	12	0	0	0
399	Lake Titicaca	2691	3.83	3.34	3.06	4.28	4.71	7.19	9.49	14.4	18.3	13.5	15.4	5.87	8.62	12	0	0	0
400	Lake Vattern	405	3.29	676	0.556	0.671	1.73	3.65	7.32	11.3	11.9	4.4	1.83	4.19	60.5	11	0	0	1
401	Great Salt Lake	2224	86.4	23.9	37.9	69.3	104	229	369	422	418	409	235	99.9	209	5	1	0	6
402	Lake Taymur	6.19	0.0081	157	226	307	391	0.0004	0.0009	0.0018	0.0023	0.0045	0.0075	0.0124	90	8	0	1	3
403	Daryacheh-Ye Orumieh	4307	13.2	37.6	33.2	27.4	32.8	90.7	173	267	271	206	65.4	32.5	104	8	0	1	3
404	Van Golu	894	3.59	676	3.91	0.808	3.18	18.9	30	51.2	59.5	20	3.52	6.18	73	11	0	0	1
405	Ozero Sevan	412	58.7	676	144	7.74	8.36	42	105	175	160	79	40.2	88.2	132	7	2	2	1
